# Nanopore sequencing with unique molecular identifiers enables accurate mutation analysis and haplotyping in the complex lipoprotein(a) KIV-2 VNTR

**DOI:** 10.1186/s13073-024-01391-8

**Published:** 2024-10-08

**Authors:** Stephan Amstler, Gertraud Streiter, Cathrin Pfurtscheller, Lukas Forer, Silvia Di Maio, Hansi Weissensteiner, Bernhard Paulweber, Sebastian Schönherr, Florian Kronenberg, Stefan Coassin

**Affiliations:** 1 Institute of Genetic Epidemiology, Medical University of Innsbruck, Innsbruck, Austria; 2 Department of Internal Medicine I, Paracelsus Medical University, Salzburg, Austria

**Keywords:** Lipoprotein(a), Copy number variation, CNV, Variable number tandem repeat, VNTR, Nanopore sequencing, UMI, Unique molecular identifiers, Molecular barcodes, DNA sequencing

## Abstract

**Background:**

Repetitive genome regions, such as variable number of tandem repeats (VNTR) or short tandem repeats (STR), are major constituents of the uncharted dark genome and evade conventional sequencing approaches. The protein-coding *LPA* kringle IV type-2 (KIV-2) VNTR (5.6 kb per unit, 1–40 units per allele) is a medically highly relevant example with a particularly intricate structure, multiple haplotypes, intragenic homologies, and an intra-VNTR STR. It is the primary regulator of plasma lipoprotein(a) [Lp(a)] concentrations, an important cardiovascular risk factor. Lp(a) concentrations vary widely between individuals and ancestries. Multiple variants and functional haplotypes in the *LPA* gene and especially in the KIV-2 VNTR strongly contribute to this variance.

**Methods:**

We evaluated the performance of amplicon-based nanopore sequencing with unique molecular identifiers (UMI-ONT-Seq) for SNP detection, haplotype mapping, VNTR unit consensus sequence generation, and copy number estimation via coverage-corrected haplotypes quantification in the KIV-2 VNTR. We used 15 human samples and low-level mixtures (0.5 to 5%) of KIV-2 plasmids as a validation set. We then applied UMI-ONT-Seq to extract KIV-2 VNTR haplotypes in 48 multi-ancestry 1000 Genome samples and analyzed at scale a poorly characterized STR within the KIV-2 VNTR.

**Results:**

UMI-ONT-Seq detected KIV-2 SNPs down to 1% variant level with high sensitivity, specificity, and precision (0.977 ± 0.018; 1.000 ± 0.0005; 0.993 ± 0.02) and accurately retrieved the full-length haplotype of each VNTR unit. Human variant levels were highly correlated with next-generation sequencing (*R*^2^ = 0.983) without bias across the whole variant level range. Six reads per UMI produced sequences of each KIV-2 unit with Q40 quality. The KIV-2 repeat number determined by coverage-corrected unique haplotype counting was in close agreement with droplet digital PCR (ddPCR), with 70% of the samples falling even within the narrow confidence interval of ddPCR. We then analyzed 62,679 intra-KIV-2 STR sequences and explored KIV-2 SNP haplotype patterns across five ancestries.

**Conclusions:**

UMI-ONT-Seq accurately retrieves the SNP haplotype and precisely quantifies the VNTR copy number of each repeat unit of the complex KIV-2 VNTR region across multiple ancestries. This study utilizes the KIV-2 VNTR, presenting a novel and potent tool for comprehensive characterization of medically relevant complex genome regions at scale.

**Supplementary Information:**

The online version contains supplementary material available at 10.1186/s13073-024-01391-8.

## Background

Complex genome regions such as copy number variations (CNVs), variable number of tandem repeats (VNTR), short tandem repeats (STRs), and centromeric and telomeric regions can hide medically highly relevant mutations that shed new light on human phenotypes and diseases [1–6]. The development of long-read sequencing technologies and new bioinformatic tools have greatly improved the resolution of these difficult regions [7–14]. However, some overly complex repeat structures remain challenging. The *LPA* KIV-2 VNTR is a medically highly relevant protein-coding example of such a difficult region [15].

The *LPA* gene encodes the apolipoprotein(a) [apo(a)] and controls most (> 90%) of the lipoprotein(a) [Lp(a)] plasma variation [16]. High Lp(a) plasma concentrations are considered a nearly monogenically determined, very frequent, causal, independent, and heritable risk factor for atherosclerotic cardiovascular diseases [17–19] that increase cardiovascular risk up to threefold [20, 21]. Elevated Lp(a) concentrations are found in ≈20% of individuals of European ancestry and even in ≈50% of individuals of African ancestry [17]. Median Lp(a) levels vary tenfold between ancestries [22] and the individual plasma concentrations vary even 1000-fold [16]. The causes of this huge phenotypic variance are not fully understood but likely result from intricate, haplotype-dependent, non-linear interactions between multiple functional *LPA* variants and the KIV-2 VNTR size [15].

The complex structure of the *LPA* gene severely complicates genetic analysis [15]. It comprises ten highly homologous kringle-IV domains (KIV-1 to -10) [23, 24]. Each KIV domain consists of two short exons (≈160 and 182 bp) interspaced by a mostly ≈4 kb intra-kringle intron and a 1.2 kb intron linking it to the next domain [15]. The KIV-2 domain is encoded by a polymorphic VNTR, which introduces 1 to ≈40 KIV-2 units per gene allele (5.6 kb per repeat unit) [23]. This creates an up to > 200 kb VNTR region consisting of highly homologous coding repeat units that encompass up to 70% of the protein [25]. The VNTR copy number explains 30–70% of Lp(a) variance in a non-linear, ancestry-dependent manner [16]. Individuals carrying at least one low molecular weight (LMW) apo(a) isoform (defined as 10–22 KIV units [16], i.e., 1 to 13 KIV-2 units [15]) present 5 to 10 times higher median Lp(a) levels compared to high molecular weight isoforms (> 22 KIV; HMW) due to higher protein production rates [17]. However, the individual Lp(a) levels within the same isoforms can still vary 10- to 200-fold [15] due to multiple, partially unknown genetic variants that modify the effect of the VNTR [15].

The interactions between the KIV-2 VNTR size and modifier SNPs are complex and multilayered (reviewed in detail in [15]). They are haplotype-dependent and only partially captured by linkage disequilibrium (LD) [15]. Several functional SNPs, including the two SNPs (4925G > A [25] and 4733G > A [26]) that explain most of Lp(a) variance [25, 26], have been hidden until recently in the KIV-2 VNTR [25–28]. These two variants alone explain remarkable 11.9% of the Lp(a) variance in the general population, are ancestry-specific, are associated with considerably reduced cardiovascular risk, and were hidden in the KIV-2 VNTR until recently [25–28]. The background apo(a) isoform size and other variants on the same haplotype can both limit and amplify the effects of any functional variant [15]. Although the KIV-2 VNTR encompasses most of the *LPA* gene region, the full genetic and haplotypic diversity of KIV-2 units and the LD of KIV-2 haplotypes with the haplotypes in the non-repetitive kringles remain largely unknown.

Current short-read deep sequencing approaches confidently identify KIV-2 SNPs [24], but mostly lose the long-range SNP haplotype data. Early cloning studies identified three synonymous KIV-2 haplotypes named KIV-2A, KIV-2B, and KIV-2C [29, 30]. These KIV-2 subtypes are defined by the haplotype of three SNPs in KIV-2 exon 1 and differ by about 120 bases [23, 24]. The KIV-2 subtypes have no functional relevance, but their frequencies differ widely between ancestries and correlate with known differences of the Lp(a) phenotype across ancestries [24, 30]. They may thus reflect distinct evolutionary histories of the KIV-2 region and tag novel ancestry-specific functional variants. Further haplotypic effects in the KIV-2 VNTR are unknown and could not be studied so far.

Nanopore sequencing (ONT-Seq; Oxford Nanopore Technologies, ONT) provides novel means to address this knowledge gap. DNA is sequenced by monitoring the sequence-specific current fluctuations generated by single-stranded DNA molecules translocating through protein pores [31, 32]. This generates hundred times longer read lengths than short-read next-generation sequencing (NGS) [14, 32] and provides single molecule resolution. It thus allows retrieving the complete haplotype of each DNA molecule sequenced, even in DNA mixtures [32]. However, at the single molecule level the benefits of nanopore sequencing are limited by its relatively high raw-read error rate (0.7–1% median error rate per read). Especially in highly similar repeat sequences like the KIV-2, errors cannot be polished by sequencing deeper (because the parental repeat of each read is unknown [33]) or by using double-stranded (“duplex”) basecalling (because of erroneous matching of strands originating from different parental molecules).

**Fig. 1:**
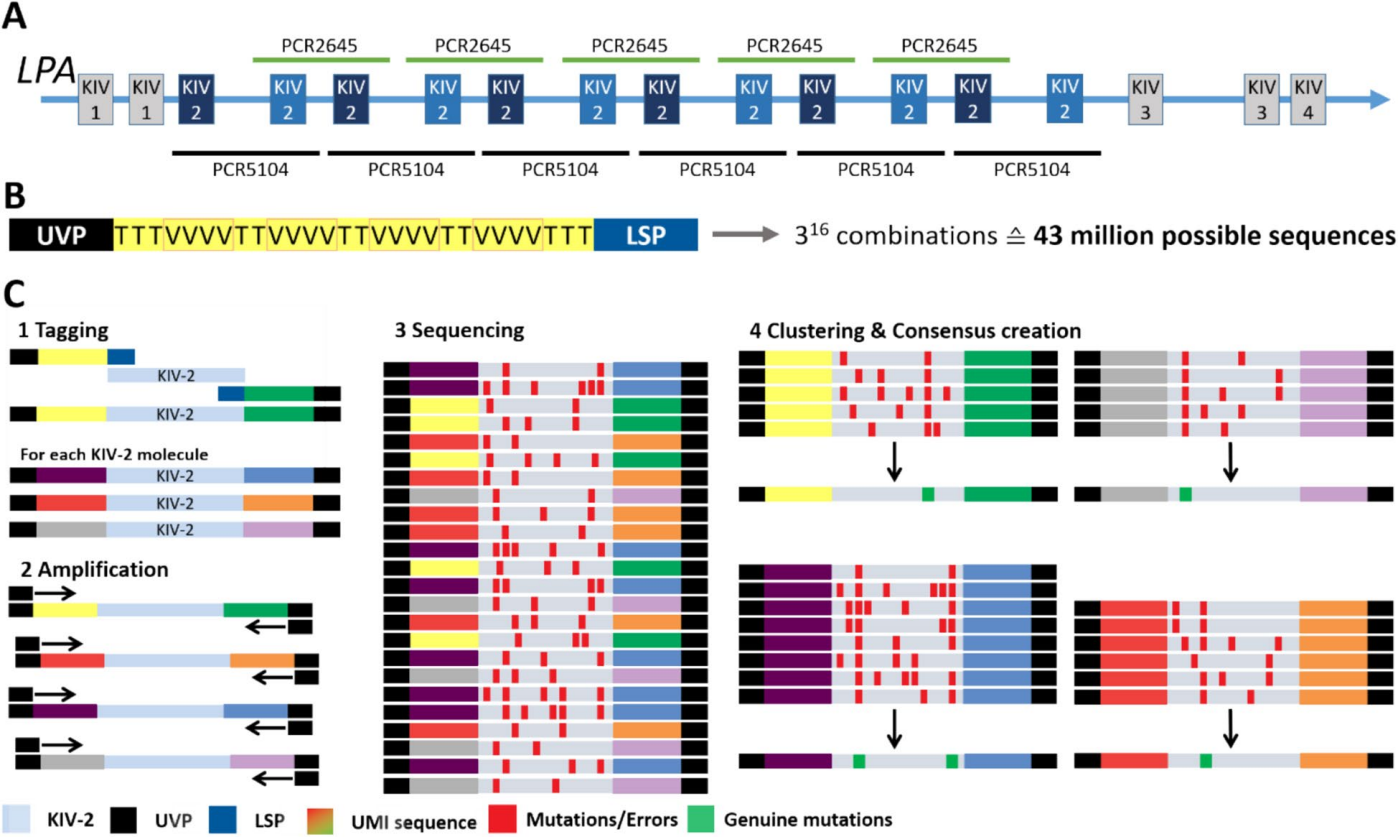
Technical aspects: *LPA* structure, amplicon location, and UMI design. **A** Partial *LPA* gene structure (second exon of KIV-4, KIV-5 to KIV-10 and the C-terminal protease domain omitted) and amplicon location. **B** UMI-ONT-Seq primer design, including a universal primer (UVP) binding site for amplification of the tagged KIV-2 repeats, the UMI sequence, and a locus-specific primer sequence (LSP) (e.g., KIV-2 specific). The UMI design leads to about 43 million possible unique tagging sequences. **C** The LSP primer site is used to tag specifically each KIV-2 repeat in a sample with a unique UMI sequence (1). Subsequent amplification with a universal primer (UVP) (2) and sequencing (3) causes random errors that cannot be differentiated from genuine low-level variants (red boxes in 3). The UMI sequences are used to cluster all sequences originating from one KIV-2 repeat unit and create molecule-wise consensus sequences. This removes errors that occur only in subset of sequences in each UMI cluster, but retains genuine variants that occur in a majority of the sequences in within each UMI cluster

Coupling of ONT-Seq with unique molecular identifiers (UMI-ONT-Seq) allows lowering the ONT-Seq error rate considerably [33, 34]. UMIs are oligonucleotide libraries that randomly tag each template molecule with a unique identifier (Fig. 1). The tagged library is amplified by PCR to generate multiple copies of each UMI-tagged template molecule and full-length sequenced [34]. The reads are clustered according to terminal UMI combination, which reflects their original template, and a consensus sequence is generated for each UMI cluster. This removes PCR and sequencing errors [34] (Fig. 1), while preserving the complete SNP haplotype of each input molecule. In highly repetitive and homologous regions such as the *LPA* KIV-2 repeats, this finally provides highly accurate consensus sequences of each repeat unit.

We describe a comprehensive assessment of UMI-ONT-Seq for SNP detection, SNP haplotyping, generation of consensus sequences for each VNTR unit, and copy number determination by coverage-corrected quantification of the unique haplotypes, using the *LPA* KIV-2 VNTR region as an example for a medically highly relevant complex VNTR region. We created a scalable freely available UMI-ONT-Seq Nextflow analysis pipeline that can be generalized to also any other UMI-ONT-Seq experiment (https://github.com/genepi/umi-pipeline-nf) and demonstrate *LPA* KIV-2 haplotyping by UMI-ONT-Seq in 48 multi-ancestry samples from the 1000 Genomes [35] (1000G) Project.

## Methods

### KIV-2 amplicon naming and reference sequence

All KIV-2 VNTR units were amplified using two amplicons that amplify all KIV-2 units as an overlapping amplicon mixture [24, 25, 30] (Fig. 1A). Any variants that are present only in a subset of KIV-2 units are seen in the corresponding fraction of amplicon molecules, respectively sequencing reads. The “variant level” corresponds to the fraction of reads presenting a certain variant. This resembles sequencing of somatic variation and corresponds to the *LPA* “batch sequencing” concept described by Rosby et al. [36] and adapted to NGS recently [24, 25]. The PCR5104 amplicon spans one KIV-2 unit and portions of the inter-kringle intron. The PCR2645 amplicon spans one inter-KIV-2 intron with the flanking exons and parts of the intra-kringle intron. All positions and fragment lengths in this manuscript are based on the reference sequences for a single KIV-2 unit used in [24].

### Recombinant standards

Generation of the recombinant KIV-2A and KIV-2B standards has been described before [24, 25]. The PCR5104 plasmids differ by 87 positions from each other (including four insertions/deletions [indels]; excluding a microsatellite region in the intra-KIV-2 intron of LPA5104) [24]. The PCR2645 plasmids differ at 120 positions to each other. The plasmids also differ from the respective reference sequences at 28 (PCR5104 KIV-2A), 85 (PCR5104 KIV-2B), 8 (PCR2645 KIV-2A), and 116 (PCR2645 KIV-2B; 113 being in the first intron) positions. Mimicking the in vivo situation, where the KIV-2B represents always the minor component in the KIV-2 VNTR [24, 30], we generated five mixtures of KIV-2B plasmids in KIV-2A background, ranging from 5 to 0.5% KIV-2B. All variants present on the same plasmid represent one haplotype and should be thus detected at the same fractional representation in the reads (i.e., the same variant level) (Additional file 1: Fig. S1). Mixture accuracy was validated by ddPCR (Biorad QX200) (Additional file 1: Table S1).

### Human samples and KIV-2 ddPCR

Sixteen unrelated samples from the healthy-working population SAPHIR (Salzburg Atherosclerosis Prevention Program in subjects at High Individual Risk; sample codes EUR in figures and tables) [37] with KIV-2 SNP data from ultra-deep NGS from Coassin and Schönherr et al. [24] were used to evaluate UMI-ONT-Seq performance in human samples using amplicon PCR5104. The samples were selected to provide a diverse number of KIV-2 repeats per allele and sample (based on Western blot [24] and ddPCR), with an enrichment of carriers of extreme number KIV-2 repeats and medically relevant mutations (e.g., 4925G > A [25] and 4733G > A [26]; Additional file 1: Fig. S2). The genomic KIV-2 repeat number was quantified by ddPCR (Additional file 1: Supplementary Methods and Table S2). Mean confidence interval (CI) width of ddPCR KIV-2 quantification was as low as 4.81 ± 2.34 KIV-2 copies. One sample was excluded due to technical failure.

Three trios (9 samples) and 48 unrelated multi-ancestry samples from the 1000 Genomes [35] (1000G) Project (Yoruba [YRI], Dai Chinese [CDX], Japanese [JPT], Punjabi [PJL]; 12 samples per group) were obtained from the Coriell NHGRI sample repository. They were selected from the following DNA panels of the NHGRI Sample Repository for Human Genetic Research at the Coriell Institute for Medical Research: MGP00009, MGP00012, MGP00013, MGP00020.

The 1000G samples were selected to be well separated based on the probabilistic ancestry maps presented by Gaspar and Breen [38], which are better suited to separate populations within the same continental superpopulation of 1000G than the commonly used principal component analysis clustering [38]. We selected one population per African (AFR), South Asian (SAS), and East Asian (EAS) superpopulation. To explore how UMI-ONT-Seq and especially the haplotype-based copy number calling behaves between distinct but related groups, we added a Japanese population to the already present Chinese CDX group, as Japanese and Chinese populations show subtle yet significant genetic differences [38–40]. We opted to exclude the admixed AMR superpopulations for this validation experiment, as they are highly heterogeneous, including European, East Asian, and African genetic components, depending on the specific sub-population.

### KIV-2 UMI-ONT-Seq principle

UMI-ONT-Seq follows the approach developed by Karst et al. [34] and uses the oligo design from ONT technical note CPU_9107_v109_revC_09Oct2020 (generating 3^16^ diverse oligos; Fig. 1B). The UMI primers consist of a 3′ locus-specific primer (LSP) [24], the UMI, and a 5′ universal priming site (UVP; Fig. 1B; Additional file 1: Table S3). Experimental conditions are given in Additional file 1: Table S4. The LSP parts correspond to the primers 422L and 421U_2 (PCR5104) and 422U and 421L (PCR2645) for *LPA* batch sequencing described by Noureen et al. [30]. The primers bind at conserved locations in the inter-kringle (PCR5104) and intra-kringle (PCR2645) introns (see Fig. 3 in [30]). By tagging the batch sequencing primer with UMIs, each UMI combination tags uniquely a single KIV-2 unit within the KIV-2 wide amplicon mixture produced by the primers.

All PCRs were performed with the ThermoFisher Platinum SuperFi II ultra-high fidelity polymerase (accuracy > 100-fold over Taq [41, 42]). About 2 ng gDNA (i.e., 50,000 KIV-2 template copies under assumption of maximum 80 KIV-2 genomic repeats; observation from our database with > 13,000 apo(a) Western blots [43] and from [44–46]) was tagged with two UMI-PCR cycles (Fig. 1C1) followed by New England Biolabs ExoCIP treatment. The tagged templates were then amplified in two rounds of PCR with UVP primers (early and late UVP-PCR) to produce multiple copies of each tagged molecule (Fig. 1C2). A 0.9 × SPRI beads purification between the two rounds of UVP-PCR was used to reduce background (Additional file 1: Supplementary Methods). After full-length nanopore sequencing (Fig. 1C3), reads are clustered based on the terminal UMI pairs and a consensus sequence is created for every UMI cluster with a defined minimal read number (UMI cluster size; Fig. 1C4). Each consensus sequence represents the sequence of a single genomic KIV-2 VNTR repeat unit.

### Sequencing and basecalling

All samples were full-length sequenced on a MinION 1B system (ONT, Oxford, UK) with either the earlier R9 chemistry (SQK-LSK109 with native barcoding kits NBD-104 and NBD-114 and R9.4.1/MIN106D flow cells) or the newer V14 chemistry (SQK-NBD114 and R10.4.1/MIN114 flow cells) [47]. About 100,000 on-target, full-length reads were generated per sample (Additional file 1: Tables S5 to S9). This was sufficient to recall all expected variants in preliminary experiments with plasmid standards (both unmixed plasmids and mixtures at 1% and 5%) and two human samples. Basecalling was done using guppy v6.3.8 with either “high accuracy” (HAC; for R9 and V14) or “super accuracy” (SUP; for V14) settings. The 15 human PCR5104 SUP samples were additionally duplex basecalled (SUPDUP; for V14) using ONT duplex tools v0.2.20 [48]. The 1000G dataset was basecalled with dorado basecalling model v4.3 [49] and SUP algorithm.

### UMI-ONT-Seq Nextflow analysis pipeline

Initially, our analysis pipeline corresponded to the proof-of-principle UMI analysis pipeline published online by ONT [50] (which follows the pipeline steps of Karst et al. [34]), but was migrated to the Nextflow [51] framework with several optimizations to improve performance and parallelization. The pipeline steps are described in Additional file 1: Fig. S3 and in the Supplementary Methods. In brief, all reads of each barcode are merged, filtered for length (> 1000 bp) and mean per-base quality (> Q9), and aligned to the target reference sequence. Only primary alignments with more than 95% overlap are retained to remove chimeric amplicons. The reads are clustered according to the terminal UMI sequences using VSEARCH [52] and only UMI clusters with ≥ 20 reads were retained for consensus sequence generation (as recommended by Karst et al. [34]). Each cluster is then polished using Medaka v1.7.0 [53]. The UMI extraction, clustering, and reference alignment steps are repeated for the polished consensus sequences to generate the final consensus sequences (clustering step 2).

Extensive analysis of the migrated pipeline revealed inaccurate clustering by VSEARCH (see “Results” section for details). In both clustering steps, VSEARCH clustered distant UMI combinations and separated identical UMI combination into different clusters. We therefore modified the clustering strategy of the pipeline. Looser clustering parameters (80% sequence identity) in clustering step 1 prevent separation of identical UMI sequences. To prevent mixing of distant UMIs into one cluster, all clusters containing more than 12 (R9 HAC), 10 (V14 HAC), and 8 (V14 SUP) reads were then split by taking the first UMI sequence of the cluster file and clustering it with all remaining UMI sequences in the same file that show ≤ 2 bp edit distance (UMI collision probability < 0.01%). The remaining UMIs were treated as a new cluster and clustered iteratively. The minimal UMI cluster size required for consensus creation was derived based on plateauing of the consensus quality at these values (see “Results”). Subsequently, in clustering step 2 stringent clustering parameters (> 99% sequence identity) were used to remove PCR duplicates without mixing distinct UMI clusters.

Our analysis pipeline and test data is available at https://github.com/genepi/umi-pipeline-nf/tree/v0.2.1 under GNU General Public License v3.0. For reproducibility, we provide also the migrated ONT pipeline at https://github.com/genepi/umi-pipeline-nf/tree/ONT_default_clustering_strategy.

### UMI-ONT-Seq residual error rate estimation

Sequencing the unmixed plasmids provides an intuitive way to estimate the residual error rate of the UMI-ONT-Seq as any variation in the consensus sequences can be considered an error. The error rate can be quantified by either averaging the Phred (Q-) scores of each consensus sequence (“consensus sequence Q-score”) and or dataset-wide (“dataset Q-score”). The latter was introduced because the fragment length limits the maximum achievable consensus-sequence Q-score and because perfect consensus sequences produce infinite Q-score. The dataset Q-score was defined as *Error rate* = *n*_*differences*_/*n*_*sequences*_ × *length*_*sequences*_ with the Q-score being *Q* =  − 10 × log_10_(*error rate*) [54].

### Variant calling

Variants in the UMI consensus sequences were called using a modified version of Mutserve [55] v2.0.0-rc13 [56] that does not require bidirectional confirmation. The minimum KIV-2 variant level that needs to be detected is 1.25% (1 in ≈80 KIV-2 units) [25]. To allow for random fluctuations and sequence-context effects, variants > 0.85% variant level were considered true. Variant KIV-2 calling without UMIs was done by omitting the UMI clustering. This resembles ultra-deep KIV-2 NGS sequencing as done in [24, 25]. All reads were aligned to the reference sequence for one repeat unit and conventional low-level variant calling was performed using default Mutserve v2.0.0-rc15 [56] and ClairS-TO v0.0.2 [57]. Extraction of the intronic KIV-2 STR sequences and variants for each KIV-2 unit is described in Additional file 1: Supplementary Methods.

### Variant truth set

Ultra-deep targeted KIV-2 NGS data obtained previously for all SAPHIR samples [24, 25] was used as truth set for UMI-ONT-Seq evaluation. The 1000G variant truth set was generated using a KIV-2 NGS variant calling pipeline on the 1000G 30X whole-genome (WGS) sequencing data [58] (with minor adaptions for WGS data). All UMI-ONT-Seq datasets were benchmarked in terms of sensitivity (true positive rate), specificity (true negative rate), precision (positive predictive value, i.e., the proportion of genuine variants among all variants found), and F1 score (harmonic mean of sensitivity and precision). For the polymorphic intronic short tandem repeat (STR) in PCR5104 (position 2472–2506), no NGS reference data was available and it thus was analyzed separately.

### KIV-2 units haplotype extraction

Haplotypes were extracted using a three-step algorithm (available at https://github.com/AmstlerStephan/haplotyping-KIV2-nf/tree/v0.1.0). (1) Extraction of uniquely occurring haplotypes including all positions that were called as variants in any of the samples (unique haplotypes; see Additional file 1: Fig. S4). (2) Noise polishing and removal of “unlikely” haplotypes (noise-filtered haplotypes). (3) Estimating the repeat number per haplotype by coverage correction (coverage-corrected haplotypes).

The unique haplotypes were determined by extracting from the consensus sequences of each sample the base present at any polymorphic position in the dataset, as found by mutserve variant calling. At positions per sample with a variant frequency < 0.85%, only the major variant was used in the haplotype. Next, only the uniquely occurring haplotypes per sample were retained, including their occurrence in the consensus sequences (unique haplotypes).

To obtain the noise-filtered haplotypes, the residual noise was reduced by clustering identical haplotypes and assigning each haplotype cluster below the threshold *n*_*sequences*_ × 0.0085 to the haplotype cluster with the smallest edit distance, but not more than a maximum edit distance of 1. Next, assuming unbiased and random tagging of KIV-2 repeats in our samples and unbiased PCR amplification, we applied a binomial distribution to determine the minimal occurrence required for a haplotype to be considered genuine. The binomial distribution formula in this context is expressed as:

$$P\left(X=k\right)=\left(\genfrac{}{}{0pt}{}{n}{k}\right){p}^{k}{\left(1-p\right)}^{n-k}$$

Here, *n* represents the total number of haplotypes observed after the UMI sequencing, *p* is the probability of a haplotype to occur, and *k* the minimal occurrence threshold for a haplotype to be considered genuine. The probability is set to 0 (stringent filtering) to calculate the sample-specific minimal occurrence threshold (*k*) for any given haplotype. Haplotypes falling below the determined minimal occurrence threshold *k* have 0 probability to be genuine (being, e.g., generated by the residual noise in the UMI sequencing) and were therefore excluded from the analysis.

To correct the remaining filtered-haplotypes for identical KIV-2 repeats, we calculate the median occurrence of all haplotypes within the consensus sequences, and then divided the occurrence of each haplotype by the median and rounded that to the next integer (coverage-corrected haplotype occurrence). This is done separately for each individual. The sum of the coverage-corrected haplotype occurrence across all haplotypes gives the coverage-corrected number of KIV-2 repeats.

### Data processing

Data processing and visualization was done in R 4.3.1 [59] with ggplot2 v3.3.6 [60] or in bash scripts (available at https://github.com/AmstlerStephan/UMI-ONT-Seq_Analysis). Reading and manipulation of BAM and FASTQ/A files was done using BioStrings [61] v2.68.1. *R*-squared values were calculated using the linear model (lm) function. Bland–Altman plots were generated using BlandAltmanLeh v0.3.1 [62]. The coverage-corrected haplotypes were used to generate unrooted KIV-2 phylograms using MAFFT v7.520 [63] with global iterative refinement (G-INS-i) and modified UPGMA guide trees. Visualization was done with ggtree v.3.8.2 [64]. R package ggvenn v0.1.10 [65] was used to create Venn diagrams directly from the haplotype sequence.

## Results

We performed a comprehensive evaluation of the performance of UMI-ONT-Seq for variant calling, haplotyping, generation of consensus sequences for each VNTR unit, and VNTR copy number determination in the complex *LPA* KIV-2 VNTR [15]. We generated 28 sequencing libraries encompassing both PCR products of two unmixed plasmid standards differing by 87 (PCR5104) and 120 (PCR2645) bases, five plasmid mixtures (ddPCR-validated, Additional file 1: Table S1), and two sequencing chemistries (R9, V14). Moreover, the KIV-2-spanning PCR5104 amplicon was sequenced on 15 human validation gDNA samples and finally used to call mutations, map haplotypes, and quantify the genomic KIV-2 units in 48 1000G samples from 4 different populations.

### UMI-ONT-Seq recapitulates expected variant levels and KIV-2B haplotype fractions in plasmid mixtures

**Fig. 2:**
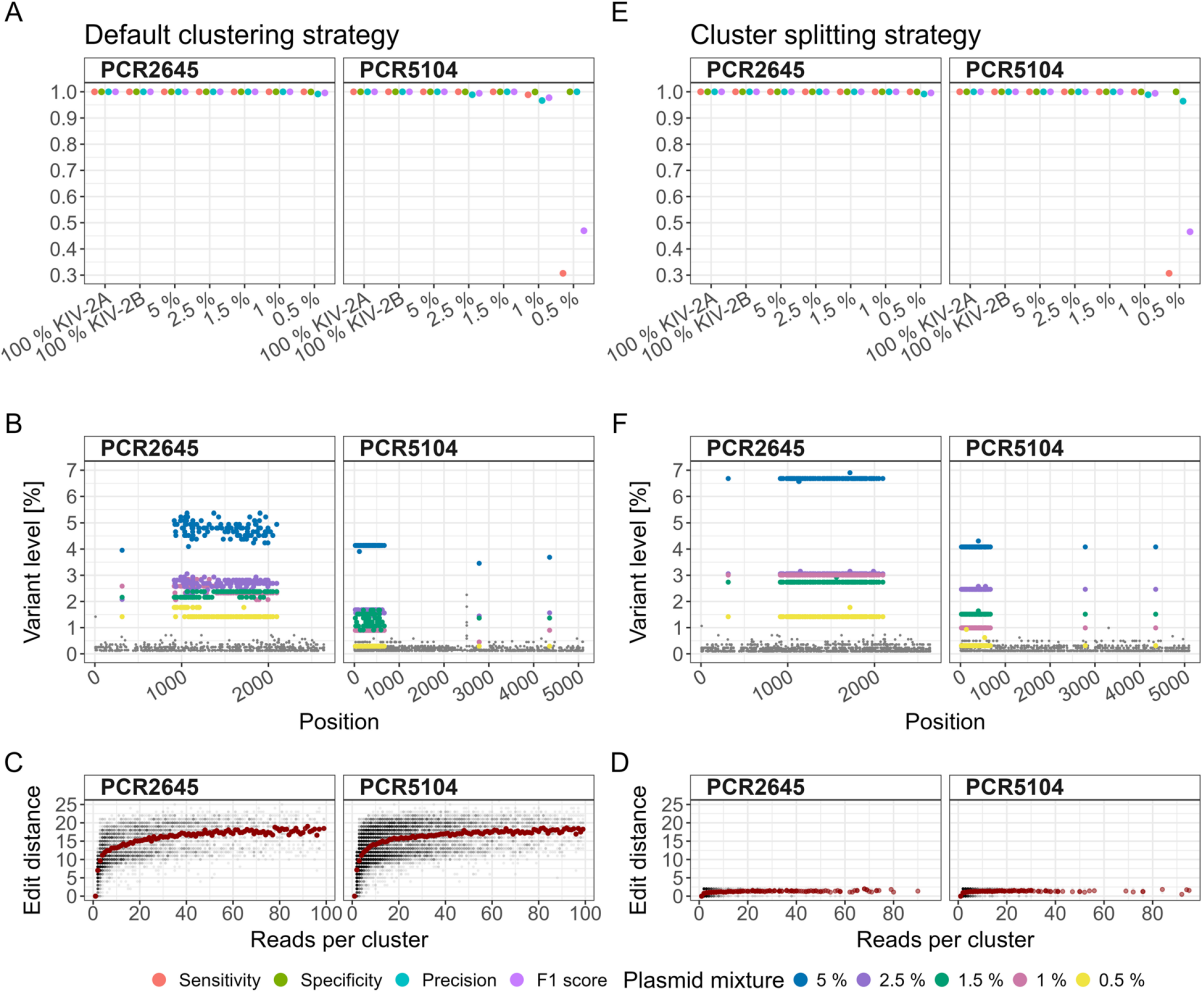
Variant detection in plasmid mixtures with the ONT UMI analysis pipeline using the default clustering strategy and the cluster splitting strategy for the V14 chemistry and HAC basecalling. **A** to **C** show the results for the default clustering strategy; **D** to **F** show the corresponding results for cluster-splitting strategy. **A** and **E** Performance measures for the default clustering strategy in recalling variants of the two unmixed plasmids and plasmid mixtures from 5 to 0.5% KIV-2B in KIV-2A background of two fragments (PCR2645, PCR5104). **B** and **F** Variant levels for the plasmid mixtures from 0.5 to 5% across every position of both fragments. Gray points: low-level residual noise. Variation of detected variant levels of up to ± 1% in absolute values was observed (blue points, 5% mixture). **C** and **D** Edit distance of the UMI sequences for different UMI cluster sizes. Gray: single clusters. Red: average cluster size. Using the default clustering strategy admixing of UMI sequences up to an edit distance of 25 within one cluster (i.e., the sequences did not originate from the same KIV-2 repeat) led to considerable variance in the observed variant levels (**C**). Using the cluster splitting strategy with maximal edit distance 2 between the UMI sequences (**D**) reduced the variant level noise considerably (**E**, colored points) for both fragments and all mixture levels

The switch from R9 to V14 chemistry, as well as basecalling V14 data with SUP or SUPDUP instead of HAC, had, as expected, a large impact on sequencing accuracy (Additional file 1: Table S5). For technical performance values across all experiments see Additional file 1: Tables S6 to S9. Variant calling performance of UMI-ONT-Seq was excellent in all plasmid mixtures down to 1% for both amplicons (Fig. 2A, for R9 data see Additional file 1: Fig. S5A). Notably, no performance difference was seen for V14 data between HAC and the computationally much more expensive SUP basecalling. Specificity was 99.9 to 100% in all samples (Additional file 1: Table S10), but a residual background noise at 0.2 to 0.5% variant level was observed in all plasmid mixtures and sequencing chemistries (Fig. 2B, Additional file 1: Fig. S5B). Therefore, we introduced a cut-off of 0.85% for all further experiments, which includes all bona-fide KIV-2 variants while allowing some technical variation.

Since the variants present on the two plasmids represent distinct haplotypes, we expected all mutations from the same plasmid to occur at the same level. We found that all mutations from the same plasmid were, indeed, close to the expected level on average, but showed considerable per-position noise when analyzed with the ONT UMI analysis pipeline using the default clustering strategy (Fig. 2B). Systematic analysis of the UMI clusters revealed that inaccurate clustering of the UMI sequences by VSEARCH resulted in partially heterogeneous UMI clusters (Fig. 2C, Additional file 1: Table S11). If the UMI clusters contained a mixture of sequences (e.g., KIV-2A and KIV-2B sequences), the cluster-polishing step produced noisy variant levels (Fig. 2B, Additional file 1: Table S12).

Implementation of the cluster splitting strategy reduced the edit distance in the UMI clusters considerably (Fig. 2D). This had no impact on variant calling performance in the V14 chemistry (Fig. 2E, Additional file 1: Table S13) and most importantly, mutations originating from the same plasmid now showed virtually no residual variation and matched the expected values very accurately (Fig. 2F). The number of plasmid standards with no variant level noise increased from 4/14 to 12/14 samples in HAC and SUP. The median variant level noise was reduced by 3.1-fold and 2.3-fold for V14 HAC and SUP (R9 HAC: 0.8-fold, Additional file 1: Table S14). This indicates that our cluster splitting strategy allows accurate recalling of the haplotype of each read (Additional file 1: Table S15). All further results are based on the UMI-ONT-Seq analysis pipeline using the cluster splitting strategy. All plasmid sequencing data is available at reference [47].

### UMI-ONT-Seq produces highly accurate KIV-2 consensus sequences at ≥ 6 reads per UMI cluster

We investigated the relationship between the UMI cluster size and consensus sequence qualities using the unmixed plasmids (KIV-2A and KIV-2B), where it can be assumed that any difference between the consensus sequences represents a PCR or sequencing error.

**Fig. 3:**
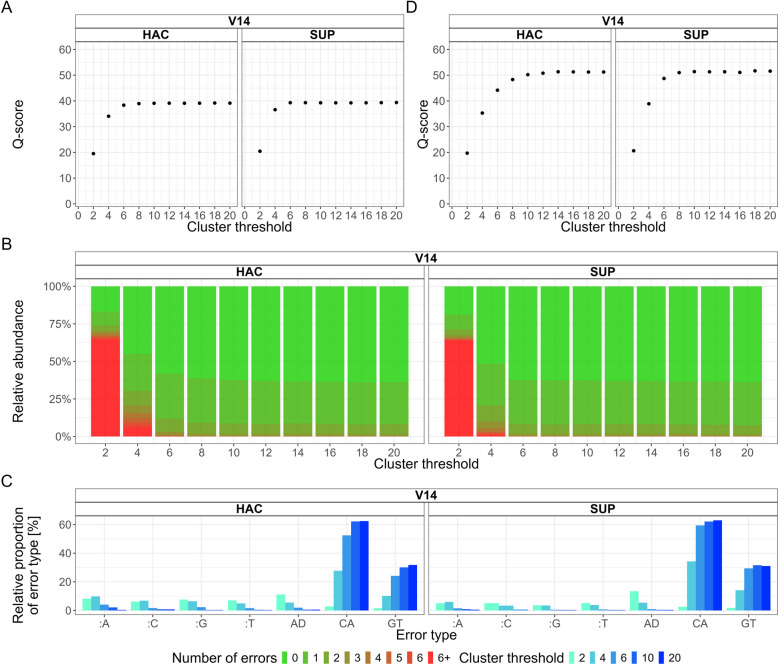
Impact of the minimal cluster size on consensus sequence quality and error-profile. **A** Dependency of the dataset Q-score from the minimal cluster size. V14 HAC and V14 SUP dataset Q-score increases rapidly being close to Q40 already at cluster size 6 to 10. **B** Percentage of perfect reads depending on the minimal cluster size threshold. At Q40 dataset Q-score 62% of all consensus sequences are error-free and 98.5% of all cluster had no more than 2 errors for both V14 conditions. **C** Error type frequency for V14 HAC and SUP. C to A and G to T transversions were the most common errors. “:N” denotes insertions; D denotes deletions. **D** Dataset Q-score at different cluster thresholds after filtering for the sequencing chemistry-specific errors. The dataset Q-score of V14 consensus sequences reaches Q50 already at a cluster size of 6 to 10

Up to 10 reads per UMI cluster, the dataset Q-score increased rapidly in the V14 data, reaching Q40 already at 6–10 reads per cluster (Fig. 3A, Additional file 1: Table S16; 14 reads for R9 data, Additional file 1: Fig. S6A). The consensus sequence Q-score increased in a similar manner as the dataset Q-score, reaching the maximal quality after 6 reads per cluster for the V14 chemistry (14 for R9 HAC; Additional file 1: Fig. S7A). At 6 reads per cluster already 96.8% (HAC) and 98.3% (SUP) of all consensus sequences showed no more than 2 errors, and 58.1% (HAC) and 62.5% (SUP) were even error-free (Fig. 3B; R9: 79.6% and 33.5%; Additional file 1: Table S17 and Fig. S7B).

For high minimal cluster sizes (≥ 10), indels became the most prominent error type for the older R9 chemistry (Additional file 1: Fig. S6B), while both V14 conditions were primarily characterized by C to A and G to T transversions (Fig. 3C). Disregarding these systematic errors, which were all below the 0.85% variant level, further improved the consensus sequence qualities for both V14 kits considerably (Fig. 3D). Both basecalling algorithms again reached dataset Q-score Q40 at 6 reads per cluster and even Q50 at 10 and 8 reads (V14 HAC: 92 errors in 10 Mb; V14 SUP: 94 errors in 11.9 Mb; Additional file 1: Table S19). Already at cluster size 6, 89.9 to 95.5% of all consensus sequences were error-free (Additional file 1: Fig. S7 and Table S16).

### Accurate variant calling of variants within the KIV-2 VNTR in human samples

**Fig. 4:**
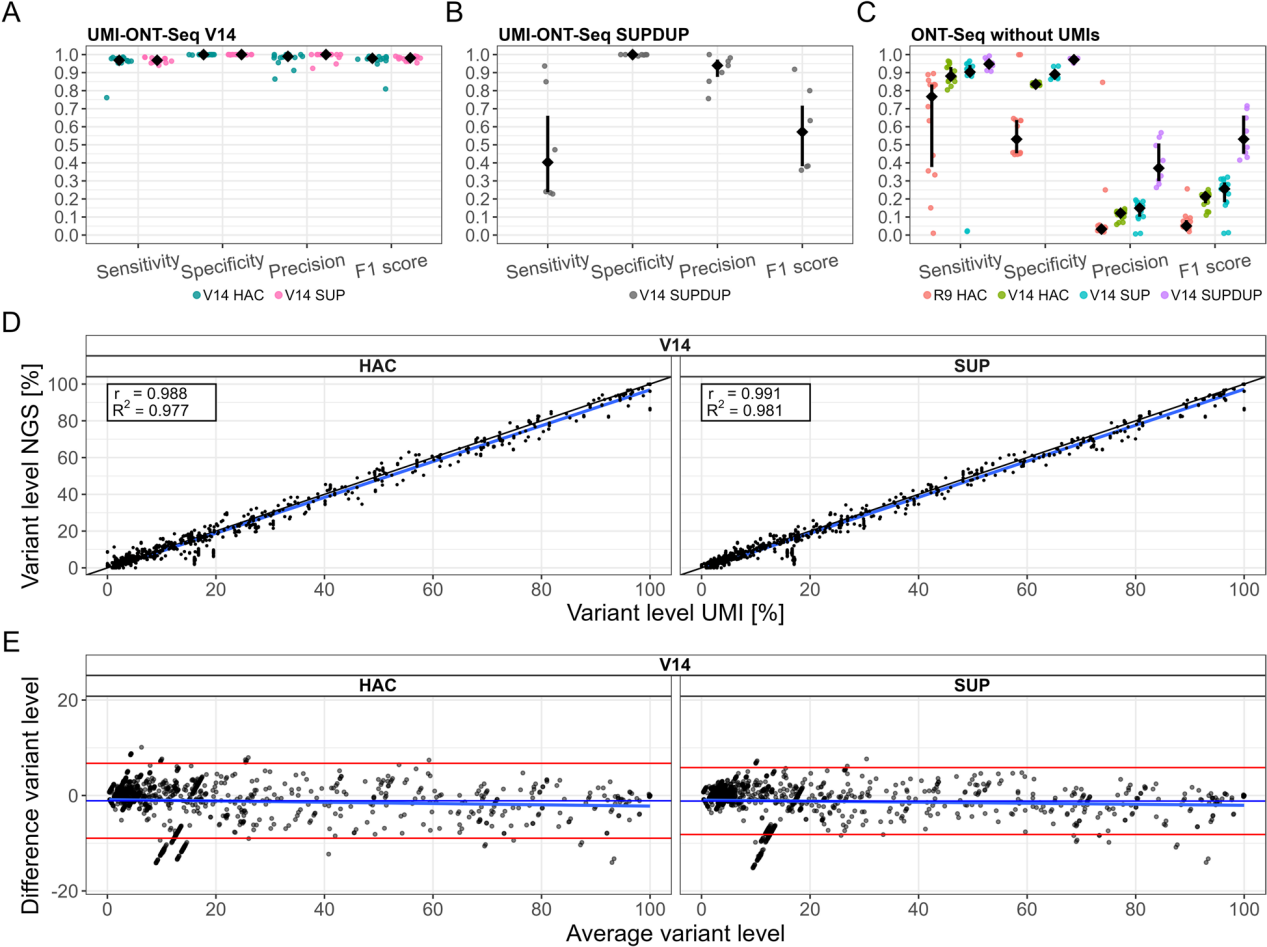
Performance measures compared to ultra-deep NGS sequencing of 15 human DNA samples (SAPHIR) for the V14 chemistry (HAC and SUP basecalling). **A** Performance of UMI-ONT-Seq for the 15 human DNA samples. Black points are median values. Colored points are the single samples. We observed high agreement between the ultra-deep NGS sequencing and UMI-ONT-Seq for most samples, leading to median performance values above 95% and slightly higher performance values for SUP basecalling. **B** Performance values for duplex basecalling (SUPDUP). Black points and lines are median values and interquartile range. Gray points are the single samples. Despite increased raw read quality, there was a significant drop in sensitivity when using SUPDUP basecalling (see “Discussion” for explanation). **C** Performance values for ONT-Seq (without UMIs) for different chemistries (R9, V14) and basecalling algorithms (HAC, SUP, SUPDUP) (black points and lines are median values and interquartile ranges; colored points are the single samples). Sensitivity increased with increasing raw read quality up to median values of 95% for SUPDUP basecalling, but precision and F1 score were consistently low due to by high number of false positives. **D** Correlation of variant levels for each mutation of all 15 DNA samples (black points) of UMI-ONT-Seq for V14 HAC and SUP basecalling compared to ultra-deep NGS sequencing. We observed nearly 100% correlation (*r* and *R*.^2^ > 0.977) for both conditions, with no bias across the variant level range (**E**)

We evaluated the performance of UMI-ONT-Seq on the KIV-2 PCR5104 fragment, which encompasses about 92% of the KIV-2 VNTR region, in 15 human validation samples with KIV-2 batch NGS sequencing data available [24]. Sensitivity, specificity, precision, and F1 score were mostly very close or equal to 100% (Fig. 4A; see Additional file 1: Table S20 for single sample values and Additional file 1: Fig. S8 for R9 HAC). Most importantly, specificity (mean ± SD) was 1.0 ± 0.001 for all conditions, demonstrating a very low false-positive rate of UMI-ONT-Seq despite its relatively high raw-read error rate (Additional file 1: Table S21). Also in human samples V14 data performed generally better than R9, while V14 SUP provided marginal benefit over V14 HAC (Fig. 4A). Importantly, despite providing considerably higher raw read accuracy (median read quality ≈Q28; Additional file 1: Table S5), UMI-ONT-Seq with duplex basecalling (SUPDUP) leads to very low sensitivity and F1 score (0.481 ± 0.298; 0.578 ± 0.221; Fig. 4B; Additional file 1: Table S21). This was actually expected due to the specific technical implementation of duplex basecalling and is addressed in the “Discussion” section.

To evaluate the advantage of the UMI-ONT-Seq, we called the KIV-2 variants on the same ONT-Seq data without UMI clustering (simulating a conventional KIV-2 deep sequencing approach [24]). The performance of the variant calling without UMIs increased continuously from R9 HAC to V14 HAC to V14 SUP to V14 SUPDUP (Fig. 4C, Additional file 1: Table S22), but precision and F1 score were considerably below UMI-ONT-Seq in all conditions. For V14 SUPDUP without UMIs, sensitivity and specificity reached 0.950 ± 0.031 and 0.971 ± 0.008, but precision and F1 score were only 0.399 ± 0.122 and 0.554 ± 0.123. For V14 SUP without the UMIs, 9644 unique variants were detected, of which 1695 were confirmed by UMI-ONT-Seq, while 7949 variants were filtered as amplification/sequencing errors (mean ± SD; 529.93 ± 142.19 filtered variants per sample). UMI-ONT-Seq also considerably exceeded the performance of the nanopore-specific low-level variant caller ClairS-TO (Additional file 1: Fig. S9).

Importantly, UMI-ONT-Seq not only discriminated variants in the KIV-2 very efficiently, but also recapitulated very closely the individual variant levels measured previously by deep NGS [24] (*R*^2^: 0.977 and 0.981 for V14 HAC and SUP, Fig. 4D, Additional file 1: Fig. S10). With V14 conditions, no bias was observed across the whole variant level range (Fig. 4E; Additional file 1: Fig. S11 for R9). Conversely, both the R9 chemistry and especially the UMI-free conditions showed noticeable bias and less correlation, which was exacerbated by a high number of false-negatives in the UMI-free analysis (Additional file 1: Figs. S10 and S11).

### UMI-ONT-Seq preserves the haplotype of each KIV-2 repeat unit and allows precise KIV-2 copy number quantification

We used 15 human DNA samples and the two unmixed plasmids of PCR5104 to develop an algorithm (for V14 SUP) to estimate the KIV-2 copy number in human genomic DNA. The number of unique haplotypes showed a high correlation with the KIV-2 copy number measured by ddPCR (*r* = 0.84, *R*^2^ = 0.709, Additional file 1: Fig. S12A and B), but overestimated the number of repeats compared to ddPCR (mean ± SD, 9.1 ± 8.2, Additional file 1: Table S23). Merging unlikely haplotypes (see “Methods”) reduced the deviation to only − 3.6 ± 3.9 repeats (Additional file 1: Table S23) and increased the correlation with ddPCR considerably (*r* = 0.96, *R*^2^ = 0.92, Additional file 1: Fig. S12C), but in turn led to an underestimation of the KIV-2 copy number, especially for the high KIV-2 numbers (Additional file 1: Fig. S12D).

**Fig. 5:**
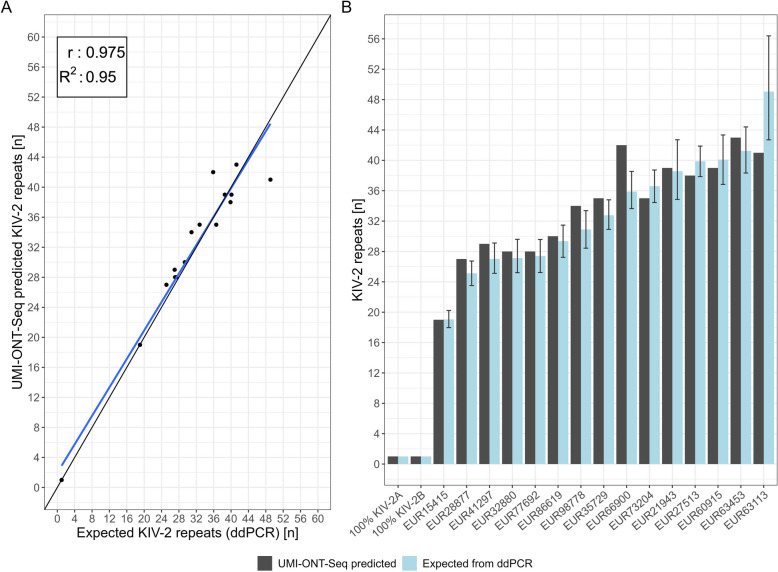
Correlation between the expected and UMI-ONT-Seq predicted number of KIV-2 repeat units. **A** Correlation of the coverage-corrected UMI-ONT-Seq haplotypes with the number of KIV-2 repeats expected from ddPCR. Coverage-corrected UMI-ONT-Seq haplotypes show high correlation with KIV-2 number determined by ddPCR. **B** The barplots report the predicted versus measured number of KIV-2 repeats per sample including the confidence interval of the ddPCR quantification. Seventy-five to 80% of the samples are within the 95% confidence interval of the ddPCR. EUR: European samples from Austria (SAPHIR study)

We thus hypothesized that truly identical KIV-2 repeats may occur more often than assumed and may bias the KIV-2 copy number estimation (Additional file 1: Fig. S13). Indeed, in line with this hypothesis, coverage correction of the occurrence of each haplotype finally led to nearly perfect correlation between the predicted and the expected KIV-2 copy number (*r*: 0.975, *R*^2^: 0.95) and reduced the mean deviation to only 0.4 ± 2.9 repeats (Fig. 5A, Additional file 1: Table S23). Both unmixed plasmids showed only one single haplotype and for 12 of 15 samples the estimated KIV-2 copy numbers were even within the narrow confidence interval (CI) of ddPCR (Fig. 5B).

Finally, we applied UMI-ONT-Seq to three different trios from the available 1000G sample set [47] (2 YRI and 1 CHS families; Additional file 1: Table S24 for an overview per sample). The number of KIV-2 repeats determined by UMI-ONT-Seq accurately reflected the KIV-2 copy number determined by ddPCR (*r*: 0.974; *R*^2^: 0.948; 6 of 9 samples within the CI of the ddPCR; Additional file 1: Fig. S14). Strikingly, all haplotypes that were found in the children were also found in the parents, without any haplotypes private to the children (Additional file 1: Fig. S14).

### KIV-2 repeat number quantification and haplotype diversity in 48 samples from 1000G

Despite the Lp(a) trait shows marked differences across ancestries, genetic Lp(a) research in the last decades was focused mainly on individuals of European ancestry. Little is known about genetic variability in non-European samples, especially within the KIV-2 region. To provide a first impression of the diversity of the KIV-2 VNTR across ancestries, we performed KIV-2 UMI-ONT-Seq on 48 randomly selected, unrelated gDNA samples from four non-European 1000G populations (Yoruba [YRI], Dai Chinese [CDX], Japanese [JPT], Punjabi [PJL]; 12 samples per group) [47].

In this sample set [47], the results reflect previously suggested differences between ancestries, such as the different frequencies of KIV-2B and KIV-2C units [24, 30]. For example, KIV-2C repeats occurred in 50% of the analyzed East Asian samples (CDX and JPT) but were completely absent in the analyzed African samples (Additional file 1: Table S25).

**Fig. 6:**
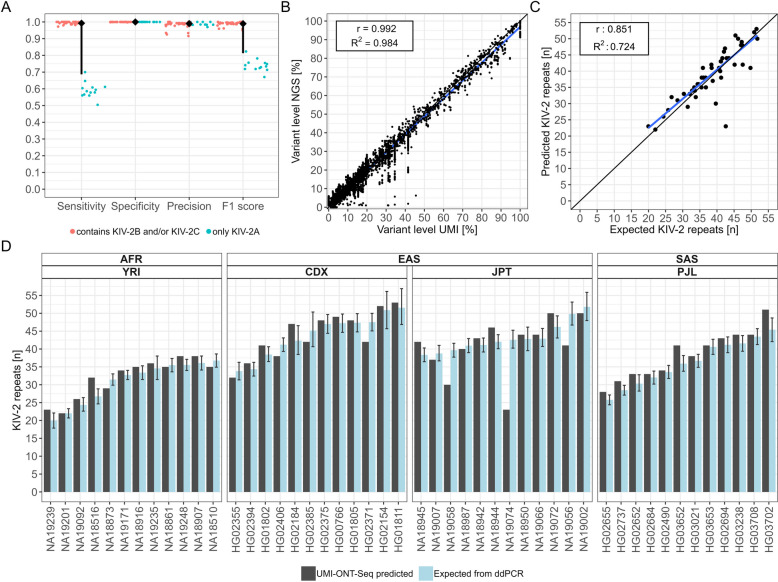
UMI-ONT-Seq analysis of 48 samples from four populations of the 1000 Genomes Project. **A** Performance of UMI-ONT-Seq compared to KIV-2 specific variant calling of high-coverage whole-genome sequencing (WGS [66]) for the 48 1000G samples [66]. While sensitivity and specificity are close to perfect (mean ± SD: 0.993 ± 0.01, 0.996 ± 0.005), precision and respectively F1 score deviate from the WGS data. All mutations that were additionally found by the UMI-ONT-Seq were previously classified as KIV-2B specific, intronic variants, which are reported to be difficult to call in WGS data [24]. **B** Correlation of UMI-ONT-Seq variant levels versus WGS variant levels (*n* = 5968 variants). Found variant levels of both methods are highly correlated (*r*=0.992, *R*^2^ = 0.983). Deviations were observed only for KIV-2B specific variants. **C** Correlation of ddPCR measured versus UMI-ONT-Seq predicted number of KIV-2. We observed a high correlation between UMI-ONT-Seq quantified and the number of KIV-2 repeats determined by ddPCR (*r*= 0.851, *R*^2^ = 0.724). **D** Comparison of UMI-ONT-Seq with ddPCR quantification. UMI-ONT-Seq accurately predicts the number of KIV-2 repeats. The mean (± SD) deviation between UMI-ONT-Seq and ddPCR was − 0.295 ± 4.26 repeats. For 32 of 48 samples even within 95% confidence interval of ddPCR

For the 35 samples that contain KIV-2B and KIV-2C repeats, variant calls from UMI-ONT-Seq and NGS closely agreed (mean ± SD; sensitivity: 99.5 ± 0.7%, specificity: 100 ± 0.1%, precision: 98.3 ± 2.2%, F1 score: 98.9 ± 1.3%; Fig. 6A; Additional file 1: Tables S26 and S27 for per-sample values). Specificity and precision were equally high also for the 13 samples containing only KIV-2A repeat units, but sensitivity was considerably lower in these samples (59.2 ± 4.7%, Fig. 6A and Additional file 1: Table S26). This is caused by known issues of NGS-based variant calling in this complex region [66]. The non-repetitive KIV-3 apo(a) domain presents the same exonic sequence as KIV-2B units within the KIV-2 VNTR. When using WGS data from samples that do not contain KIV-2B units, reads from the KIV-3 domain are mistakenly aligned to KIV-2, resembling KIV-2B-specific variants and thus causing false-positive variant calls [66]. The KIV-2 specific amplification in UMI-ONT-Seq alleviates the wrong alignments. Exclusion of KIV-2B specific variants increased sensitivity for all 48 samples to 98.7 ± 1.8% (Additional file 1: Table S28). As for the SAPHIR validation dataset, NGS and UMI-ONT-Seq variant levels were highly correlated also in 1000G dataset (*r* = 0.992, *R*^2^ = 0.983, Fig. 6B). We found no systematic bias and only deviations in the aforementioned KIV-2B specific mutations. Remarkably, KIV-2B specific mutations were detected at the same variant level, as expected from one haplotype (Additional file 1: Fig. S15 and see Additional file 3 for variant calling results for all 48 1000G samples). When comparing the mutation density of the KIV-2 VNTR with the other KIV units, we found a higher normalized mutation density for the KIV-2 VNTR (median mutation density of the other KIV units = 0.0004–0.0015; KIV-2 VNTR = 0.004 [IQR: 0.0034–0.0047]; Additional file 1: Fig. S16A).

We observed a similar step-wise improvement for the KIV-2 repeat number estimations between the different quantification strategies as in the SAPHIR validation set (Additional file 1: Table S29 and Fig. S17). Correlation with ddPCR data for the 48 1000G samples was *R*^2^ = 0.724, which raised to *R*^2^ = 0.903 after exclusion of two outliers (Fig. 6C). The mean difference was as low as 0.295 ± 4.259 repeats and 32 of 45 samples were even within the narrow CI of the ddPCR (Additional file 1: Table S29 and Fig. 6D).

In summary, UMI-ONT-Seq provides a new, very accurate, ancestry-independent method for variant calling, haplotype extraction, and determination of KIV-2 repeat number for large as well as short alleles, which well exceeds the technical limit of the commonly used KIV-2 VNTR qPCR method [67–70]. Albeit of limited sample size, this dataset may support generation of new hypotheses addressing the Lp(a) differences across ancestries.

### Exploration of the KIV-2 intronic short tandem repeat

Leveraging the high accuracy of UMI-ONT-Seq, we explored the poorly described CA short tandem repeat (STR) in each KIV-2 intron. This STR shows pronounced variability between the KIV-2 units and thus resembles somatic STR variation, which is a further class of hard-to-resolve genetic variation. The KIV-2 STR had been characterized before only once in just 2 individuals [71].

To explore the STR region in the KIV-2 intron, we extracted this region from each of the 62,679 consensus sequences generated by UMI-ONT sequencing across all 63 genomic DNA samples (SAPHIR and 1000G) and the two unmixed plasmids. We found a median of 23 unique STRs per genomic DNA sample (IQR: 20–27). In close agreement with the prior work, we observed STR lengths between 8 and 22 repeats (12–18 in Rosby et al. [71]; Additional file 1: Fig. S18 and Table S30). We observed degeneration of the STR region at mainly positions 7 and 15 from to either GA or AA in 5685 (≈9.1%) UMI consensus sequences (Additional file 1: Table S31). Both the degeneration patterns and the STR diversity showed potential population-specificity (Additional file 1: Tables S31 and S32). This provides a further example of the capabilities and broad applicability of UMI-ONT-Seq to analyze complex genetic variation. Studies with larger sample sets are warranted to provide a comprehensive picture of the population-specific differences.

### Haplotype diversity in 63 multi-ancestry samples

**Fig. 7:**
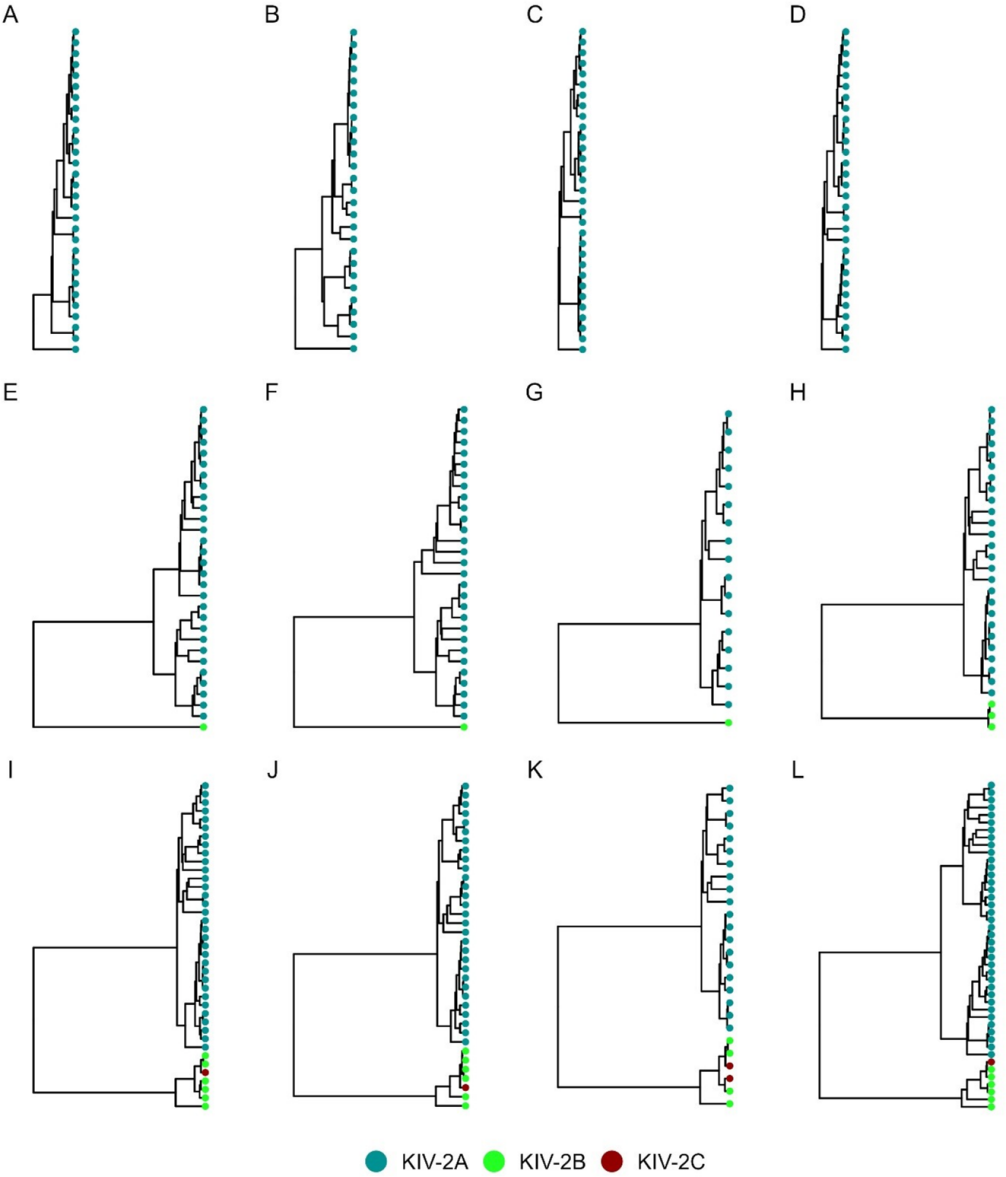
KIV-2 subtype specific haplotype diversity of 12 representative human gDNA samples in the present study (1000G, SAPHIR). Splitting the samples by their containing KIV-2 subtypes revealed 2 major clusters containing either only KIV-2A (**A**–**D**, KIV-2A repeat haplotypes marked in blue) or the phylogenetically distant KIV-2B (**E**–**H**, KIV-2B repeat haplotypes marked in green) and C (**I**–**L**, KIV-2C repeat haplotypes marked in red) subtypes. While the KIV-2B and C cluster contain very similar sequences, with low diversity, the KIV-2A cluster has large internal differentiation (see Additional file 1: Fig. S19 for all samples)

Finally, we performed an exploratory analysis to investigate whether the retrieved haplotypes reflect known KIV-2 clusters and subtypes. We extracted the haplotypes based on variant calling of all 63 genomic DNA samples from 1000G and SAPHIR (see Additional file 2 for a variant calling summary), to analyze the diversity in the KIV-2 repeats. In 2348 KIV-2 repeat units (37.27 ± 7.97 per sample; based on ddPCR), we found 1993 haplotypes (based on the noise-filtered haplotypes) of which 1916 were unique throughout the whole sample set. We were readily able to classify the KIV-2 haplotypes into the commonly known KIV-2A, B, and C subtypes (representative samples in Fig. 7; all samples in Additional file 1: Fig. S19). We observed two novel clusters within KIV-2A, which were defined by three positions (35, 3103, and 4358; Additional file 1: Fig. S20). These had been proposed previously as new haplotypes also in [24]. Interestingly, these positions were invariant in all KIV-2B and C repeats across all 63 samples. Analysis of the KIV-2B and C repeats revealed 5 positions, which define the three clusters of KIV-2B and C (positions 50, 2409, 5037, 5045, and 5052; Additional file 1: Fig. S21). Interestingly, KIV-2C repeats did not build a distinct cluster (Additional file 1: Fig. S22).

Given the unique per-repeat resolution of UMI-ONT-Seq, we finally chose to characterize in detail the haplotypes of two frequent, clinically relevant KIV-2 SNPs (4925G > A [25] and 4733G > A [26]) in the present sample set. Either one of the two SNPs was found in 7 samples (6 EUR, 1 PJL). Both variants were exclusive to KIV-2A repeats. Remarkably, three otherwise unrelated samples showed exactly the same sequence for the complete 5.1 kb long repeat unit carrying the 4733G > A variant and two unrelated samples showed exactly the same sequence of the 4925G > A variant carrying repeat unit (Additional file 1: Fig. S23). The background haplotypes of these variants were clearly located on one of the two different KIV-2A clusters (Additional file 1: Fig. S23). This highlights the potential of UMI-ONT-Seq to preserve highly accurate, full-length, and per-repeat haplotype information of the KIV-2 VNTR and gaining profound insights in the per-unit haplotype structures within this complex VNTR.

## Discussion

Large, highly similar VNTR repeat units are a major constituent of the “dark genome” [2]. Nanopore sequencing has recently achieved well beyond 99% variant calling accuracy for germline mutations [72, 73], but identification and direct phasing of differences in highly homologous regions (especially VNTRs) remains challenging. The medically highly relevant *LPA* KIV-2 VNTR [17] is among the most complex coding VNTRs in the human genome [15, 27] and still poses issues in VNTR variant calling [1, 11, 74], making it a challenging and interesting model. We extensively evaluated the performance of amplicon-based UMI-ONT-Seq for SNP detection, direct SNP phasing, and determination of VNTR repeat numbers.

In all experiments, the UMI-ONT-Seq with V14 chemistry showed nearly perfect variant calling performance, extraordinary correlation with NGS data, and no systematic bias across the variant level range. The effects of raw read quality on variant calling were marginal between V14 HAC and V14 SUP and affected only a few positions in the PCR5104 amplicon (Additional file 1: Table S10). UMI-based variant calling clearly outperformed UMI-free KIV-2 variant calling, which suffered from low precision and low sensitivity, depending on the variant caller used.

The low performance of using duplex called UMI reads despite nearly Q30 raw read quality may appear counterintuitive but is intrinsic to the duplex calling process. Duplex basecalling performs consensus basecalling on all strands that enter the same pore within 20 s, show similar length, and have ≥ 60% homology between the last, respectively first 250 bp. With amplicons showing > 90% similarity like the KIV-2 amplicons [24], this leads to extensive false strand pairings.

Using the V14 chemistry, dataset-quality achieved ≈Q40 already with 6–8 reads per UMI cluster, providing highly accurate sequences of each KIV-2 repeat unit. We identified a V14-specific error profile, which was systematic and limited the consensus quality. Indeed when disregarding these errors, 95.5% of all UMI-ONT-Seq consensus sequences were error-free at cluster size 6 to 8. These characteristic transversion errors might represent a current limitation of UMI-ONT-Seq, especially for very low-level (≤ 0.5%) mutation detection. However, even without error profile correction, one third of the consensus sequences were error-free and 99.7% of the sequences had no more than three errors. The observation that the residual errors are systematic makes them well addressable bioinformatically and/or by modified Medaka training sets.

Overall, the performance of V14 was significantly better than previous reports using R9 chemistry across all experiments. Karst et al. reported that 15 to 25 reads per UMI cluster were required to achieve Q40 dataset error rates with R9 chemistry [34], which is in agreement with our observations (Additional file 1: Fig. S6). Conversely, the low minimal cluster size required by V14 for Q40 quality now puts UMI-ONT-Seq close to the performance of UMI-tagged Pacific Bioscience circular consensus sequencing (PacBio CCS), which requires three reads per UMI cluster for Q40 [34] but comes with 100 times higher equipment investments compared to ONT MinION systems. Of note, PacBio CCS data without UMIs showed chimeras up to 3% variant level, thus requiring UMIs for VNTR sequencing also with PacBio data despite the higher intrinsic quality of PacBio CCS. UMI-ONT-Seq allows multiplexing ≈50 gDNA samples per MinION flow cell at roughly 25 € per sample, while a single PromethION flow cell has sufficient capacity to analyze > 96 samples at < 10 € per sample. This corresponds to ≈0.06/0.025 cents per consensus sequence (assuming roughly 40,000 consensus sequences on average in our setup). This allows sequencing 192 samples on a compact PromethION P2 system every 72 h. UMI-ONT-Seq thus enables decentralized sequencing of the KIV-2 region at scale across ancestries with moderate equipment investment. This may open new avenues to refine variability maps in this complex region and improve reference datasets.

All observations confidently supported that UMI-ONT-Seq provides nearly error-free consensus sequences that allow direct experimental haplotyping of KIV-2 units down to ≤ 1% fractional representation. For some difficult variants with ambiguous results, UMI-ONT-Seq might be even more reliable than NGS. For example, we have previously shown an underrepresentation of KIV-2B variants in ultra-deep NGS [24]. These variants now show considerably higher variant levels in UMI-ONT-Seq (Additional files 2 and 3). In addition to the KIV-2B specific variants, a so far not completely defined subset of variants most likely characterizes several subtypes of KIV-2 repeats and increases the mutation density of the KIV-2 VNTR compared to the other KIV units within our 1000G exploration sample set (Additional file 1: Fig. S16B and C). In larger studies, the UMI-ONT-Seq derived haplotypes per KIV-2 repeat could provide an instrument to define these KIV-2 subtypes. The high consensus sequence quality allowed direct extraction of the repeat structure of an STR located in the intron of the KIV-2 VNTR, which is a challenging task as it resembles somatic STR mosaicism. Given the growing interest in somatic STR mosaicism [75], UMI-ONT-Seq may provide a new efficient way to generate high-quality reference data.

We also employed UMI-ONT-Seq in a limited sample set from the 1000G study to provide first insights into the phylogenetic subclusters and the haplotype context of two frequent disease-relevant SNPs hidden in the KIV-2 VNTR (KIV-2 4925G > A and 4733G > A). We could readily define subcluster-specific mutations and observed high sequence homology (up to 100%) of 4925G > A and 4733G > A carrying repeats (possibly indicating either rather recent mutation events or a conservation of these Lp(a)-reducing haplotypes). UMI-ONT-Seq was readily able to resolve the full-length haplotype of these variants, revealing, in this limited sample set, location on different KIV-2A subclusters. More samples will be needed to investigate these patterns at scale. Given the huge human diversity, our sample set has to be regarded as a hypothesis-generating example of the potential of this technology for analysis of the *LPA* KIV-2 (and possibly similarly complex regions). Larger studies are required to appreciate these complex patterns across ancestries comprehensively.

Given the high per-repeat resolution of UMI-ONT-Seq, we hypothesized that the genomic VNTR repeat number could be accurately determined by coverage-corrected quantification of the unique haplotypes, because truly identical KIV-2 repeats that would arise from, e.g., recent expansions would be detectable by higher normalized coverage. We show in 63 samples from European, African, East Asian, and South Asian populations that UMI-ONT-Seq indeed well exceeds the technical limit of the largely used [76] KIV-2 qPCR assays. It provides repeat number estimates that are comparable to high resolution ddPCR, which is among the most precise technologies for copy number quantification and is able resolve ≈1.1–1.2-fold differences (compared to about twofold in qPCR) [77, 78]. We note that our validation sample set did not include a Hispanic population (admixed Americans, AMR), which are a heterogeneous population [38] with complex genetics. Despite requiring experimental validation, we are confident that UMI-ONT-Seq performs equally well in AMR populations, as it relies solely on the coverage-corrected number of haplotypes, which should not be affected by the ancestry (in contrast to using occurrence of specific “marker” SNPs or haplotypes for copy number estimation).

Of note, a recent preprint by Behera et al. reports an algorithm to determine the KIV-2 copy number using NGS data, which has been implemented in the Illumina DRAGEN analysis suite [11]. The authors determined the KIV-2 copy number of all 1000G samples and were able to phase the copy number to the two *LPA* alleles in 47% of the samples, providing the allelic KIV-2 copy number. The KIV-2 copy number reported by Behera et al. matches accurately our ddPCR data and, more importantly, the KIV-2 copy number derived by UMI-ONT-Seq matches accurately the data of Behera et al. in 46 of 48 samples (*R*^2^ = 0.965 for these 46 samples, Additional file 1: Table S33). Conversely, current NGS-based approaches do not provide full-length sequences and haplotypes of each KIV-2 unit. Another complementary method is ultra-long nanopore sequencing with high-molecular weight (HMW) DNA, which can achieve reads > 100 kb [14, 79]. However, generation of HMW DNA requires laborious procedures [80], while commonly available biobanks used conventional DNA extraction methods. Moreover, an HMW sequencing approach requires WGS, which is very costly at scale and may not provide the coverage level needed to accurately resolve differences between highly similar KIV-2 units. Conversely, ultra-long nanopore reads are an exceedingly valuable tool, when it comes to the generation of deeply characterized reference samples and to resolve complex structural variation [7, 81, 82]. These approaches thus complement each other to investigate the KIV-2 genetics at scale.

In general, we observed a considerable improvement of copy number prediction after correcting for haplotype coverage. This was most pronounced in the larger alleles, which may suggest that especially some larger KIV-2 alleles consist of multiple identical units. These may have originated from relatively recent repeat expansion and only slow divergence of the KIV-2 VNTR. Very little is known about the frequency and mechanisms of KIV-2 expansions [23]. Boerwinkle et al. report generation of one new allele in 376 meioses [83], but no other reports are available. UMI-ONT-Seq allows for the first time to study the mutational and evolutionary mechanisms of this complex VNTR with single nucleotide, respectively single haplotype resolution at scale.

Given its direct portability to other VNTRs with similar structure, UMI-ONT-Seq provides a novel instrument with general applicability beyond *LPA.* It complements and expands similar approaches like circularization-based concatemeric consensus sequencing (R2C2) [84] or linked-read sequencing. R2C2 with UMIs recently reported up to Q50 quality for 550 and 1200 bp long amplicons using cluster sizes 12–17 [85], but might be limited to smaller amplicons, as the coverage on concatemerized targets is also a function of the target length. Conversely, linked-reads can present technical difficulties when analyzing highly similar amplicons [33]. Further use cases for UMI-ONT-Seq may include mapping of epistatic protein mutations in in vitro evolution experiments and deep mutational scans, monitoring of intra-host disease evolution, immune repertoire mapping, mapping of large inserts for massive parallel reporter assays, generation of reference sequences for complex regions, and any other applications requiring precise long consensus sequences and/or SNP phasing at clonal resolution down to 1% [33, 74, 86, 87].

## Conclusions

Using the *LPA* KIV-2 VNTR as a model of a highly complex, challenging, and medically relevant VNTR, we demonstrate the capability of amplicon-based UMI-ONT-Seq to accurately detect mutations, determine the full-length SNP haplotype of each VNTR unit, and determine the VNTR copy number using coverage-corrected haplotypes in recombinant standards, human validation samples and multi-ancestry samples from 1000G. This provides a new, straightforward approach to map variation in such challenging regions. The use of an amplicon-based approach circumvents costly and laborious high molecular weight DNA WGS and provides an efficient method to generate reference data for complex regions with clonal resolution at scale. This will enable researcher to move from limited insights from small multi-ancestry datasets to a much-needed comprehensive picture at scale of the variability in the *LPA* KIV-2 and other similarly complex regions.

## Supplementary Information

Additional file 1. Further notes on quality control of the amplification, additional description of methods, all Supplementary Figures and Supplementary Tables that are mentioned in the manuscript. Supplementary Notes: UMI-ONT-Seq showed negligible to absent co-amplification of other KIV units and no detectable amplification bias of specific KIV-2 repeats. Supplementary Methods: Human samples for validation; Mutation density of KIV units; Testing for co-amplification of other KIV units; Extraction of the KIV-2 intronic CA STR; Detailed PCR Protocols; Quality control of the UMI-amplicons; Ligation sequencing amplicons – Native Barcoding Kit V14 and R9. Supplementary Figures: Fig. S1: Principle of the plasmid mixtures containing KIV-2A and KIV-2B inserts in different ratios that were used for benchmarking UMI-ONT-Seq. Fig. S2: Distribution of the number of KIV units per allele, KIV-2 copy number and Lp(a) per sample of 15 European samples from the SAPHIR study. Fig. S3: Flow chart of the UMI-ONT-Seq analysis pipeline. Fig. S4: Concept of the UMI-ONT-Seq based extraction of unique haplotypes per sample. Fig. S5: Variant detection in plasmid mixtures with the UMI-ONT-Seq analysis pipeline using default clustering (left column) or cluster splitting strategy (right column) for the R9 chemistry and HAC basecalling. Fig. S6: Dataset Q-score estimation using KIV-2A and KIV-2B containing plasmids for the R9 chemistry and HAC basecalling algorithm. Fig. S7: Distribution of the consensus sequence Q-score depending on the minimal cluster threshold for all sequencing conditions of the 4 unmixed KIV-2A or KIV-2B plasmids (both PCR2645 and PCR5104). Fig. S8: Performance measures for the R9 chemistry and HAC basecalling algorithm for 15 human gDNA samples (SAPHIR). Fig. S9: Performance of the nanopore-specific low-level variant caller ClairS-TO for 63 genomic DNA samples (15 samples from SAPHIR, 48 samples of 4 populations from 1000G) compared to conventional KIV-2 NGS (SAPHIR, turquoise) and variants derived from publically available WGS data (1000G, red). Fig. S10: Correlation of the variant levels in standard UMI-free ONT-Seq and in UMI ONT Seq with levels determined by deep KIV-2 NGS for all sequencing chemistries and basecalling algorithms. Fig. S11: Bland–Altman plot for ONT-Seq and UMI-ONT-Seq compared to NGS for all sequencing chemistries and basecalling algorithms used. Fig. S12: Correlation of UMI-ONT-Seq estimated and actual number of KIV-2 repeats measured by ddPCR (expected) and haplotype cluster size distribution of 15 human gDNA samples and two 100% KIV-2A or KIV-2B plasmid. Fig. S13: Occurrence of the different haplotypes for the 15 SAPHIR samples. Fig. S14: Correlation of UMI-ONT-Seq and ddPCR based number of KIV-2 repeats and Venn diagrams showing the haplotypes for 3 trios. Fig. S15: Bland–Altman plot of the 48 1000G samples analyzed with UMI-ONT-Seq compared variant levels derived from publically available WGS data. Fig. S16: SNP density of the KIV domains and variant levels in the KIV-2. Fig. S17: Correlation of expected (ddPCR) and UMI-ONT-Seq predicted number of KIV-2 for different steps of the haplotype extraction and number of KIV-2 repeat prediction in 1000G. Fig. S18: Length distribution (relative occurrence) of the extracted STR-region of 63 gDNA samples of 5 different populations (1000G and SAPHIR) and the two unmixed plasmids. Fig. S19: KIV-2 subtype specific haplotype diversity of all 63 human gDNA samples in the present study (1000G, SAPHIR). Fig. S20: Classification of KIV-2A repeat subclusters using 3 positions (35, 3103, 4358) of a previously suggested haplotype in a multi-ancestry sample set (1000G and SAPHIR) of 63 samples. Fig. S21: Classification of KIV-2B and KIV-2C repeats into three main subclusters using 5 positions (50, 2409, 5037, 5045, 5052) in a multi-ancestry sample set (1000G and SAPHIR) of 63 samples. Fig. S22: Classification of KIV-2C containing repeats with the commonly used KIV-2 subtype defining positions (594, 621, 666) in a multi-ancestry sample set (1000G and SAPHIR) of 63 samples. Fig. S23: Classification of the two most frequent disease relevant variants in the KIV-2 VNTR (4925G > A: red and 4733G > A: green) in a multi-ancestry sample set (1000G and SAPHIR) of 63 samples. Supplementary Tables: Table S1: ddPCR validation of the two unmixed and the plasmid mixtures for both fragments. Table S2: ddPCR and qPCR reaction conditions. Table S3: Primer sequences. Table S4: PCR conditions for the 2645 and the 5104 fragment. Table S5: Summary statistics of the sequencing results per kit and basecalling algorithm. Table S6: Sequencing overview R9 HAC. Table S7: Sequencing overview V14 HAC. Table S8: Sequencing overview V14 SUP. Table S9: Sequencing overview V14 SUPDUP. Table S10: Performance measures for all plasmid mixtures analyzed with the initial version of the pipeline. Table S11: Edit distances within UMI sequences in the same UMI cluster for the original ONT pipeline. Table S12: Variant levels and noise of variant level using UMI-ONT-Seq with the default clustering strategy. Table S13: Performance measures for all plasmid mixtures analyzed with by UMI-ONT-Seq with the cluster splitting strategy. Table S14: Noise reduction of detected variant levels of the UMI-ONT-Seq analysis pipeline using either the default clustering or the cluster splitting strategy. Table S15: Variant levels and noise of variant level of the UMI-ONT-Seq analysis pipeline using the cluster splitting strategy. Table S16: Threshold values for reaching a dataset Q-score of Q40. Table S17: Consensus sequence summary statistics for a minimal cluster size threshold of 6 reads per UMI cluster. Table S18: Summary of the error profiles for all cluster thresholds greater than 10. Table S19: Dataset Q-scores per sequencing chemistry and basecalling algorithm after error-profile adjustment. Table S20: Performance measures for all human gDNA samples for all sequencing chemistries and basecalling conditions. Table S21: Summary performance measures for UMI-ONT-Seq. Table S22: Summary performance measures for ONT-Seq without UMIs. Table S23: Differences and correlation values between ddPCR measured and UMI-ONT-Seq predicted number of KIV-2 repeats for different prediction strategies. Table S24: Overview of the three trios, including number of consensus sequences, uniquely occurring haplotypes (used for the Venn diagram), the KIV-2 copy number estimation based on the coverage-corrected haplotypes and the results of the ddPCR. Table S25: KIV-2 subtype occurrence in the SAPHIR and 1000G dataset. Table S26: KIV-2 subtype specific summary performance measures for UMI-ONT-Seq analysis of the 48 1000G samples. Table S27: Per-sample performance measures for UMI-ONT-Seq analysis of the 48 1000G samples for V14 SUP. Table S28: Summary performance measures for UMI-ONT-Seq analysis of the 48 1000G samples without KIV-2B specific variants. Table S29: Correlation between the number of KIV-2 repeats quantified by ddPCR and by UMI-ONT-Seq for each step of the UMI-ONT-Seq KIV-2 quantification algorithm in the 1000G dataset. Table S30: Extracted STR sequences of all 63 human gDNA samples and two unmixed KIV-2A, respectively KIV-2B plasmids. Table S31: Population-specific degeneration of the STR region. Table S32: Diversity of the extracted STR sequences per population. Table S33: Predicted number of KIV-2 repeats for 48 samples and the three trios of the 1000G project using either UMI ONT Seq, ddPCR or the results from Behera et al. Table S34: Number of KIV unit specific sequence patterns found in the raw reads of the 48 1000 Genome samples.

Additional file 2. A summary of all variants found across the European SAPHIR sample set and the four 1000G populations, their absolute occurrence in the different populations, mean and standard deviation of the variant level per population.

Additional file 3. Detailed per-sample overview of all found variants and their variant level for each of the 48 1000G samples.

## Data Availability

The UMI-ONT-Seq analysis pipeline is available at https://github.com/genepi/umi-pipeline-nf/tree/v0.2.1. All code used for data analysis is available at https://github.com/AmstlerStephan/UMI-ONT-Seq_Analysis and https://github.com/AmstlerStephan/haplotyping-KIV2-nf/tree/v0.1.0. The 1000G samples are available at the Coriell Sample Repository. All sequencing data generated for the plasmid samples and the 1000G samples is available in the European Nucleotide repository (ENA), https://www.ebi.ac.uk/ena/browser/view/PRJEB73509 (ENA project ID PRJEB73509) [47]. The datasets generated for SAPHIR are not publicly available due restrictions in the informed consent but are available from the corresponding author on reasonable request.

## Abbreviations

CNV: Copy number variation
VNTR: Variable number tandem repeat
STR: Short tandem repeat
Apo(a): Apolipoprotein(a)
Lp(a): Lipoprotein(a)
KIV: Kringle IV
KIV-2: KIV type-2
HMW: High molecular weight
LMW: Low molecular weight
LD: Linkage disequilibrium
ONT: Oxford Nanopore Technologies
UMI: Unique molecular identifiers
HAC: High accuracy
SUP: Super accuracy
SUPDUP: Super accuracy with duplex
NGS: Next-generation sequencing
UMI-ONT-Seq: Nanopore sequencing with unique molecular identifiers
SAPHIR: Salzburg Atherosclerosis Prevention Program in subjects at High Individual Risk
1000G: 1000 Genome Project
ddPCR: Droplet digital PCR
CI: Confidence interval
LSP: Locus-specific primer
UVP: Universal primer
WGS: Whole-genome sequencing
YRI: Yoruba
CDX: Dai Chinese
JPT: Japanese
PJL: Punjabi
EUR: European
