# Supplementary material for: Nanopore sequencing with unique molecular identifiers enables accurate mutation analysis and haplotyping in the complex lipoprotein(a) KIV-2 VNTR
Source: Genome Med. 2024 Oct 8;16:117. doi: 10.1186/s13073-024-01391-8 (PMC11462820; doi:10.1186/s13073-024-01391-8)
Supplement: Supplementary file 1 — Additional file 1. Further notes on quality control of the amplification, additional description of methods, all Supplementary Figures and Supplementary Tables that are mentioned in the manuscript. Supplementary Notes: UMI-ONT-Seq showed negligible to absent co-amplification of other KIV units and no detectable amplification bias of specific KIV-2 repeats. Supplementary Methods: Human samples for validation; Mutation density of KIV units; Testing for co-amplification of other KIV units; Extraction of the KIV-2 intronic CA STR; Detailed PCR Protocols; Quality control of the UMI-amplicons; Ligation sequencing amplicons – Native Barcoding Kit V14 and R9. Supplementary Figures: Fig. S1: Principle of the plasmid mixtures containing KIV-2A and KIV-2B inserts in different ratios that were used for benchmarking UMI-ONT-Seq. Fig. S2: Distribution of the number of KIV units per allele, KIV-2 copy number and Lp(a) per sample of 15 European samples from the SAPHIR study. Fig. S3: Flow chart of the UMI-ONT-Seq analysis pipeline. Fig. S4: Concept of the UMI-ONT-Seq based extraction of unique haplotypes per sample. Fig. S5: Variant detection in plasmid mixtures with the UMI-ONT-Seq analysis pipeline using default clustering (left column) or cluster splitting strategy (right column) for the R9 chemistry and HAC basecalling. Fig. S6: Dataset Q-score estimation using KIV-2A and KIV-2B containing plasmids for the R9 chemistry and HAC basecalling algorithm. Fig. S7: Distribution of the consensus sequence Q-score depending on the minimal cluster threshold for all sequencing conditions of the 4 unmixed KIV-2A or KIV-2B plasmids (both PCR2645 and PCR5104). Fig. S8: Performance measures for the R9 chemistry and HAC basecalling algorithm for 15 human gDNA samples (SAPHIR). Fig. S9: Performance of the nanopore-specific low-level variant caller ClairS-TO for 63 genomic DNA samples (15 samples from SAPHIR, 48 samples of 4 populations from 1000G) compared to conventional KIV-2 NGS (SAPHIR, turq [file 13073_2024_1391_MOESM1_ESM.pdf]

Supplementary Materials for

Nanopore sequencing with unique molecular identifiers enables accurate mutation analysis and haplotyping in the complex Lipoprotein(a) KIV-2 VNTR

Stephan Amstler [a], Gertraud Streiter [a], Cathrin Pfurtscheller [a], Lukas Forer [a], Silvia Di Maio [a],
Hansi Weissensteiner[a], Bernhard Paulweber [b], Sebastian Schönherr [a],
Florian Kronenberg [a], Stefan Coassin [a] *

[a] Institute of Genetic Epidemiology, Medical University of Innsbruck, Innsbruck, Austria
[b] First Department of Internal Medicine I, Paracelsus Medical University, Salzburg, Austria

This PDF file includes:

Supplementary Notes
Supplementary Methods
Supplementary Figures 1 - 23
Supplementary Tables 1 - 34

Table of Content

SUPPLEMENTARY NOTES **3**

UMI-ONT-SEQ SHOWED NEGLIGIBLE TO ABSENT CO-AMPLIFICATION OF OTHER KIV UNITS AND NO DETECTABLE AMPLIFICATION BIAS OF SPECIFIC KIV-2 REPEATS 3

SUPPLEMENTARY METHODS **4**

HUMAN SAMPLES FOR VALIDATION 4

MUTATION DENSITY OF KIV UNITS 4

TESTING FOR CO-AMPLIFICATION OF OTHER KIV UNITS 4

EXTRACTION OF THE KIV-2 INTRONIC CA STR 5

DETAILED PCR PROTOCOLS 5

QUALITY CONTROL OF THE UMI-AMPLICONS 6

LIGATION SEQUENCING AMPLICONS – NATIVE BARCODING KIT V14 AND R9 6

SUPPLEMENTARY FIGURES **7**

SUPPLEMENTARY TABLES **30**

REFERENCES **58**

Supplementary Notes

UMI-ONT-Seq showed negligible to absent co-amplification of other KIV units and no detectable amplification bias of specific KIV-2 repeats

To test for any co-amplification of other KIV units we screened the raw sequencing reads of the 48 1000G samples for KIV unit specific sequence patterns. In 9,019,607 reads we did not find any patterns originating from KIV-4, only 1 for KIV-1 and only 146 to 341 reads for KIV-3, which corresponds to 0.0017% to 0.0038% (Table S34). Overall, the co-amplification level appeared negligible to absent. Noteworthy, KIV-3 amplicons (6,007 bp) would be automatically excluded in the UMI-ONT-Seq analysis pipeline, as the raw sequencing reads are filtered to have ≥95 % overlap with the KIV-2 reference sequence (5,104 bp).

Moreover, to test for any amplification bias of KIV-2 subtypes we used the level KIV-2B haplotypes, which was shown to be non-uniform and underrepresented in NGS[1]. In UMI-ONT-Seq, the level of KIV-2B haplotypes rose considerably compares to NGS and was uniform across the whole KIV-2B haplotype (see Additional file 2 and Additional file 3). This indicates that UMI-ONT-Seq likely reflects their fractional representation more accurately than NGS. Also, the UMI-ONT-Seq KIV-2B haplotypes in the plasmid mixtures matched the expected mixture levels closely (Figure 2F), indicating that there is no (at least major) amplification bias.

Overall, we observed a negligible to absent co-amplification of the other KIV units and a more even sequencing depth and representation of KIV-2B haplotypes compared to NGS.

Supplementary Methods

Human samples for validation

Samples were drawn from the healthy-working population SAPHIR (Salzburg Atherosclerosis Prevention Program in subjects at High Individual Risk)[2]. SAPHIR is an observational study conducted in the years 1999-2002 involving healthy unrelated subjects (645 females from age 39 to 67 and 1093 males from age 39 to 66). Study participants were recruited by health screening programs in companies in area of Salzburg, Austria. For KIV-2 quantification by ddPCR 40 ng template DNA was digested with 2 u EcoRI for 1 h at 37°C and genomic KIV-2 number was assessed on a Biorad QX-200 ddPCR system using the KIV-2 quantification primer/probe set from Lanktree et al[3] (Table S2). Three ddPCR replicates per sample were merged for precise KIV-2 counting[4].

Mutation density of KIV units

The PCR5104 product starts 561 bp upstream of the first KIV-2 exon and ends 159 downstream of the second exons (as provided by the Exons Table in Ensembl entry ENST00000316300.10). We thus extracted the coordinates of every KIV unit, starting 561 bp upstream of the first exon and ending 159 bp downstream of the second exon. We then retrieved the public SNP data for each of these regions for the same 1000G samples that were investigated in our study. To get the actual number of variants for the KIV-2 VNTR we multiplied the variant level per variant with the number of KIV-2 repeats per sample (based on ddPCR). We normalized the number of found variants for the other KIV units by dividing the number of found variants by the length of the KIV units (based on the extraction coordinates) and for the KIV-2 VNTR by the PCR5104 amplicon length multiplied by the number of KIV-2 repeats (based on ddPCR).

Testing for co-amplification of other KIV units

We aligned the LSP primer sequences to the genomic LPA sequence using SnapGene v7.2.1, allowing a mismatch every 4 bases (the most permissive setting possible). Degenerated priming sites in correct orientation are found only in KIV-1, KIV-3 (one identical priming site) and KIV-4. We aligned the putative unspecific PCR products for KIV-1, KIV-3 and KIV-4 with the intended KIV-2 PCR product using ClustalO[5] and inspected the raw sequencing data (the unprocessed fastq files) of the 48 1000G samples for multiple characteristic 20 bp long sequence patterns that differentiate the putative amplicons (Table S34).

Extraction of the KIV-2 intronic CA STR

STR sequences were extracted from the consensus sequences using a custom python script (available at https://github.com/AmstlerStephan/UMI-NPSeq_Analysis). The STR region was defined as reference sequence position 2472-2506. After extraction of this region the actual start and end of the STR was defined by the first and last occurrence of a CA repeat. Like the process of extracting haplotypes, we employed a sample-specific threshold to eliminate highly improbable STR sequences. We determined this threshold based on a binomial distribution with a 0% likelihood for an STR sequence to occur. Any STR sequences occurring below this threshold were excluded from the analysis.

Detailed PCR Protocols

To create the UMI-tagged amplicon library, we adapted and optimized an existing protocol, which is provided by ONT (protocol ID: cpu_9107_v109_reve_09oct2020).

Samples were diluted to contain approximately 50,000 target molecules in 4 µl (1 KIV-2 repeat ≙ 1 target), under maximum case scenario of 80 KIV-2 repeats for the genomic DNA (gDNA) samples (up to 40 copies per allele[6]). Average molecular weight of one base-pair was assumed to be 660 g/mol for double stranded DNA. Genomic DNA samples were normalized to a concentration of 20 ng/µL and then diluted to 50,000 targets by mixing them 1:36 with nuclease free water. Plasmid mixtures, with a concentration of 6.5 ng/µL were diluted to 50,000 targets with a dilution series in four steps (1:16 per step, 1:65536 in total) to have sufficient volume for accurate pipetting. The PCR to generate the amplicon library is separated in three parts, which are a tagging PCR followed by two amplification PCRs (“Early PCR” and “Late PCR”). A detailed PCR protocol can be found in Table S4.

After tagging, the UMI-primers and untagged molecules are digested using ExoCip (New England Biolabs [NEB]; Ipswich, MA, USA; E1050L), mixing 3 µL ExoCip A, 3 µL ExoCip B and 15 µL of the PCR product. The protocol was run at 4 min digestion (37°C) and 4 min inactivation (80°C).

After the “Early-PCR” the amplicons were purified by adding 45 µL AMPure XP beads (Beckman Coulter; Indianapolis, Indiana USA; 19836400) and 10 min incubation on a Hula mixer. The beads were pelleted on a magnet and washed twice with 200 µL 70 % ethanol. After spinning down, re-pelleting and removal of the residual ethanol, the DNA was eluted in 21 µL nuclease free water with 5 min incubation.

After the “Late PCR” the amplicons were again purified with AMPure XP beads as described before but eluted in 30 µL nuclease free water.

Quality control of the UMI-amplicons

To ensure the quality and target specificity of the UMI amplicons the concentration of the all samples was measured using a Qubit 3 (Thermo Scientific; Waltham, MA USA; Q33238) fluorometer with the DNA high sensitivity kit (Thermo Scientific; Waltham, MA USA; Q33231). Additionally, 3 µL of the PCR product of the "Late PCR" (prior the final clean up) were loaded onto an 1% agarose gel. As size reference an 1.5 µL of GeneRuler 1 kb ladder (Thermo Scientific; Waltham, MA USA; SM0311) was used.

Ligation sequencing amplicons – Native Barcoding Kit V14 and R9

The ONT protocols for both chemistries were followed (R9: SQK-LSK109, EXP-NBD104 and EXP NBD114, protocol version: NBA_9093_v109_revK_12Nov2019; V14: SQK NBD114.24, protocol version: NBA_9168_v114_revD_15Sep2022). We used 250 fmol for V14 and 200 fmol for R9 as input for the end prep of the DNA. After library preparation the final sequencing mix that is loaded onto the flow cell should contain was adjusted to contain 20 fmol of the library.

Supplementary Figures

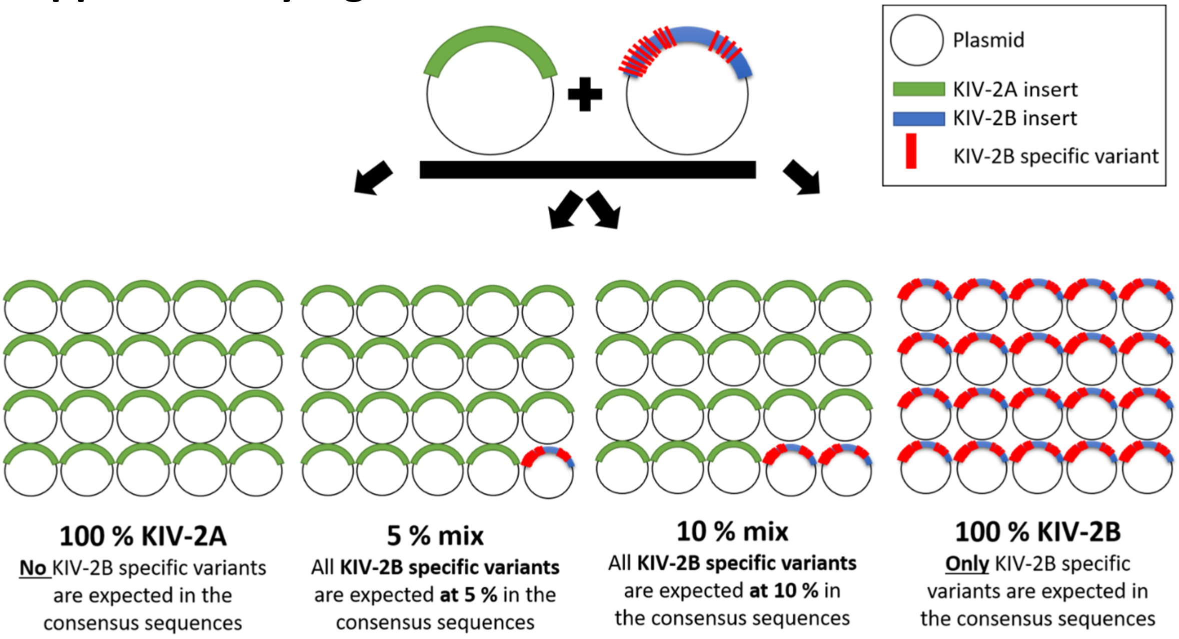

Fig S1: Principle of the plasmid mixtures containing KIV-2A and KIV-2B inserts in different ratios that were used for benchmarking UMI-ONT-Seq.

The plasmids were created using KIV-2 specific amplicons spanning either the inter-kringle intron (2645 insert; amplicon PCR2645) or the intra-kringle intron (5104 bp insert; amplicon PCR5104) of either KIV-2 subtype A (KIV-2A, green) or KIV-2 subtype B (KIV-2B, blue). Generation of the plasmids has been detailed in [1]. KIV-2A and KIV-2B plasmids were mixed at different ratios (100:0 to 95:5 and 0:100), with the KIV-2B comprising the minor fraction to resemble the in-vivo situation. Each KIV-2B insert differs from the reference at 85 (PCR5104) and 116 (PCR2645) positions (red lines). These differences are present all on the same molecule, representing thus one haplotype. Therefore, all KIV-2B positions shall occur at the same level in the consensus sequences. The Figure exemplifies mixtures for unmixed (100 %) KIV-2A, unmixed (100%) KIV-2B, 5 % KIV-2B mixture and 10 % KIV-2B mixture.

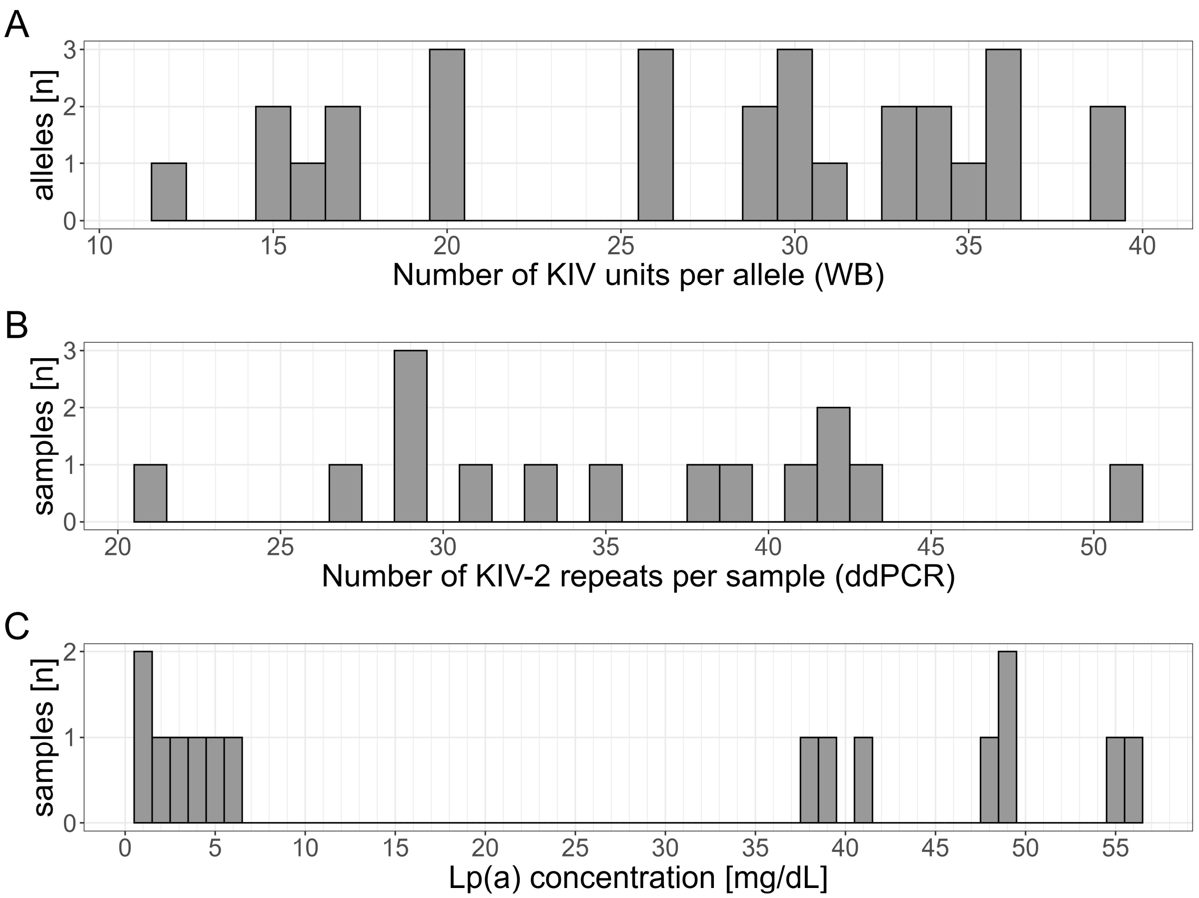

Fig S2: Distribution of the number of KIV units per allele, KIV-2 copy number and Lp(a) per sample of 15 European samples from the SAPHIR study.

Panel A: The number of KIV units per allele from apo(a) Western Blot (WB). The allele length covers the broad range of possible allele lengths, with an enrichment of extreme alleles. **Panel B**: The KIV-2 copy number per sample. The sum of KIV-2 repeats show an even distribution across the 15 samples. **Panel C:** Lp(a) concentrations per sample. The distribution of Lp(a) concentrations reflects the expected pattern based on allele distribution and Lp(a) lowering mutations included in the sample set. Phenotype data from [1, 7].

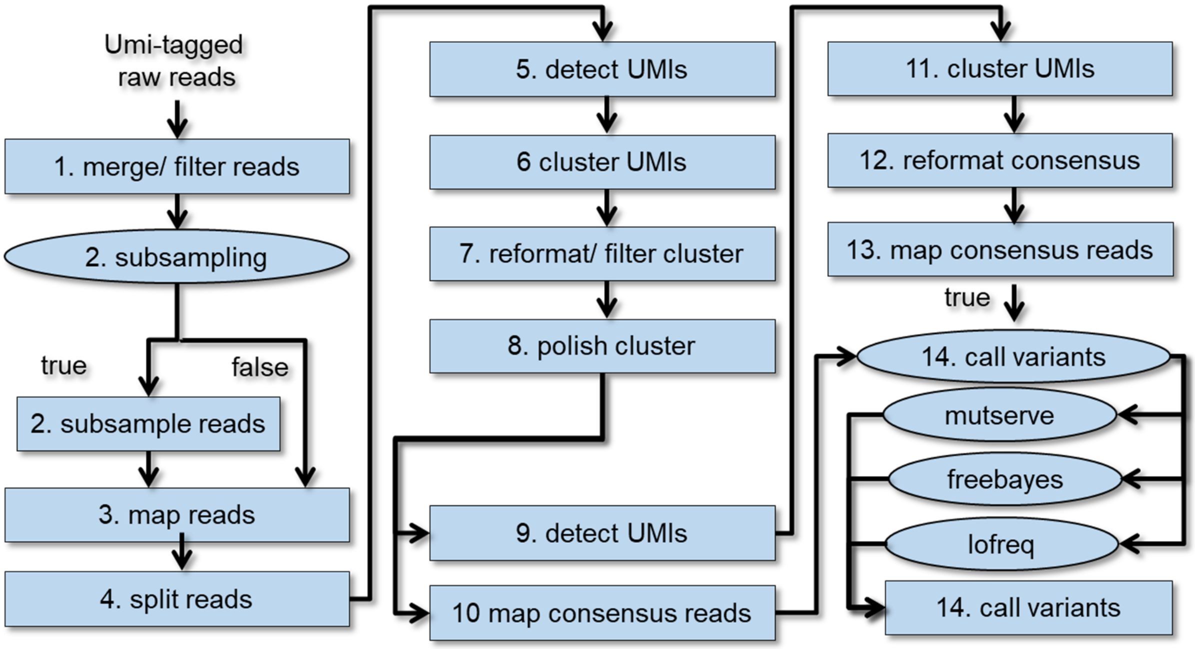

Fig S3: Flow chart of the UMI-ONT-Seq analysis pipeline.

The analysis workflow 1. merges and filters all FASTQ files per sample, 2. Performs an optional read subsampling step, 3. Maps the reads against the reference sequences, 4. Splits the aligned reads to keep only full-length reads with UMI-tags on both sides, 5. Extracts the UMI-sequences in the reads, 6. Clusters them according to the UMI-sequences, 7. Filters the UMI-clusters to keep only reads above a minimal cluster size threshold and 8. polishes each cluster of UMI-sequences to obtain one consensus sequence per cluster. 9. Detection, 11. clustering and 12. consensus read creation is repeated. After cluster polishing (8) and reformat consensus (12) the consensus sequences are mapped against the reference (10 and 13) for 14. variant calling with mutserve[8] (alternatively also Freebayes[9] (v1.3.2) or Lofreq[10] (v2.1.5) can be invoked).

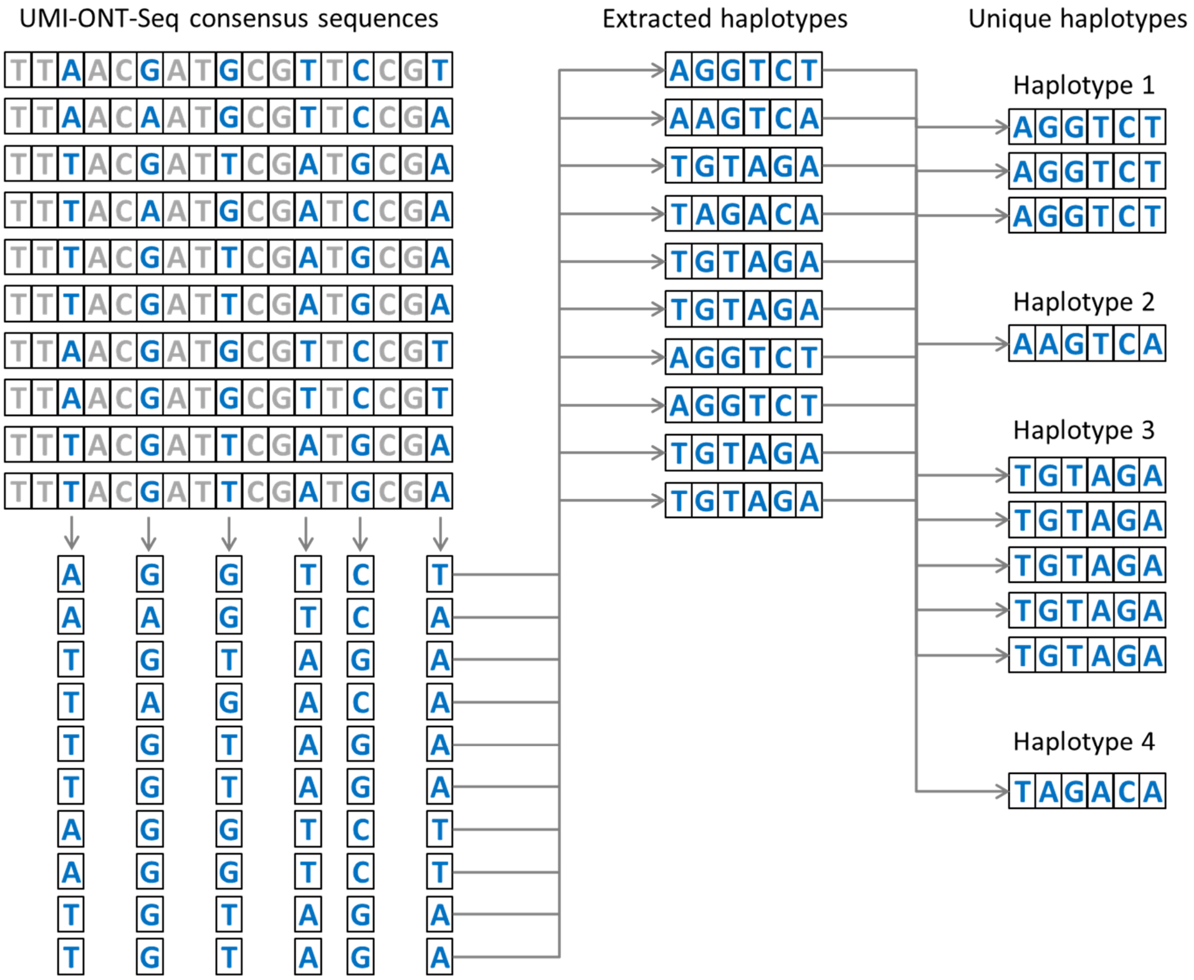

Fig S4: Concept of the UMI-ONT-Seq based extraction of unique haplotypes per sample.

The polymorphic positions of the UMI-ONT-Seq based consensus sequences are extracted and represent the KIV-2 haplotypes of each sample. The unique haplotypes are then derived by clustering of all haplotypes based on their sequence.

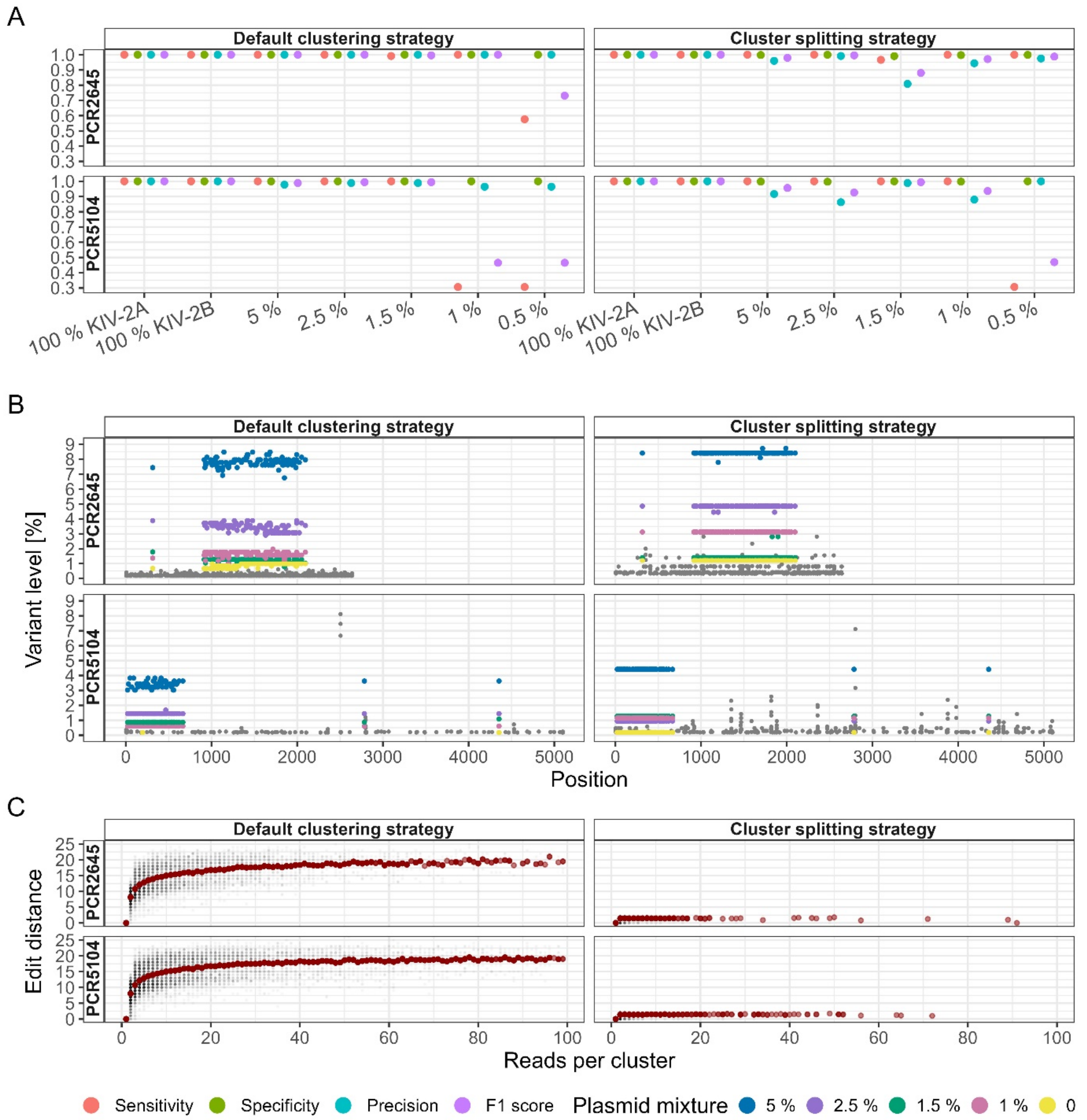

Fig S5: Variant detection in plasmid mixtures with the UMI-ONT-Seq analysis pipeline using default clustering (left column) or cluster splitting strategy (right column) for the R9 chemistry and HAC basecalling.

Panel A: Variant recall performance measures for the UMI-ONT-Seq analysis pipeline for different plasmid mixtures (KIV-2B in KIV-2A background, 0.5 – 100 %) and both amplicons (PCR2645, PCR5104). Down to 0.5 % percent variant level performance values were close to 100 % for all conditions. **Panel B:** detected variant levels of the UMI-ONT-Seq analysis pipeline using default clustering or cluster splitting strategy for the plasmid mixtures from 0.5 – 5 % across every position of both fragments. Low-level residual noise (grey points) led to a lower detection limit of ~0.85 % variant level. Variance of detected variant levels of up to ±1 % (blue points, 5 % mixture) was observed for the default clustering strategy. **Panel C** shows the edit distance of the UMI-sequences for different UMI cluster sizes for two approaches. Using default clustering strategy, UMI-sequences with an edit distance up to 25 were found in a single cluster. Using the cluster splitting strategy reduced the maximal edit distance per cluster to ≤ 2.

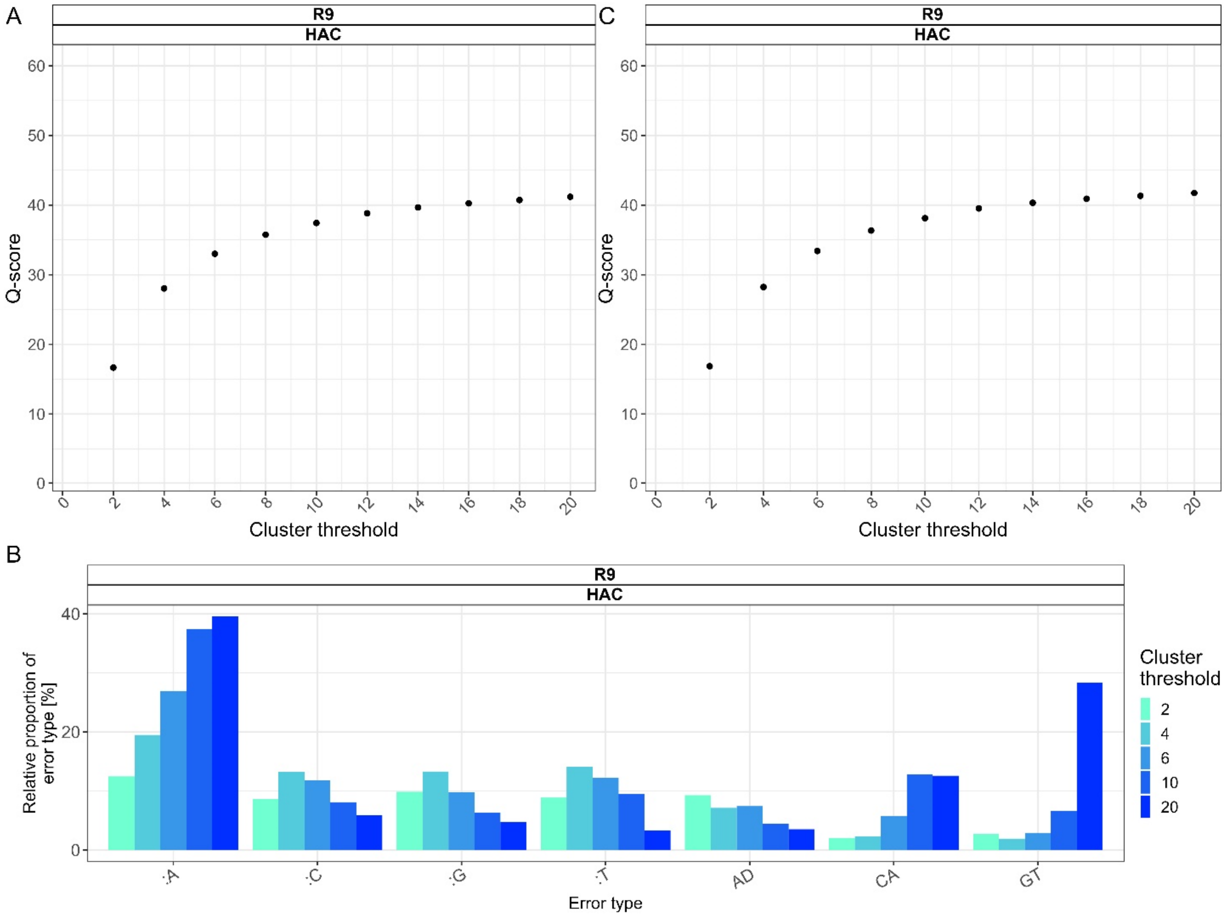

Fig S6: Dataset Q-score estimation using KIV-2A and KIV-2B containing plasmids for the R9 chemistry and HAC basecalling algorithm.

Panel A: Dataset Q-score of the consensus sequences. **Panel B:** Characteristic errors for the R9 HAC condition. Like ONT-Seq without UMIs, insertion are the main characteristic error (40%) also in R9 UMI-ONT-Seq at all cluster size thresholds. **Panel C:** Dataset Q-score per cluster size threshold after correction for low-level transversions (G to T and C to A). Correction for these errors caused marginal increase of the dataset Q-Score.

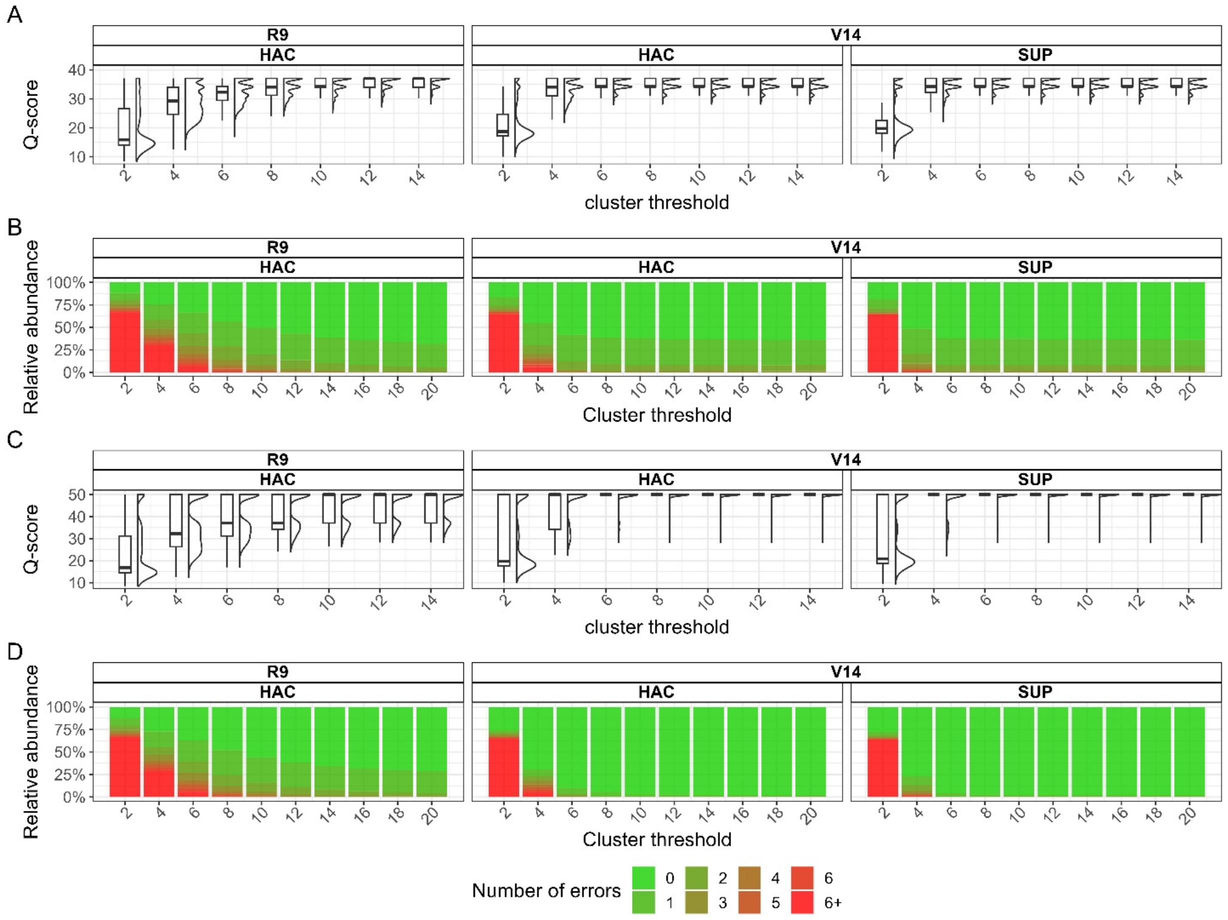

Fig S7: Distribution of the consensus sequence Q-Score depending on the minimal cluster threshold for all sequencing conditions of the 4 unmixed KIV-2A or KIV-2B plasmids (both PCR2645 and PCR5104).

Panel A: Consensus sequence Q-score for each chemistry and sequencing condition depending on the minimal cluster size threshold chosen for the analysis. While for the R9 chemistry the median Q-score increased steadily up to a minimal cluster size of 12, the V14 chemistries median Q-score increased rapidly, reaching the maximal per-read Q-score already at 6 reads per cluster (limited by the fragment length). Noteworthy, panel A includes only imperfect reads, since perfect reads would have an infinite Q-score. Therefore, **panel B** shows the percentage of perfect reads depending on the minimal cluster size threshold. The same trend as for the median Q-score is observed also here. The percentage of perfect reads increases steadily. At 12 reads per cluster 48 %, 58 % and 62.5 % of all consensus sequences were perfect for R9 HAC, V14 HAC and V14 SUP respectively. Correcting for characteristic errors (GT and CA conversions, see Results for details), increased median Q-score **(C)** and percentage of perfect reads **(D)** per minimal cluster threshold drastically. At 6 reads per cluster almost 100 % of the reads of both V14 conditions were perfect.

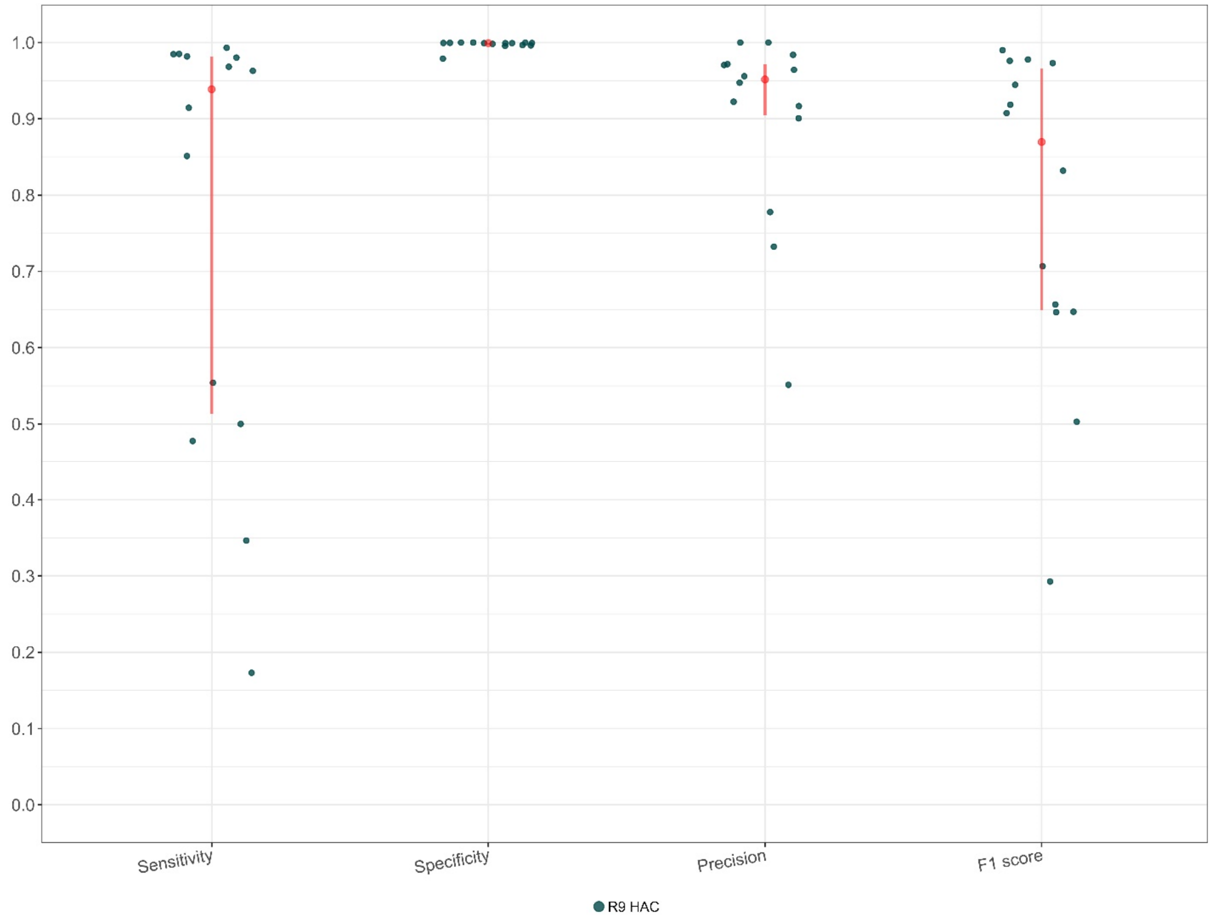

Fig S8: Performance measures for the R9 chemistry and HAC basecalling algorithm for 15 human gDNA samples (SAPHIR).

The red point and line show median values and interquartile range. The blue points are the single samples. Despite having some outliers, the median sensitivity is above 90 %, specificity 100 %, precision above 95 % and F1 score above 85 %.

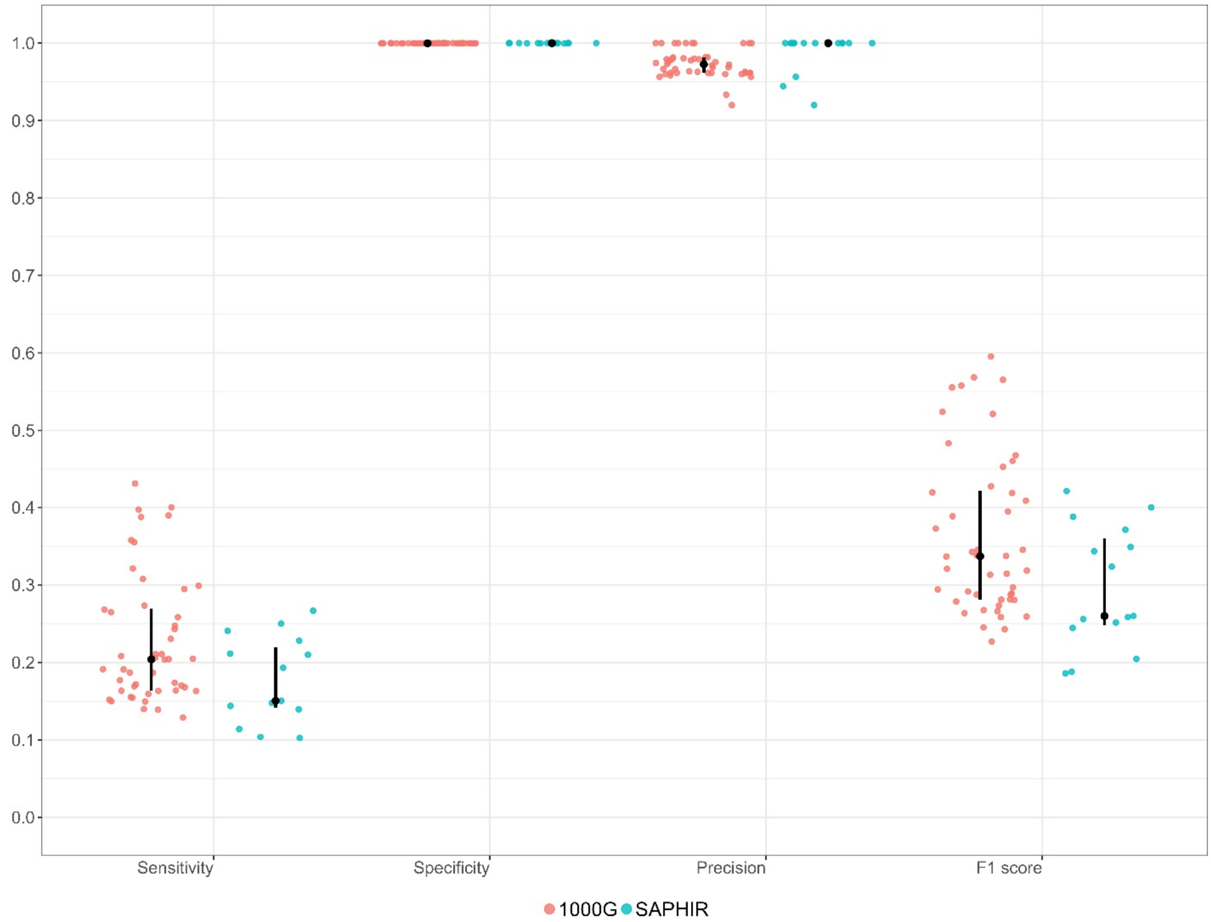

Fig S9: Performance of the nanopore-specific low-level variant caller ClairS-TO for 63 genomic DNA samples (15 samples from SAPHIR, 48 samples of 4 populations from 1000G) compared to conventional KIV-2 NGS[1] (SAPHIR, turquoise) and variants derived from publically available WGS data[1, 11] (1000G, red).

ClairS-TO detected only a fraction of the actual variants of the genomic DNA samples compared, resulting in low median sensitivity (≈21 % for 1000G and ≈15% for SAPHIR). Specificity and precision are high for both sample sets (>95 %), while the low F1 score reflects the low sensitivity values.

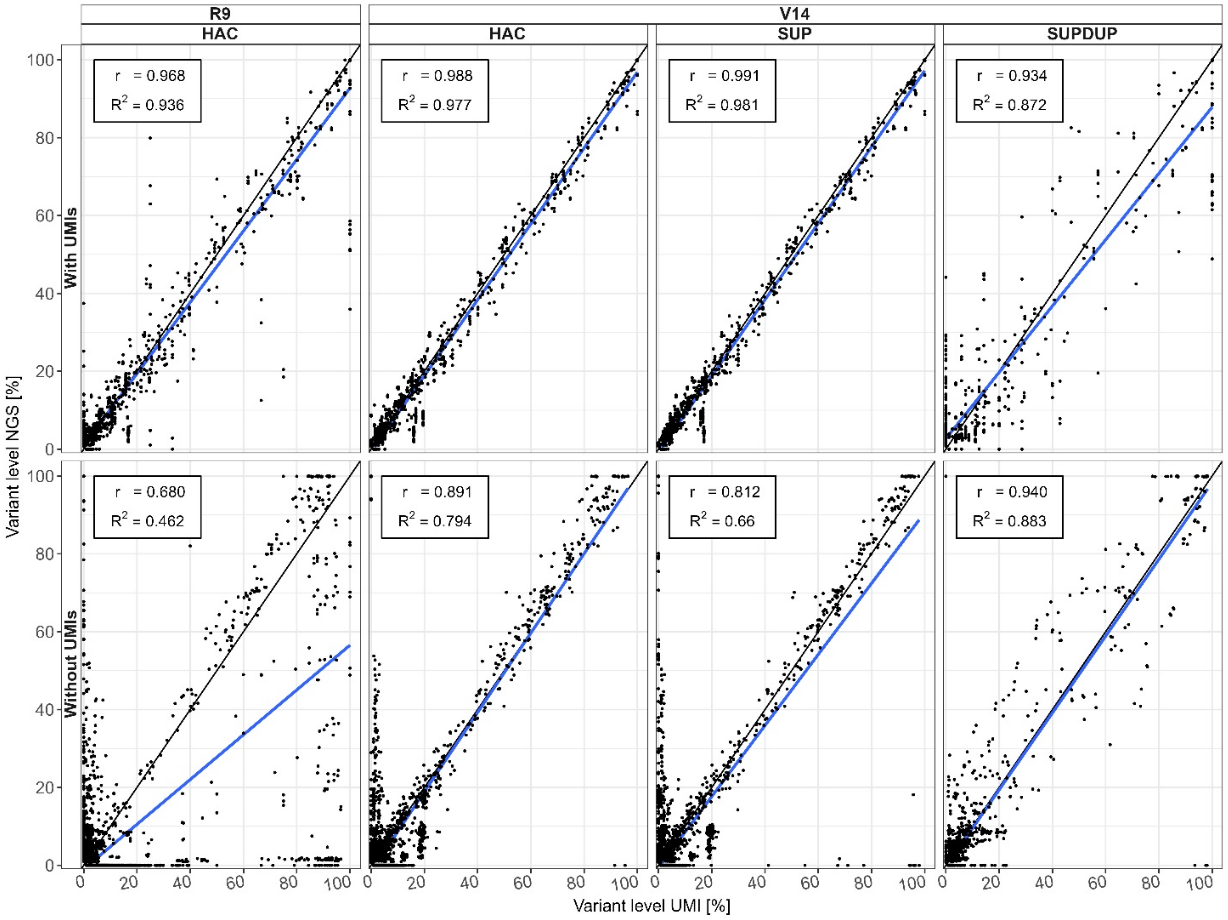

Fig S10: Correlation of the variant levels in standard UMI-free ONT-Seq and in UMI-ONT-Seq with levels determined by deep KIV-2 NGS[1] for all sequencing chemistries and basecalling algorithms.

Black points are variants found in NGS, ONT-Seq (top panels) or UMI-ONT-Seq (bottom panels). Without UMIs (using only the raw reads) we observed increasing correlation with increasing raw read accuracy (lowest to best: R9 HAC, V14 HAC, V14 SUP, V14 SUPDUP). For UMI-ONT-Seq we had generally high correlation between the variant levels compared to NGS for all conditions. V14 HAC and V14 SUP showed the best correlation ≥97 %, while SUPDUP showed lower performance due to the reasons described in the main manuscript.

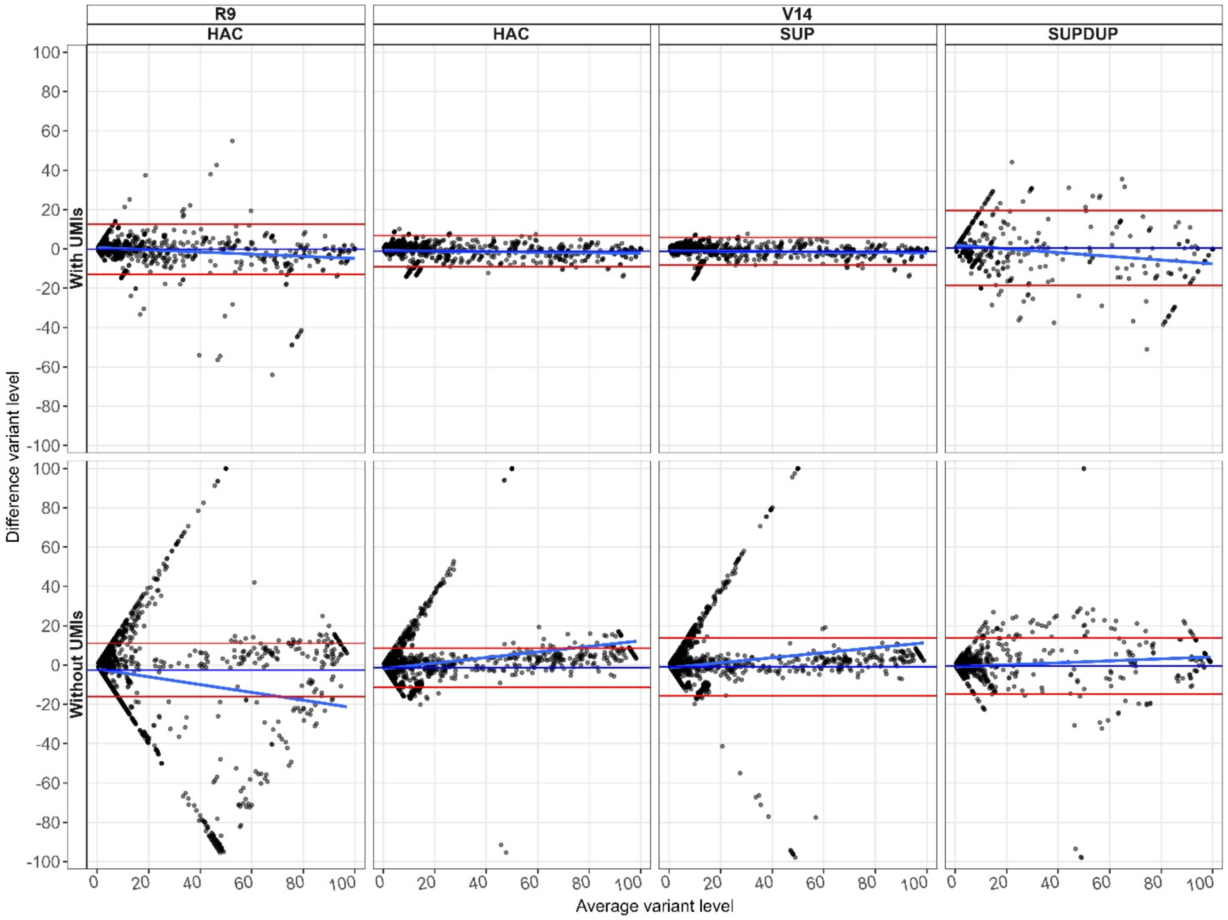

Fig S11: Bland-Altman plot for ONT-Seq and UMI-ONT-Seq compared to NGS for all sequencing chemistries and basecalling algorithms used.

Black points are variants found in NGS, ONT-Seq (top panels) or UMI-ONT-Seq (bottom panels). Without UMIs (using only the raw reads) we observed increasing correlation, decreasing bias for high level variants and narrower lines of agreement (95 % confidence interval) with increasing raw read accuracy (lowest to best: R9 HAC, V14 HAC, V14 SUP, V14 SUPDUP). For UMI-ONT-Seq we had generally high correlation between the variant levels compared to NGS for all conditions, narrower lines of agreement and, for V14 HAC and SUP, also a negligible bias.

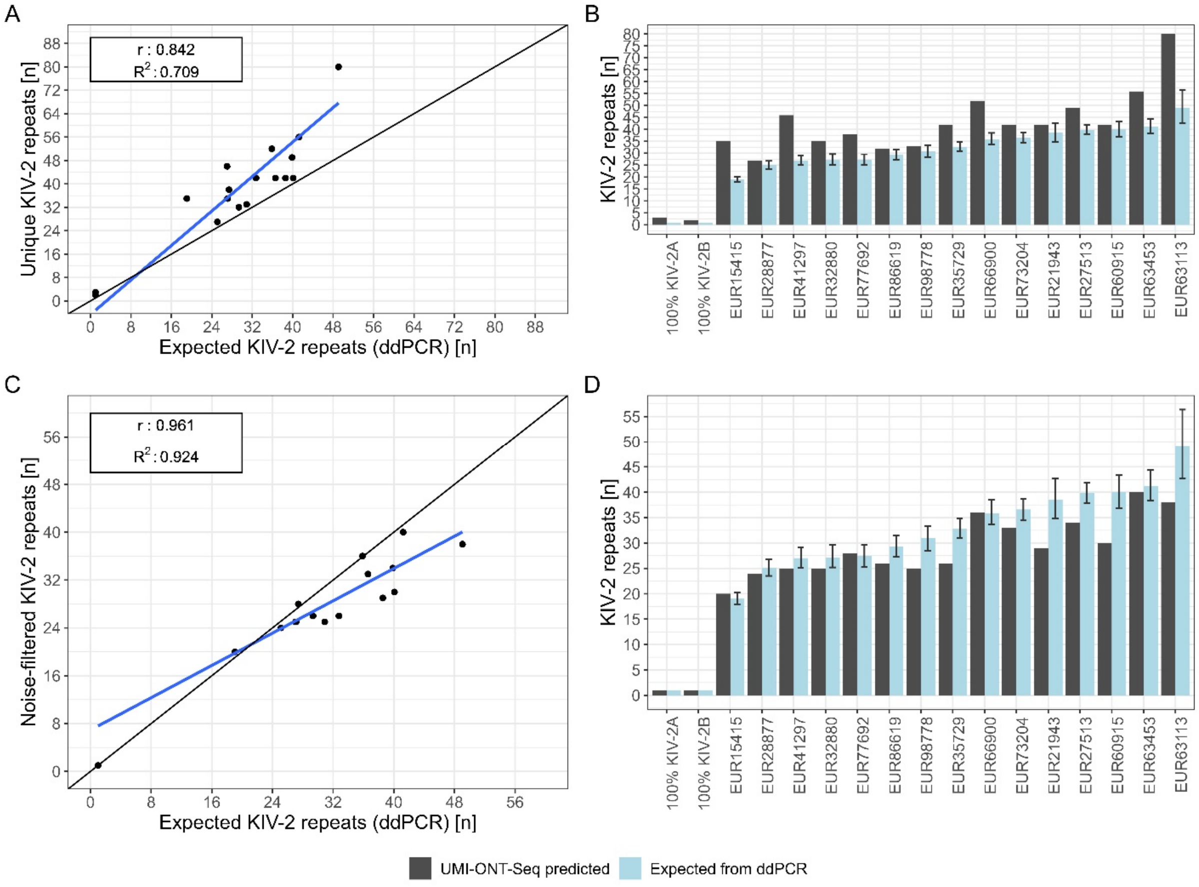

Fig S12: Correlation of UMI-ONT-Seq estimated and actual number of KIV-2 repeats measured by ddPCR (expected) and haplotype cluster size distribution of 15 human gDNA samples and two 100% KIV-2A or KIV-2B plasmid.

Panels A to D show the correlation (**A, C**) and absolute agreement (**B, D**) of the number of KIV-2 repeats derived from the number of unique haplotypes in UMI-ONT-Seq and as expected from ddPCR. Despite high correlation, for the majority of samples the number of KIV-2 repeats is overestimated. **Panels C and D** show the same correlation using a noise-filtering algorithm that removes and merges highly unlikely haplotype cluster sizes.

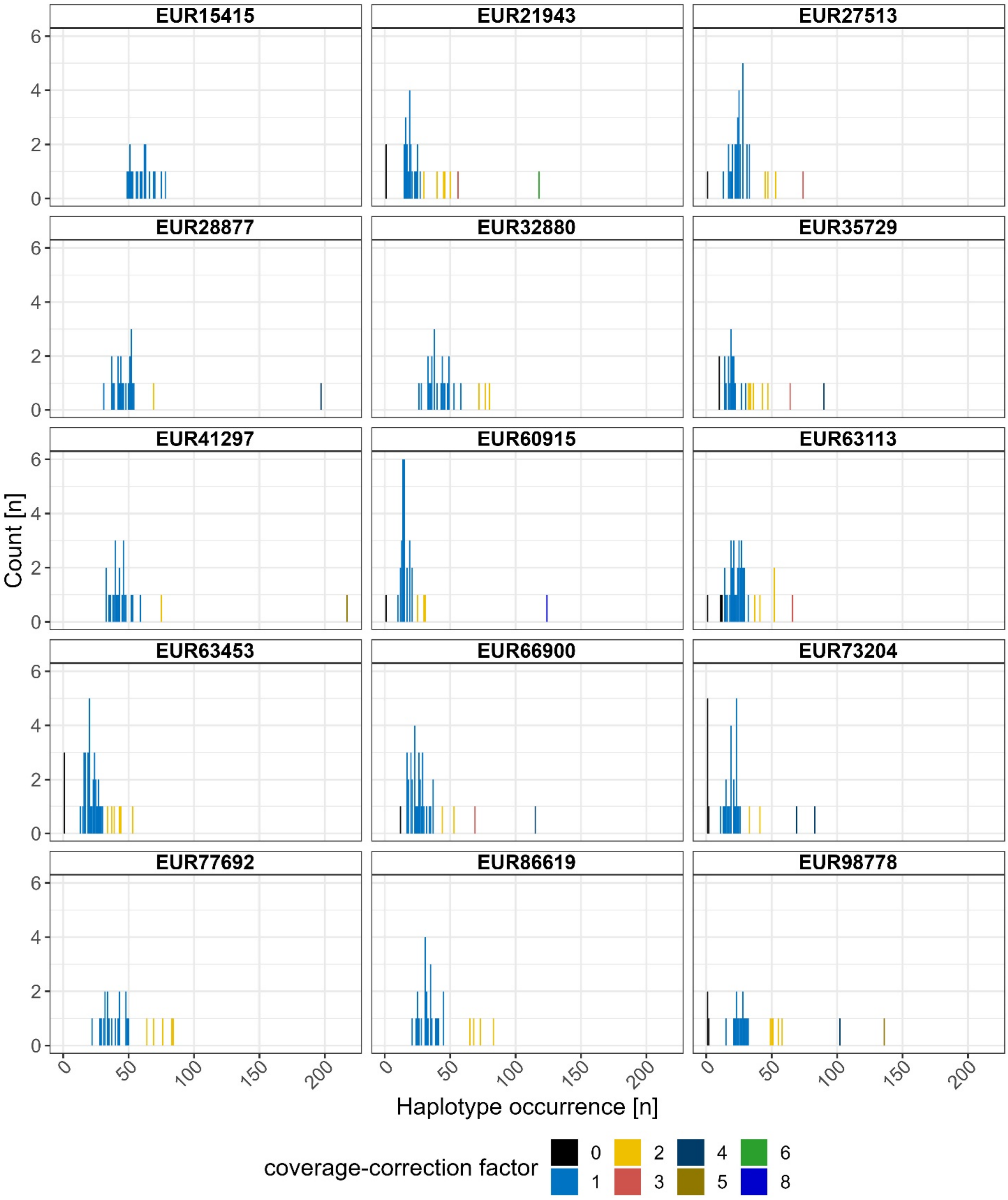

Fig S13: Occurrence of the different haplotypes for the 15 SAPHIR samples.

We observed haplotype occurrences that were not according to the assumed binomial distribution, suggesting the occurrence of identical KIV-2 repeats (e.g. recent repeat expansions). This was leveraged to determine a coverage-correction factor dependent on the occurrence of each haplotype per sample.

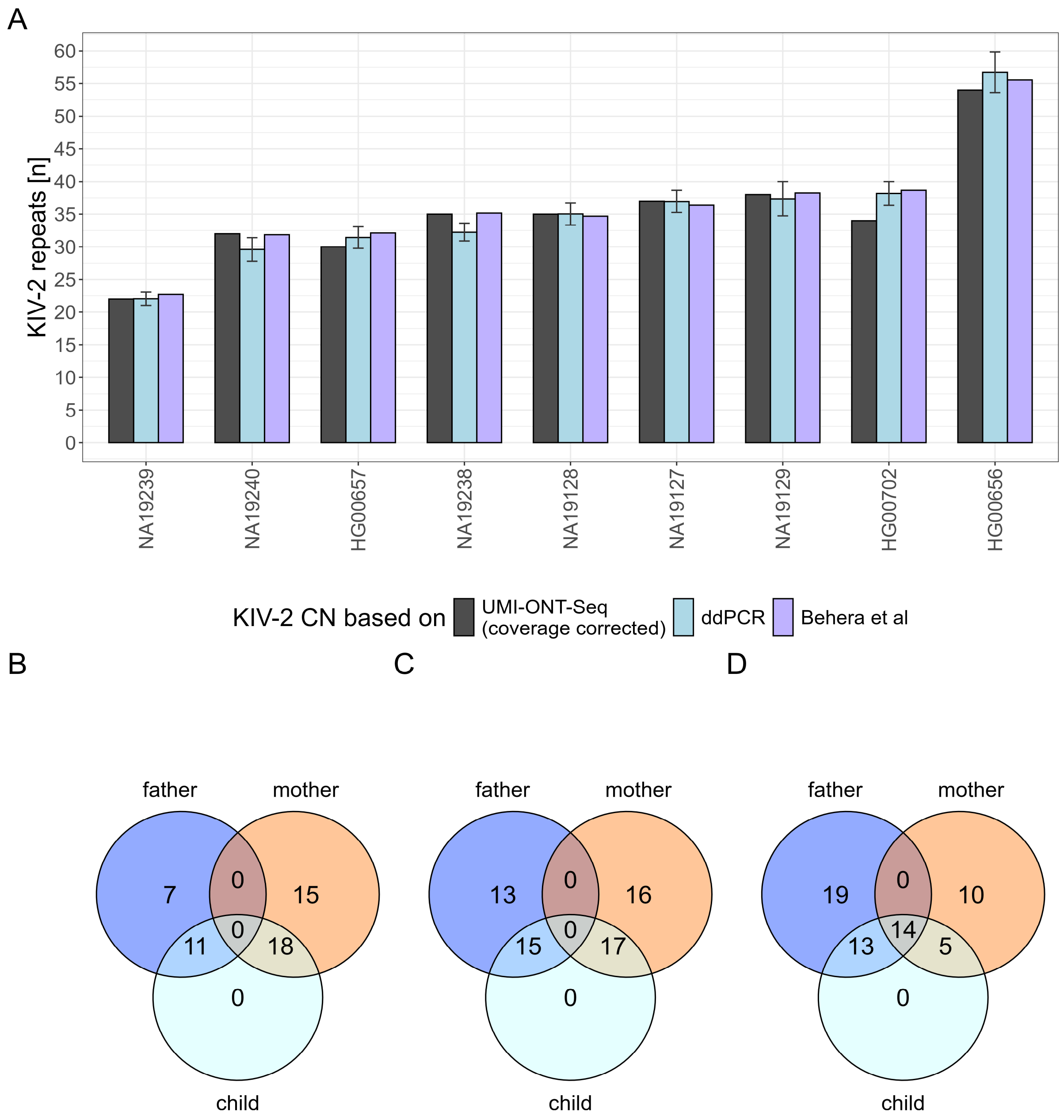

Fig S14: Correlation of UMI-ONT-Seq and ddPCR based number of KIV-2 repeats and Venn diagrams showing the haplotypes for 3 trios.

Panel A: The number of KIV-2 repeats per sample determined by coverage-corrected haplotypes from UMI-ONT-Seq (black), ddPCR (blue) and Behera et al, bioRxiv 2023.04.24.538128 (purple). UMI-ONT-Seq accurately reflects the number of KIV-2 repeats per sample determined by on ddPCR (r: 0.974) and Behera et al (r: 0.982). **Panel B-D:** Venn diagram showing the overlap of haplotypes for the three trios. All haplotypes found in the children are base-identical with the haplotypes that are found in the parents. **B:** Family 1: In the child (NA19240) 29 different haplotypes were found, of which 11 were paternal (NA19239) and 18 maternal (NA19238). **C:** Family 2: In the child (NA19129) 32 different haplotypes were found, of which 15 were paternal (NA19128) and 17 maternal (NA19128). **D:** Family 3: In the child (HG00702) 32 different haplotypes were found, of which 13 were paternal (HG00656), 5 maternal (HG00657) and 14 were found in all three individuals.

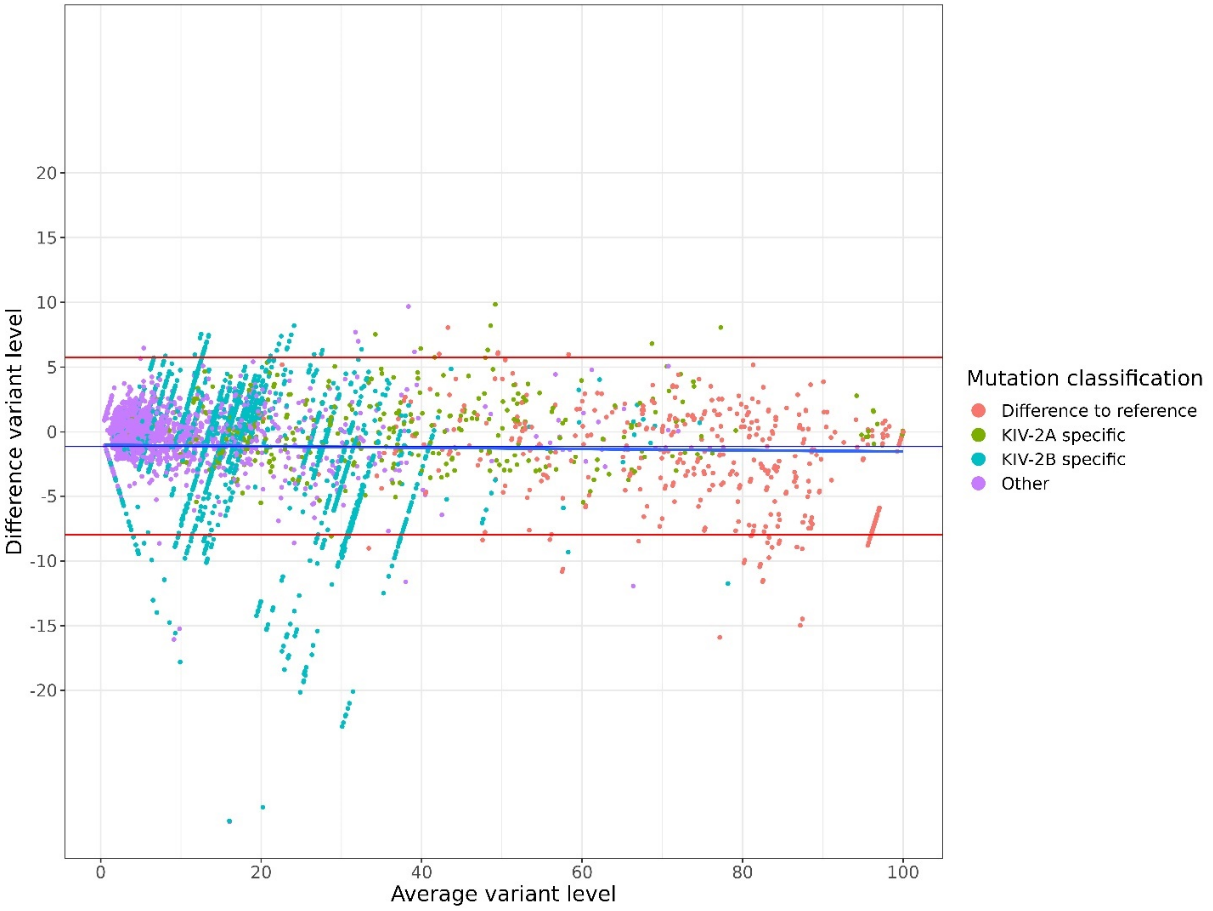

Fig S15: Bland Altman plot of the 48 1000G samples analyzed with UMI-ONT-Seq compared variant levels derived from publically available WGS data[11].

Mutations were classified according to their specificity for certain KIV-2 subtypes according to [1] (red: Difference to the reference sequence [No specific subtype], green: KIV-2A specific, blue: KIV-2B specific, purple: Not classified]. Variant levels were highly correlated, showed no bias, high accordance for all variant levels and low margins of error (< 5 %). A subset of KIV-2B specific mutations was found only with UMI-ONT-Seq and therefore showed higher deviation between both methods (turquoise points).

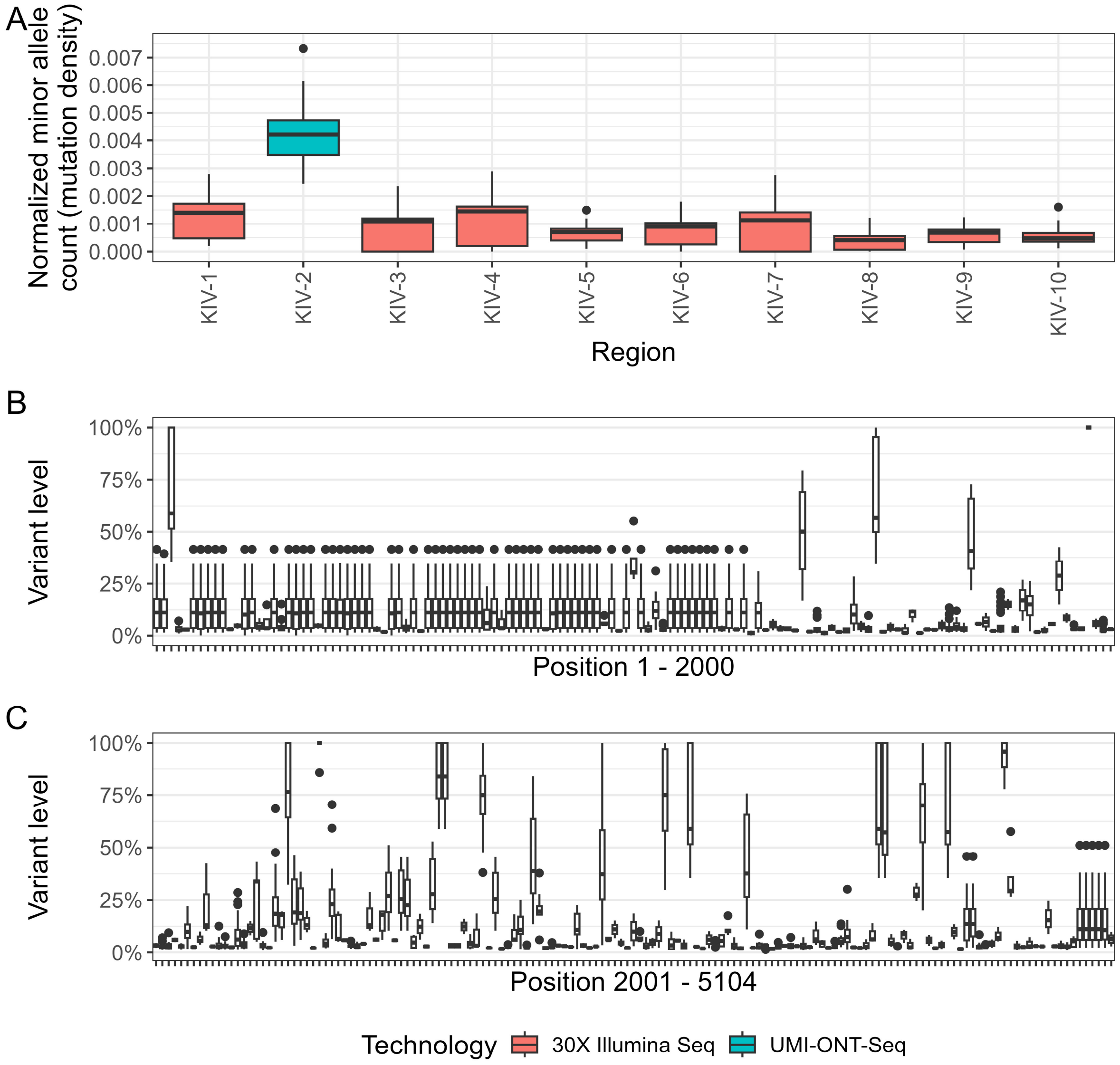

Fig S16: SNP density of the KIV domains and variant levels in the KIV-2.

Panel A: Normalized allele count of 48 samples of the 1000G project from for the non-repetitive KIV domains (red; 30X Illumina sequencing) and for the KIV-2 VNTR (blue; UMI-ONT-Seq data). The allele count was normalized to the length of each domain (all KIV domains) and for the KIV-2 VNTR also for the copy number according to ddPCR. Median normalized allele count is about 2-times higher in the KIV-2 VNTR than in the non-repetitive KIV domains. **Panel B and C:** Boxplots showing the variant levels per position of the PCR5104 fragment based on UMI-ONT-Seq for the 48 1000G samples. Some variants are present at high levels throughout the whole sample-set.

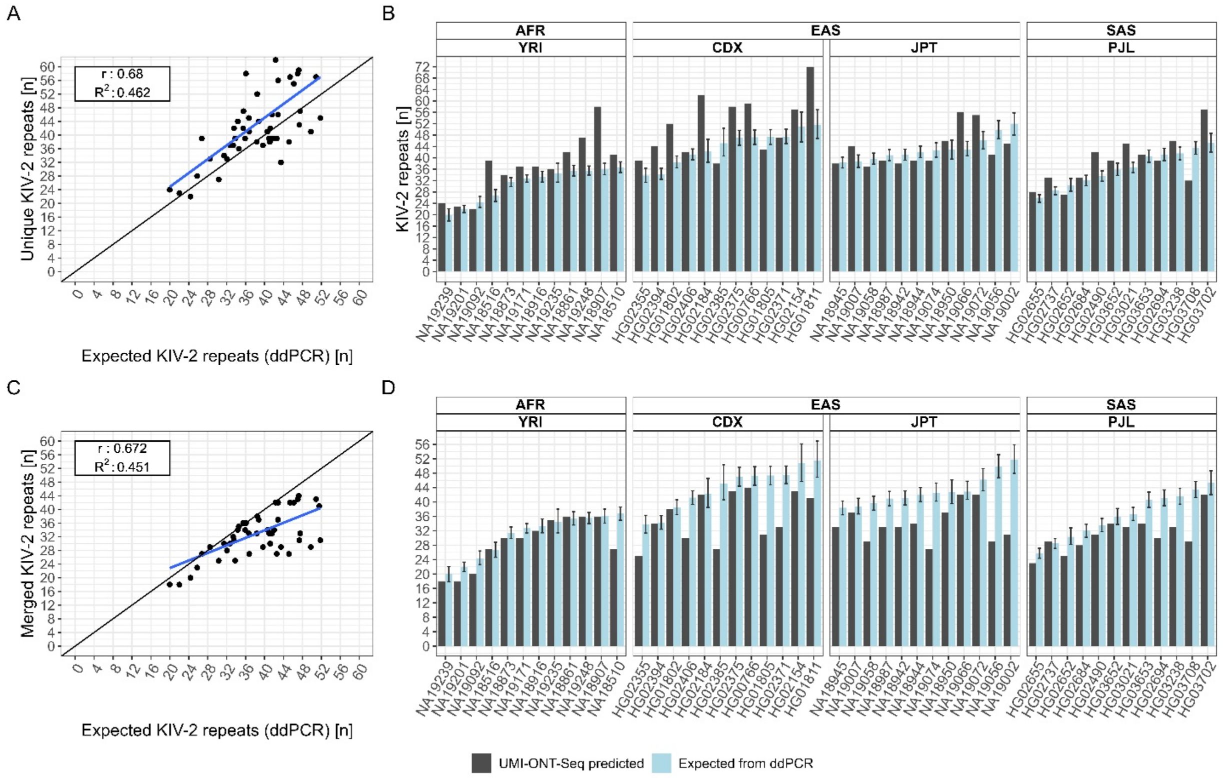

Fig S17: Correlation of expected (ddPCR) and UMI-ONT-Seq predicted number of KIV-2 for different steps of the haplotype extraction and number of KIV-2 repeat prediction in 1000G.

Panels A and B show the correlation and absolute concordance when only the uniquely occurring haplotypes are used for prediction. Taking only the unique haplotypes the number of KIV-2 repeats is generally overestimated and low correlation (r: 0.68, R^2: 0.462). **Panels C and D** show the correlation and absolute concordance after merging small haplotype clusters (occurrence < 0.85% of all consensus sequences) to cluster within a maximum of 1 edit distance and removing all small clusters that were not able to be merged. After that no sample showed more than the expected number of KIV-2 repeats and correlation increased to 0.732 (r) and 0.536 (R^2). For about one third of the samples the number of KIV-2 repeats determined by UMI-ONT-Seq is indeed within the 95% confidence interval of the ddPCR. This is improved further after coverage-correction (see Figure 6).

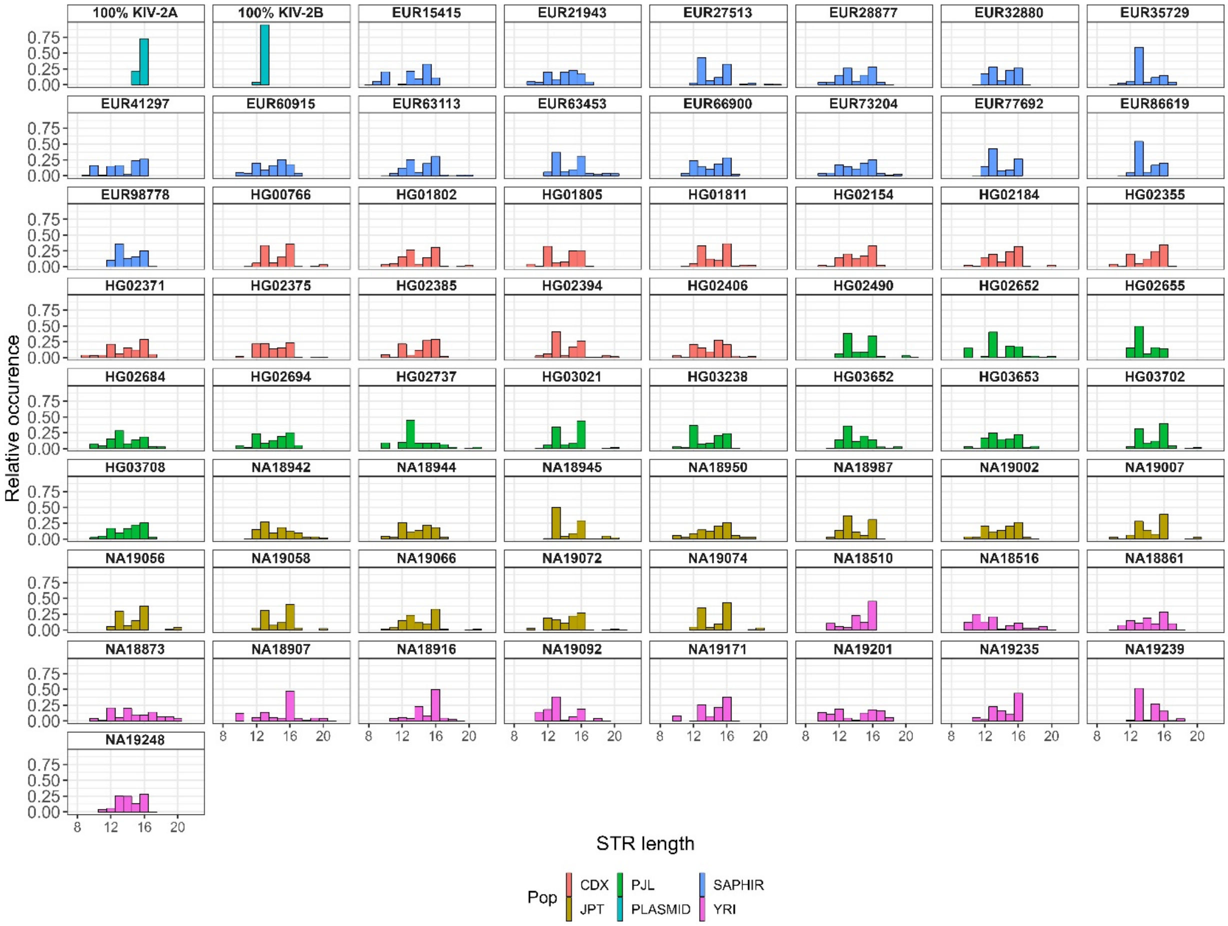

Fig S18: Length distribution (relative occurrence) of the extracted STR-region of 63 gDNA samples of 5 different populations (1000G and SAPHIR) and the two unmixed plasmids.

Using the high-quality UMI-ONT-Seq consensus sequences, we extracted 62,679 STR regions and counted the number of CA-repeats. The STR-region contains 7 to 22 repeats. While the STR-length was homogenous throughout all populations, a degeneration of the STR at position 7 and 15 suggest potential population specific pattern (Table S31 and Table S32).

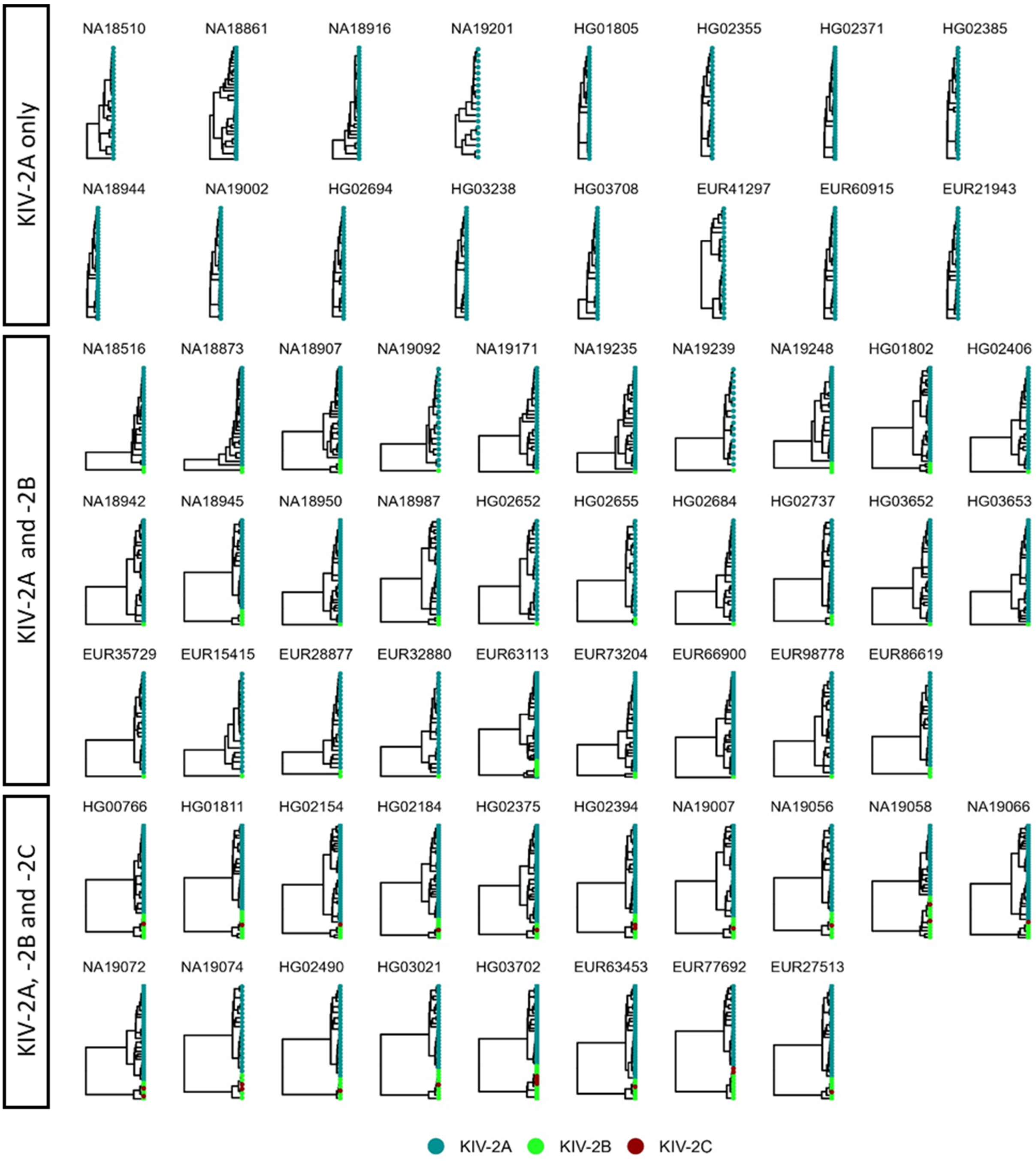

Fig S19: KIV-2 subtype specific haplotype diversity of all 63 human gDNA samples in the present study (1000G, SAPHIR).

KIV-2 subtype-specific haplotype diversity. Splitting the samples by their KIV-2 subtypes, revealed the 2 major clusters representing KIV-2A and the phylogenetically distant KIV-2B and C subtypes. While the KIV-2B and C cluster contain very similar sequences, with low diversity, the KIV-2A cluster has large internal differentiation. EUR: SAPHIR samples. Other samples: 1000G. See Table S27 for population assignment of the 1000G sample IDs.

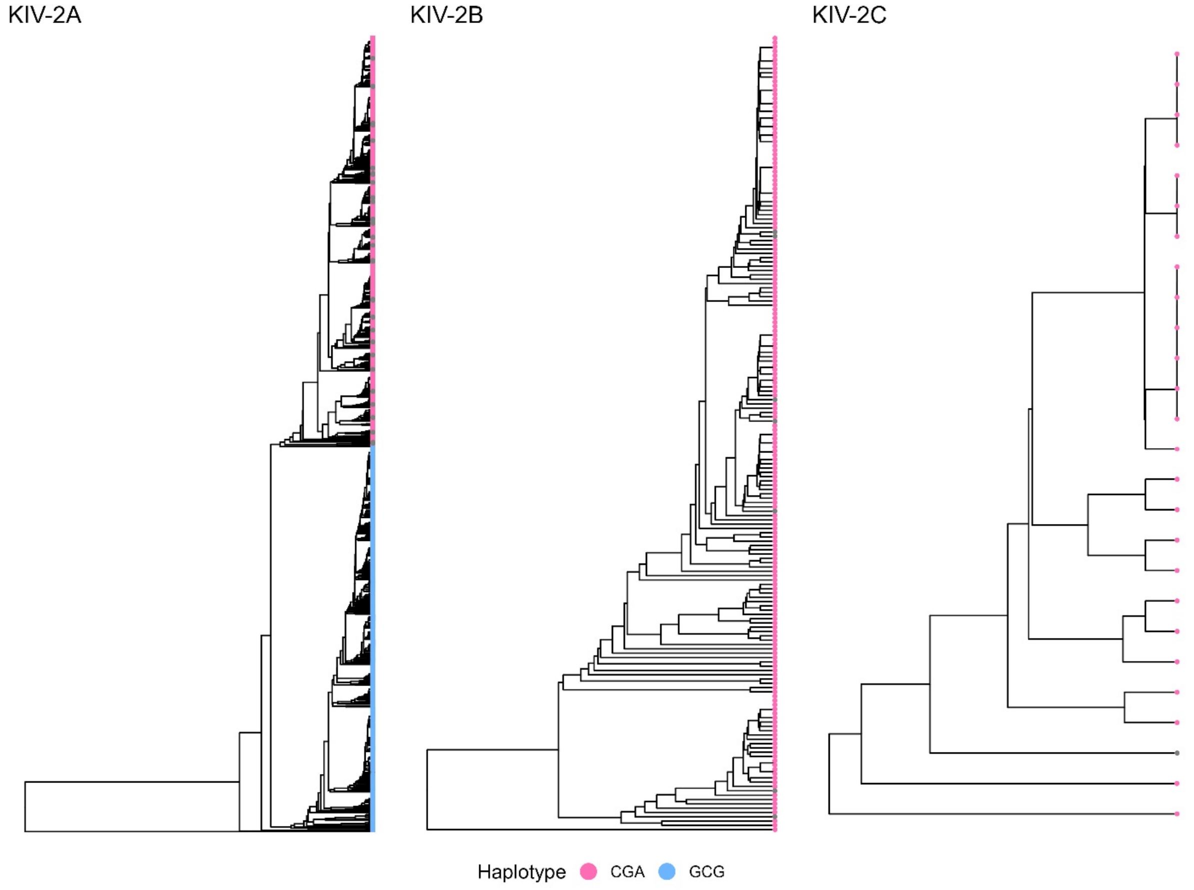

Fig S20: Classification of KIV-2A repeat subclusters using 3 positions (35, 3103, 4358) of a previously suggested haplotype in a multi-ancestry sample set (1000G and SAPHIR) of 63 samples.

Using three positions (35, 3103, 4358) of a previously suggested haplotype we were readily able to divide KIV-2A into the two main subclusters (CGA: pink or GCG: blue) observed in our analysis. All KIV-2B and KIV-2 repeats showed the same haplotype at these positions in all 63 samples.

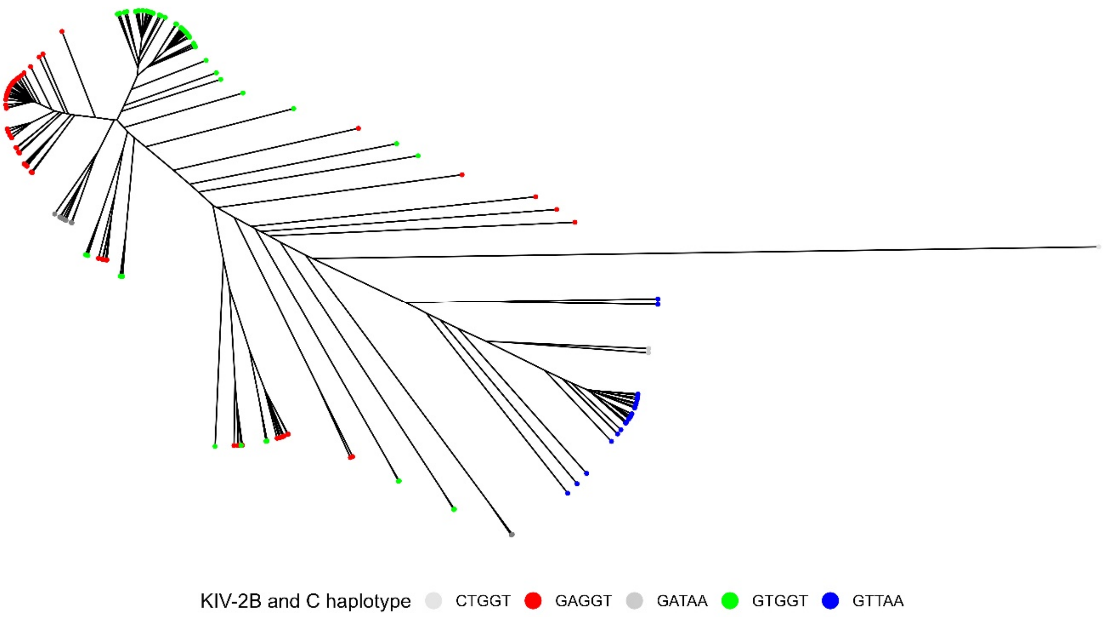

Fig S21: Classification of KIV-2B and KIV-2C repeats into three main subclusters using 5 positions (50, 2409, 5037, 5045, 5052) in a multi-ancestry sample set (1000G and SAPHIR) of 63 samples.

We observed 5 positions that split KIV-2B and KIV-2C repeats into three main subclusters. Interestingly, KIV-2C repeats were not found to build a distinct cluster. Noteworthy, the three haplotypes GAGGT, GTGGT and GTTAA represented 98.5 % of all found haplotypes.

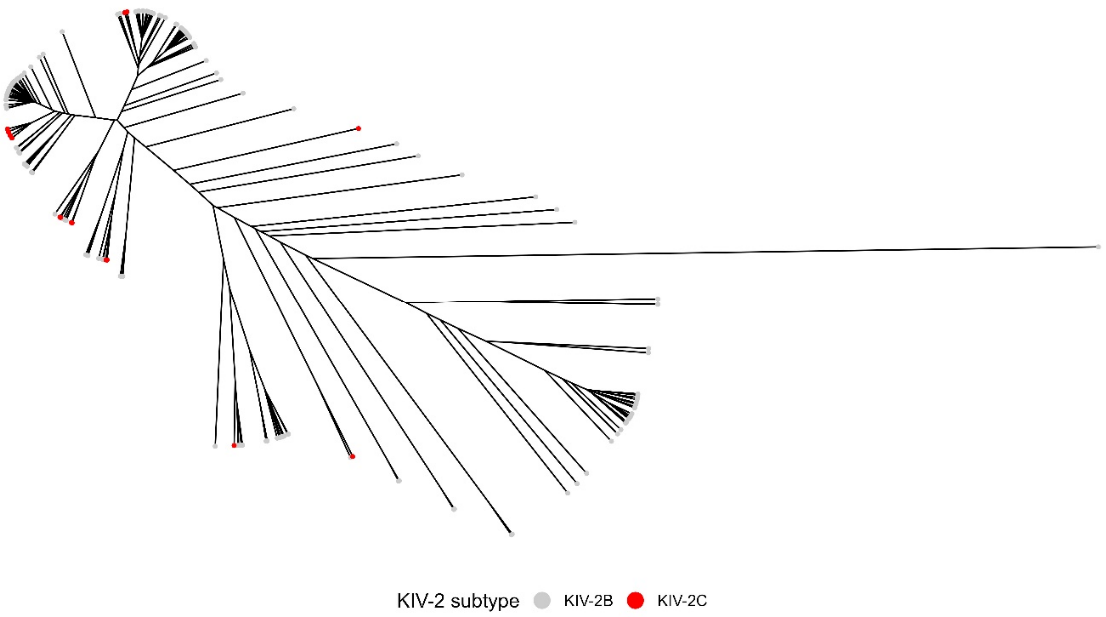

Fig S22: Classification of KIV-2C containing repeats with the commonly used KIV-2 subtype defining positions (594, 621, 666) in a multi-ancestry sample set (1000G and SAPHIR) of 63 samples.

Despite being associated with KIV-2B and KIV-2C, the three positions are not sufficient two define a distinct KIV-2C subcluster in all KIV-2B and KIV-2C containing repeats. KIV-2C can be found in several subclusters.

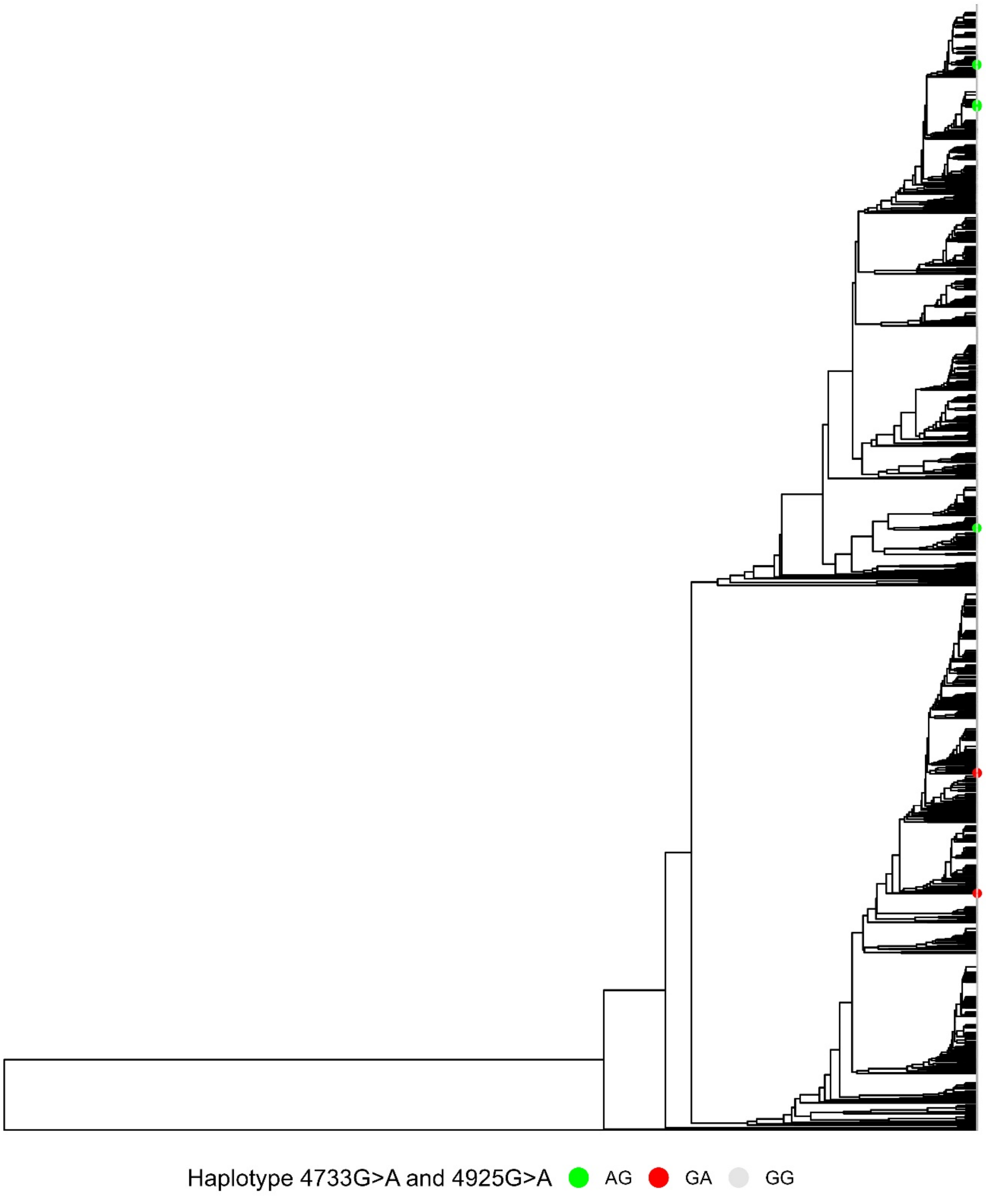

Fig S23: Classification of the two most frequent disease relevant variants in the KIV-2 VNTR (4925G>A: red and 4733G>A: green) in a multi-ancestry sample set (1000G and SAPHIR) of 63 samples.

The majority of the repeats was wild type for both alleles (GG: grey). 7 samples were found the carry either 4925G>A and 4733G>A. The two variants were solely found in KIV-2A repeats and associate with two different KIV-2A subclusters . Of 7 samples carrying either one of the two mutations 6 were European and 1 Punjabi. We observed 100% inter-individual sequence identity of the complete KIV-2 repeat in two (4925G>A) and three (4733G>A) samples.

Supplementary Tables

Table S1: ddPCR validation of the two unmixed and the plasmid mixtures for both fragments. Abundance is the fractional abundance of KIV-2B specific alleles in the plasmid mixtures. CI: Confidence interval

Sample	Fragment	Abundance [%]	Abundance CI 95% - Low	Abundance CI 95% - High
100 % KIV-2A	2645	99.75	99.56	99.94
100 % KIV-2B	2645	99.88	99.63	100.12
5%	2645	5.29	4.66	5.93
2.5%	2645	3.21	2.28	4.04
1.5%	2645	1.65	1.16	2.18
1%	2645	1.33	1.02	1.64
0.5%	2645	0.68	0.59	0.77
100 % KIV-2A	5104	99.14	98.69	99.58
100 % KIV-2B	5104	99.94	99.79	100.08
5%	5104	5.43	4.69	6.16
2.5%	5104	2.97	2.16	3.77
1.5%	5104	1.39	0.8	1.99
1%	5104	1.18	0.67	1.69
0.50%	5104	0.62	0.50	0.74

Table S2: ddPCR and qPCR reaction conditions. MGBEQ: minor groove binding black hole quencher. The ddPCR for KIV-2A/KIV-2B plasmid mixture validation uses two probes binding in *LPA* KIV-2 exon 1 at a base that differs between the two subtypes.

	**KIV-2 copy number ddPCR**	**KIV2B quantification ddPCR**
Product length [bp]	94	79
Reaction volume [µL]	20	20
Enzyme	Biorad ddPCR 2X Supermix for Probes (No dUTP)	Biorad ddPCR 2X Supermix for Probes (No dUTP)
Forward primer (5'-3') [a]	GTGGCAGCTCCTTATTGTTATACG	TTTTCATTTCAGCACCGACTGA
Reverse primer (5'-3') [a]	CGACGGCAGTCCCTTCTG	GTGCCTCGATAACTCTGTCCATT
Probe (5'-3') [a]	[FAM]-TACTGCAACCTGACGCAATGCTCAGAC-[BHQ1]	[HEX]-AGGAGTGCTACCACGG-[MGBEQ]<br>[FAM]-AGGAGTGCTACCATGGT-[MGBEQ]
Diploid reference assay	ThermoFisher RNAse P Copy Number Reference assay<br>(Part.no 4403328)	none
Final primer concentration [nM]	900 nM	900 nM
Final *LPA* probe concentration [nM]	300 nM	300 nM
Initial denaturation	95 °C, 10 min	95 °C, 10 min
Denaturation	94 °C, 30 s,	94 °C, 30 s
Annealing (ramp rate)	60 °C, 60 s (2 °C/s)	57.4 °C, 30 s (2 °C/s)
Extension step	None, 2 step PCR	60.0 °C, 30 sec
Cycles	40	40
Final denaturation	98°C, 10 min	98°C, 10 min
Sample input [ng]	4 ng EcoRI digested	4 ng Plasmid

Table S3: Primer sequences. The tagging primers have a universal amplification sequence, the UMI sequence and the KIV-2 5104 or 2645 fragment-specific primer sequence. The amplification primer are complementary to the universal primer sequence of the tagging primer. Bold: locus-specific primer sequences from references [1, 7, 12]. Underlined Universal priming site (UVP). UMI protocol-specific primer design is from ONT technical note CPU_9107_v109_revC_09Oct2020.

ID	Target	Orientation	Step	Sequence (5‘-3‘)
1	PCR2645	forward	tagging	GTATCGTGTAGAGACTGCGTAGGTTTVVVVTTVVVVTTVVVVTTVVVVTTT**AGAAACAAACCTACTAAACCTGACAG**
2	PCR2645	reverse	tagging	AGTGATCGAGTCAGTGCGAGTGTTTVVVVTTVVVVTTVVVVTTVVVVTTT**TTTTTCTGACAATCGGAATATAC**
3	PCR5104	forward	tagging	GTATCGTGTAGAGACTGCGTAGGTTTVVVVTTVVVVTTVVVVTTVVVVTTT**TCAGGATGCAGGGCATGAG**
4	PCR5104	reverse	tagging	AGTGATCGAGTCAGTGCGAGTGTTTVVVVTTVVVVTTVVVVTTVVVVTTT**CACCAGAAATCACTCCGCTG**
5	UVP für both targets	forward	amplification	GGTGCTGAAGAAAGTTGTCGGTGTCTTTGTGTTAACCGTATCGTGTAGAGACTGCGTAGG
6	UVP für both targets	reverse	amplification	GGTGCTGAAGAAAGTTGTCGGTGTCTTTGTGTTAACCAGTGATCGAGTCAGTGCGAGTG

Table S4: PCR conditions for the 2645 and the 5104 fragment.

	PCR2645 - tagging	PCR2645 - early UVP PCR	PCR2645 - late UVP PCR	PCR5104 - tagging	PCR5104 - early UVP PCR	PCR5104 - late UVP PCR
Product length [bp]	2746	2820	2820	5205	5279	5279
Reaction Volume [µL]	15	50	50	15	50	50
Enzyme	Superfi II [a]	Superfi II [a]	Superfi II [a]	Superfi II [a]	Superfi II [a]	Superfi II [a]
Initial Denaturation	98 °C, 180 s,	98 °C, 180 s,	98 °C, 180 s,	98 °C, 180 s,	98 °C, 180 s,	98 °C, 180 s,
Denaturation	98 °C, 30 s,	98 °C, 20 s,	98 °C, 20 s,	98 °C, 30 s,	98 °C, 20 s,	98 °C, 20 s,
Annealing, ramp rate	66 - 60 °C, 90 s, 0.2 °C/s	1 s 70°C, 70 - 63 °C, 45 s, 0.4 °C/s 1 s 63°C	-	66 - 60 °C, 90 s, 0.2 °C/s	1 s 70°C, 70 - 63 °C, 45 s, 0.4 °C/s 1 s 63°C	-
Extension	72 °C, 90 s,	72 °C, 90 s,	72 °C, 120 s,	72 °C, 180 s,	72 °C, 180 s,	72 °C, 240 s,
Cycles	2	5	28	2	5	28
Denaturation 2	-	98 °C, 20 s,	-	-	98 °C, 20 s,	-
Extension 2	-	72 °C, 120 s,	-	-	72 °C, 180 s,	-
Cycles	1	5		1	5	
Final extension	72 °C, 300 s,	72 °C, 300 s,	72 °C, 300 s,	72 °C, 300 s,	72 °C, 300 s,	72 °C, 300 s,
Primer	1, 2 [b]	3, 4 [b]	3, 4 [b]	5, 6 [b]	7, 8 [b]	7, 8 [b]
Final primer conc. [nM]	750	830	830	750	830	830
Final MgCl2 conc. [mM]	-	3.3	3.3	-	3.3	3.3
Sample input	2 ng	21 µl	21 µl	2 ng	21 µl	21 µl
Targets	50,000	-	-	50,000	-	-

[a] Full name of the enzyme: ThermoFisher Scientific Platinum SuperFi II Green PCR Master Mix.

[b] PrimerIDs refer to Table S3.

Table S5: Summary statistics of the sequencing results per kit and basecalling algorithm.

Kit	Algorithm	Number of reads	Median Q-score	Mean Q-score	Number of bases [Gb]	Number of samples
R9	HAC	3509445	13.2	13.1	14.7	29
V14	HAC	3227736	15.7	15.3	13.8	29
V14	SUP	3548652	18.5	17.9	14	29
V14	SUPDUP	277911	28.6	28	1.4	12
Total	-	10563744	-	-	43.9	99

Table S6: Sequencing overview R9 HAC.

Sample	Fragment	Number of reads	Number of bases [Mb]	Median Q-score	Mean Q-score	Median read length [b]	Mean read length [b]
100% KIV-2B	2645	109843	303.5	12.1	12.1	2911	2762.7
5%	2645	126532	359.7	12	12.1	2911	2842.6
2.50%	2645	113217	323.4	12.4	12.4	2913	2856.1
1.50%	2645	93180	264.6	12.4	12.3	2913	2839.2
1%	2645	101180	288	12.4	12.3	2912	2846.4
0.50%	2645	137340	388.5	12	12.1	2912	2828.5
100% KIV-2A	2645	134277	383.1	12	12.1	2913	2853.3
100% KIV-2B	5104	125709	639.7	13.8	13.6	5335	5088.9
5%	5104	127595	602.2	12.7	12.6	5283	4719.3
2.50%	5104	77479	397	12.1	12.1	5333	5123.4
1.50%	5104	106608	535.4	13.8	13.5	5327	5022.3
1%	5104	105730	497.6	12.7	12.6	5281	4705.9
0.50%	5104	152135	770	13.8	13.5	5336	5061
100% KIV-2A	5104	163591	819.8	13.8	13.5	5339	5011.3
EUR15415	5104	70038	317.1	14	13.7	5308	4527.4
EUR21943	5104	100754	492	13.4	13.2	5329	4883
EUR27513	5104	88489	389.9	13.9	13.7	5321	4406.6
EUR28877	5104	156027	771.2	13.5	13.3	5342	4943
EUR32880	5104	151916	739.8	13.5	13.3	5339	4869.8
EUR35729	5104	95958	469.7	13.5	13.3	5328	4895
EUR41297	5104	128104	628.8	13.5	13.3	5336	4908.3
EUR60915	5104	110100	529.6	13.5	13.2	5329	4810.1
EUR63113	5104	157136	689.4	14	13.7	5331	4387.3
EUR63453	5104	114891	518.8	13.5	13.3	5323	4516
EUR66900	5104	93850	397	13.9	13.7	5313	4229.7
EUR73204	5104	208536	494.3	13.8	13.6	1500	2370.2
EUR77692	5104	147495	640.1	13.9	13.7	5326	4339.7
EUR86619	5104	97587	485.7	13.9	13.7	5343	4977.6
EUR98778	5104	114148	560.6	13.9	13.7	5343	4911

Table S7: Sequencing overview V14 HAC.

Sample	Fragment	Number of reads	Number of bases [Mb]	Median Q-score	Mean Q-score	Median read length [b]	Mean read length [b]
100% KIV-2B	2645	105403	307.2	14.8	14.5	2940	2914.9
5%	2645	110703	304.1	18.9	18.4	2817	2746.6
2.50%	2645	105147	289.5	18.9	18.4	2817	2753
1.50%	2645	94187	261.1	18.9	18.3	2817	2772.2
1%	2645	131673	368.2	14.9	14.5	2937	2796.7
0.50%	2645	91836	243.5	18.9	18.4	2816	2652
100% KIV-2A	2645	96155	261.1	18.8	18.3	2816	2715.3
100% KIV-2B	5104	145038	576.7	14.9	14.6	5363	3976.5
5%	5104	107300	533.4	15.3	14.9	5385	4970.8
2.50%	5104	127751	636.9	15.4	14.9	5385	4985.1
1.50%	5104	110409	528.8	15	14.6	5381	4789.9
1%	5104	113702	568.5	15.4	15	5386	5000.3
0.50%	5104	94354	485.7	15.4	15	5387	5148.1
100% KIV-2A	5104	104810	513.1	15.3	14.9	5384	4895.2
EUR15415	5104	107148	530.3	15	14.6	5378	4949.6
EUR21943	5104	115224	572.5	15	14.7	5380	4968.4
EUR27513	5104	90414	415.1	14.9	14.6	5375	4591.1
EUR28877	5104	106609	532.9	15.1	14.7	5382	4998.3
EUR32880	5104	103952	515.9	15.1	14.7	5381	4962.6
EUR35729	5104	100376	500.7	15.1	14.7	5381	4987.8
EUR41297	5104	114752	573.3	15.1	14.7	5381	4996.1
EUR60915	5104	101977	503.7	15	14.6	5380	4939.2
EUR63113	5104	100513	457.5	15	14.6	5376	4551.5
EUR63453	5104	99587	470.5	15	14.6	5380	4724.2
EUR66900	5104	112271	501.4	15	14.7	5374	4465.6
EUR73204	5104	157966	441.8	14.5	14.3	1525	2796.7
EUR77692	5104	102688	467.9	14.9	14.6	5376	4556.9
EUR86619	5104	116328	589.5	15	14.6	5381	5067.6
EUR98778	5104	159463	803.3	15	14.7	5381	5037.3

Table S8: Sequencing overview V14 SUP.

Sample	Fragment	Number of reads	Number of bases [Mb]	Median Q-score	Mean Q-score	Median read length [b]	Mean read length [b]
100% KIV-2B	2645	110348	305.2	18.2	17.7	2819	2766.2
5%	2645	110703	304.1	18.9	18.4	2817	2746.6
2.50%	2645	105147	289.5	18.9	18.4	2817	2753
1.50%	2645	94187	261.1	18.9	18.3	2817	2772.2
1%	2645	133275	359.5	18.5	18	2816	2697.5
0.50%	2645	91836	243.5	18.9	18.4	2816	2652
100% KIV-2A	2645	96155	261.1	18.8	18.3	2816	2715.3
100% KIV-2B	5104	179155	610.7	18.4	17.8	4788	3408.7
5%	5104	109517	531.2	18.8	18.1	5268	4850.2
2.50%	5104	130071	632.5	18.8	18.1	5267	4862.9
1.50%	5104	146157	569.6	18.2	17.7	5256	3896.9
1%	5104	115813	565.1	18.8	18.2	5267	4879.6
0.50%	5104	96259	484.2	18.9	18.3	5269	5030
100% KIV-2A	5104	106702	509.7	18.8	18.2	5267	4776.4
EUR15415	5104	109926	532.2	18.4	17.7	5263	4841.9
EUR21943	5104	117770	572.8	18.3	17.7	5263	4863.5
EUR27513	5104	106423	432.8	18.1	17.5	5256	4067
EUR28877	5104	108763	532.7	18.4	17.8	5264	4897.5
EUR32880	5104	106256	516.1	18.4	17.8	5264	4857.5
EUR35729	5104	102554	501	18.4	17.7	5264	4885.3
EUR41297	5104	117428	574.3	18.5	17.8	5264	4890.7
EUR60915	5104	104458	504.7	18.4	17.7	5263	4832.1
EUR63113	5104	119810	478	18.3	17.6	5255	3989.6
EUR63453	5104	101851	471.3	18.4	17.7	5264	4627.8
EUR66900	5104	138525	528.8	18.3	17.7	5249	3817
EUR73204	5104	261885	556.5	18.2	17.7	1389	2124.8
EUR77692	5104	124413	491.4	18.2	17.5	5253	3949.7
EUR86619	5104	125810	599	18.2	17.5	5262	4760.8
EUR98778	5104	177455	822.6	18.3	17.6	5262	4635.6

Table S9: Sequencing overview V14 SUPDUP.

Sample	Fragment	Number of reads	Number of bases [Mb]	Median Q-score	Mean Q-score	Median read length [b]	Mean read length [b]
EUR15415	5104	29849	155.9	28.7	28.1	5273	5223
EUR21943	5104	29657	154.8	29.1	28.4	5272	5218.5
EUR28877	5104	30581	160.3	29	28.3	5273	5242.2
EUR32880	5104	28577	149.2	28.9	28.2	5273	5219.7
EUR35729	5104	24606	128.6	28.8	28.1	5273	5228.3
EUR41297	5104	34236	179.2	29	28.3	5273	5234.4
EUR60915	5104	26888	140.2	29.1	28.5	5272	5214.1
EUR63113	5104	11125	49.7	28.4	28	5270	4463.5
EUR63453	5104	25678	133	28.2	27.5	5275	5180.4
EUR73204	5104	6685	8.1	25.9	25.8	1158	1206.9
EUR86619	5104	12618	64.3	28.9	28.3	5271	5097.3
EUR98778	5104	17411	87.4	28.9	28.4	5271	5019.7

Table S10: Performance measures for all plasmid mixtures analyzed with the initial version of the pipeline.

Sample	Fragment	Kit	Algorithm	Raw reads	Consensus sequences	True positive	True negative	False positive	False negative	Sensitivity	Specificity	Precision	F1 score
100 % KIV-2A	2645	R9	HAC	134277	600	8	2637	0	0	1	1	1	1
100 % KIV-2B	2645	R9	HAC	109843	495	112	2533	0	0	1	1	1	1
5%	2645	R9	HAC	103124	578	118	2527	0	0	1	1	1	1
2.50%	2645	R9	HAC	113217	618	118	2527	0	0	1	1	1	1
1.50%	2645	R9	HAC	93180	390	117	2527	0	1	0.992	1	1	0.996
1%	2645	R9	HAC	101180	507	118	2527	0	0	1	1	1	1
0.50%	2645	R9	HAC	137340	611	68	2527	0	50	0.576	1	1	0.731
100 % KIV-2A	5104	R9	HAC	163591	636	27	5077	0	0	1	1	1	1
100 % KIV-2B	5104	R9	HAC	125709	576	75	5029	0	0	1	1	1	1
5%	5104	R9	HAC	127595	496	88	5014	2	0	1	1	0.978	0.989
2.50%	5104	R9	HAC	77479	416	88	5015	1	0	1	1	0.989	0.994
1.50%	5104	R9	HAC	106608	461	88	5015	1	0	1	1	0.989	0.994
1%	5104	R9	HAC	105730	493	27	5015	1	61	0.307	1	0.964	0.466
0.50%	5104	R9	HAC	152135	555	27	5015	1	61	0.307	1	0.964	0.466
100 % KIV-2A	2645	V14	HAC	96155	628	8	2637	0	0	1	1	1	1
100 % KIV-2B	2645	V14	HAC	105403	310	112	2533	0	0	1	1	1	1
5%	2645	V14	HAC	110703	708	118	2527	0	0	1	1	1	1
2.50%	2645	V14	HAC	105147	818	118	2527	0	0	1	1	1	1
1.50%	2645	V14	HAC	94187	463	118	2527	0	0	1	1	1	1
1%	2645	V14	HAC	131673	387	118	2527	0	0	1	1	1	1
0.50%	2645	V14	HAC	91836	283	118	2526	1	0	1	1	0.992	0.996
100 % KIV-2A	5104	V14	HAC	81945	417	27	5077	0	0	1	1	1	1
100 % KIV-2B	5104	V14	HAC	145038	779	75	5029	0	0	1	1	1	1
5%	5104	V14	HAC	107300	435	88	5016	0	0	1	1	1	1
2.50%	5104	V14	HAC	127751	833	88	5015	1	0	1	1	0.989	0.994
1.50%	5104	V14	HAC	110409	659	88	5016	0	0	1	1	1	1
1%	5104	V14	HAC	113702	224	87	5013	3	1	0.989	0.999	0.967	0.978
0.50%	5104	V14	HAC	94354	343	27	5016	0	61	0.307	1	1	0.47
100 % KIV-2A	2645	V14	SUP	96155	627	8	2637	0	0	1	1	1	1

100 % KIV-2B	2645	V14	SUP	110348	348	112	2533	0	0	1	1	1	1
5%	2645	V14	SUP	110703	725	118	2527	0	0	1	1	1	1
2.50%	2645	V14	SUP	105147	828	118	2527	0	0	1	1	1	1
1.50%	2645	V14	SUP	94187	462	118	2527	0	0	1	1	1	1
1%	2645	V14	SUP	133275	399	118	2527	0	0	1	1	1	1
0.50%	2645	V14	SUP	91836	289	118	2526	1	0	1	1	0.992	0.996
100 % KIV-2A	5104	V14	SUP	83509	395	27	5077	0	0	1	1	1	1
100 % KIV-2B	5104	V14	SUP	179155	816	75	5029	0	0	1	1	1	1
5%	5104	V14	SUP	109517	451	88	5016	0	0	1	1	1	1
2.50%	5104	V14	SUP	130071	944	88	5016	0	0	1	1	1	1
1.50%	5104	V14	SUP	146157	732	88	5016	0	0	1	1	1	1
1%	5104	V14	SUP	115813	215	87	5013	3	1	0.989	0.999	0.967	0.978
0.50%	5104	V14	SUP	96259	346	28	5016	0	60	0.318	1	1	0.483

Table S11: Edit distances within UMI sequences in the same UMI cluster for the original ONT pipeline.

Fragment	Kit	Algorithm	Clusters	Max UMI edit distance in clusters	Mean UMI edit distance in clusters	SD UMI edit distance in clusters
PCR2645	R9	HAC	28127	26	15.879	3.944
PCR2645	V14	HAC	15535	25	15.453	4.145
PCR2645	V14	SUP	14700	25	15.441	4.172
PCR5104	R9	HAC	46503	26	16.106	3.987
PCR5104	V14	HAC	35965	26	15.41	4.189
PCR5104	V14	SUP	32974	25	15.503	4.204

Table S12: Variant levels and noise of variant level using UMI-ONT-Seq with the default clustering strategy.

Sample	Fragment	Kit	Algorithm	Mean variant level [%]	SD variant level [%]	Absolute variant level range observed
100 % KIV-2B	PCR2645	R9	HAC	99.763	1.127	6.869
5%	PCR2645	R9	HAC	7.8	0.291	1.73
2.50%	PCR2645	R9	HAC	3.41	0.269	0.971
1.50%	PCR2645	R9	HAC	1.205	0.137	1.026
1%	PCR2645	R9	HAC	1.637	0.168	0.986
0.50%	PCR2645	R9	HAC	0.873	0.132	0.327
100 % KIV-2B	PCR5104	R9	HAC	100	0	0
5%	PCR5104	R9	HAC	3.421	0.23	0.806
2.50%	PCR5104	R9	HAC	1.446	0.031	0.24
1.50%	PCR5104	R9	HAC	0.871	0.028	0.217
1%	PCR5104	R9	HAC	0.609	0	0
0.50%	PCR5104	R9	HAC	0.18	0	0
100 % KIV-2B	PCR2645	V14	HAC	99.645	0.315	0.968
5%	PCR2645	V14	HAC	4.756	0.277	1.412
2.50%	PCR2645	V14	HAC	2.683	0.162	0.978
1.50%	PCR2645	V14	HAC	2.289	0.106	0.216
1%	PCR2645	V14	HAC	2.424	0.175	0.775
0.50%	PCR2645	V14	HAC	1.502	0.151	0.355
100 % KIV-2B	PCR5104	V14	HAC	99.996	0.023	0.128
5%	PCR5104	V14	HAC	4.116	0.107	0.682
2.50%	PCR5104	V14	HAC	1.624	0.068	0.24
1.50%	PCR5104	V14	HAC	1.291	0.228	0.759
1%	PCR5104	V14	HAC	0.89	0.057	0.45
0.50%	PCR5104	V14	HAC	0.292	0	0
100 % KIV-2B	PCR2645	V14	SUP	99.83	0.147	0.575
5%	PCR2645	V14	SUP	3.582	0.298	1.517
2.50%	PCR2645	V14	SUP	1.994	0.155	0.725
1.50%	PCR2645	V14	SUP	2.519	0.113	0.433
1%	PCR2645	V14	SUP	2.424	0.17	0.752

0.50%	PCR2645	V14	SUP	1.387	0.033	0.346
100 % KIV-2B	PCR5104	V14	SUP	99.996	0.022	0.123
5%	PCR5104	V14	SUP	2.89	0.185	0.443
2.50%	PCR5104	V14	SUP	1.665	0.221	0.847
1.50%	PCR5104	V14	SUP	1.505	0.035	0.274
1%	PCR5104	V14	SUP	1.372	0.132	0.93
0.50%	PCR5104	V14	SUP	0.578	0.053	0.578

Table S13: Performance measures for all plasmid mixtures analyzed with by UMI-ONT-Seq with the cluster splitting strategy.

Sample	Fragment	Kit	Algorithm	Raw reads	Consensus sequences	True positive	True negative	False positive	False negative	Sensitivity	Specificity	Precision	F1 score
100 % KIV-2A	PCR2645	R9	HAC	134277	265	8	2637	0	0	1	1	1	1
100 % KIV-2B	PCR2645	R9	HAC	109843	106	112	2533	0	0	1	1	1	1
5%	PCR2645	R9	HAC	126532	321	118	2522	5	0	1	0.998	0.959	0.979
2.50%	PCR2645	R9	HAC	113217	247	118	2526	1	0	1	1	0.992	0.996
1.50%	PCR2645	R9	HAC	93180	71	114	2504	27	0	0.966	0.991	0.809	0.88
1%	PCR2645	R9	HAC	101180	128	118	2520	7	0	1	0.997	0.944	0.971
0.50%	PCR2645	R9	HAC	137340	249	118	2524	3	0	1	0.999	0.975	0.987
100 % KIV-2A	PCR5104	R9	HAC	163591	613	27	5077	0	0	1	1	1	1
100 % KIV-2B	PCR5104	R9	HAC	125709	514	75	5029	0	0	1	1	1	1
5%	PCR5104	R9	HAC	127595	453	88	5008	8	0	1	0.998	0.917	0.957
2.50%	PCR5104	R9	HAC	77479	211	88	5002	14	0	1	0.997	0.863	0.926
1.50%	PCR5104	R9	HAC	106608	471	88	5015	1	0	1	1	0.989	0.994
1%	PCR5104	R9	HAC	105730	347	88	5004	12	0	1	0.998	0.88	0.936
0.50%	PCR5104	R9	HAC	152135	533	27	5016	0	61	0.307	1	1	0.47
100 % KIV-2A	PCR2645	V14	HAC	96155	760	8	2637	0	0	1	1	1	1
100 % KIV-2B	PCR2645	V14	HAC	105403	341	112	2533	0	0	1	1	1	1
5%	PCR2645	V14	HAC	110703	898	118	2527	0	0	1	1	1	1
2.50%	PCR2645	V14	HAC	105147	1047	118	2527	0	0	1	1	1	1
1.50%	PCR2645	V14	HAC	94187	548	118	2527	0	0	1	1	1	1
1%	PCR2645	V14	HAC	131673	432	118	2527	0	0	1	1	1	1
0.50%	PCR2645	V14	HAC	91836	282	118	2526	1	0	1	1	0.992	0.996
100 % KIV-2A	PCR5104	V14	HAC	104810	140	27	5077	0	0	1	1	1	1
100 % KIV-2B	PCR5104	V14	HAC	145038	1051	75	5029	0	0	1	1	1	1
5%	PCR5104	V14	HAC	107300	441	88	5016	0	0	1	1	1	1
2.50%	PCR5104	V14	HAC	127751	893	88	5016	0	0	1	1	1	1
1.50%	PCR5104	V14	HAC	110409	793	88	5016	0	0	1	1	1	1
1%	PCR5104	V14	HAC	113702	202	88	5015	1	0	1	1	0.989	0.994
0.50%	PCR5104	V14	HAC	94354	322	27	5016	1	60	0.307	1	0.964	0.466
100 % KIV-2A	PCR2645	V14	SUP	96155	746	8	2637	0	0	1	1	1	1

100 % KIV-2B	PCR2645	V14	SUP	110348	346	112	2533	0	0	1	1	1	1
5%	PCR2645	V14	SUP	110703	893	118	2527	0	0	1	1	1	1
2.50%	PCR2645	V14	SUP	105147	1010	118	2527	0	0	1	1	1	1
1.50%	PCR2645	V14	SUP	94187	541	118	2527	0	0	1	1	1	1
1%	PCR2645	V14	SUP	133275	436	118	2527	0	0	1	1	1	1
0.50%	PCR2645	V14	SUP	91836	278	118	2526	1	0	1	1	0.992	0.996
100 % KIV-2A	PCR5104	V14	SUP	106702	185	27	5077	0	0	1	1	1	1
100 % KIV-2B	PCR5104	V14	SUP	179155	1206	75	5029	0	0	1	1	1	1
5%	PCR5104	V14	SUP	109517	462	88	5016	0	0	1	1	1	1
2.50%	PCR5104	V14	SUP	130071	1100	88	5016	0	0	1	1	1	1
1.50%	PCR5104	V14	SUP	146157	932	88	5016	0	0	1	1	1	1
1%	PCR5104	V14	SUP	115813	222	88	5015	1	0	1	1	0.989	0.994
0.50%	PCR5104	V14	SUP	96259	332	27	5016	0	61	0.307	1	1	0.47

Table S14: Noise reduction of detected variant levels of the UMI-ONT-Seq analysis pipeline using either the default clustering or the cluster splitting strategy.

Kit	Algorithm	Mean noise reduction [%]	SD noise reduction [%]	Median noise reduction [%]	Samples with no noise with default clustering strategy	Samples with no noise with cluster splitting strategy
R9	HAC	82.7	96.4	72.8	5	10
V14	HAC	314.6	310.9	257.5	4	12
V14	SUP	232.8	108.8	204.9	4	12

Table S15: Variant levels and noise of variant level of the UMI-ONT-Seq analysis pipeline using the cluster splitting strategy.

Sample	Fragment	Kit	Algorithm	Mean variant level [%]	SD variant level [%]	Absolute variant level range observed [%]
100 % KIV-2B	PCR2645	R9	HAC	99.691	1.395	8.491
5%	PCR2645	R9	HAC	8.403	0.099	0.935
2.50%	PCR2645	R9	HAC	4.844	0.076	0.405
1.50%	PCR2645	R9	HAC	1.434	0.189	1.408
1%	PCR2645	R9	HAC	3.125	0	0
0.50%	PCR2645	R9	HAC	1.205	0	0
100 % KIV-2B	PCR5104	R9	HAC	99.987	0.049	0.195
5%	PCR5104	R9	HAC	4.415	0	0
2.50%	PCR5104	R9	HAC	0.948	0	0
1.50%	PCR5104	R9	HAC	1.274	0	0
1%	PCR5104	R9	HAC	1.153	0	0
0.50%	PCR5104	R9	HAC	0.188	0	0
100 % KIV-2B	PCR2645	V14	HAC	99.979	0.077	0.293
5%	PCR2645	V14	HAC	6.679	0.03	0.334
2.50%	PCR2645	V14	HAC	3.059	0.016	0.096
1.50%	PCR2645	V14	HAC	2.739	0.017	0.182
1%	PCR2645	V14	HAC	3.009	0	0
0.50%	PCR2645	V14	HAC	1.422	0.034	0.355
100 % KIV-2B	PCR5104	V14	HAC	99.989	0.052	0.381
5%	PCR5104	V14	HAC	4.085	0.029	0.227
2.50%	PCR5104	V14	HAC	2.467	0.02	0.112
1.50%	PCR5104	V14	HAC	1.515	0.016	0.126
1%	PCR5104	V14	HAC	0.99	0	0
0.50%	PCR5104	V14	HAC	0.326	0.088	0.621
100 % KIV-2B	PCR2645	V14	SUP	99.982	0.071	0.289
5%	PCR2645	V14	SUP	6.717	0.03	0.336
2.50%	PCR2645	V14	SUP	2.875	0.023	0.198
1.50%	PCR2645	V14	SUP	2.774	0.018	0.185
1%	PCR2645	V14	SUP	2.752	0	0
0.50%	PCR2645	V14	SUP	1.442	0.034	0.36
100 % KIV-2B	PCR5104	V14	SUP	99.996	0.018	0.083

5%	PCR5104	V14	SUP	3.9	0.028	0.216
2.50%	PCR5104	V14	SUP	2.37	0.037	0.273
1.50%	PCR5104	V14	SUP	1.289	0.014	0.107
1%	PCR5104	V14	SUP	1.802	0.082	0.901
0.50%	PCR5104	V14	SUP	0.306	0.039	0.301

Table S16: Threshold values for reaching a dataset Q-score of Q40. SD: Standard deviation

Kit	Algorithm	Result type	Minimal number of reads per cluster	Consensus sequences	Consensus sequences without error	Consensus sequences without error [%]	Mean Q-score per consensus	SD Q-score per consensus
R9	HAC	Raw	14	1220	747	61.23	38.336	2.41
V14	HAC	Raw	10	2488	1552	62.379	38.094	2.76
V14	SUP	Raw	6	3483	2174	62.417	38.168	2.681
R9	HAC	Transversion error corrected	12	1470	908	61.769	38.347	2.427
V14	HAC	Transversion error corrected	6	3271	2939	89.85	39.517	1.582
V14	SUP	Transversion error corrected	6	3483	3327	95.521	39.795	1.02

Table S17: Consensus sequence summary statistics for a minimal cluster size threshold of 6 reads per UMI cluster.

Kit	Algorithm	Total number of reads	Maximal number of errors	Reads with n or less errors	Percent of total reads	Number of bases[Gb]	Number of errors
R9	HAC	2997	0	1004	33.5	4.588	0
V14	HAC	3271	0	1901	58.117	7.487	0
V14	SUP	3483	0	2174	62.417	8.954	0
R9	HAC	2997	2	2111	70.437	9.132	1522
V14	HAC	3271	2	3168	96.851	12.619	1553
V14	SUP	3483	2	3425	98.335	14.061	1487

Table S18: Summary of the error profiles for all cluster thresholds greater than 10. ":" stands for insertion. "D" Deletion

Kit	Algorithm	Base change	Mean occurrence of error [%]	SD occurrence of error [%]
R9	HAC	:A	57.15%	26.58%
R9	HAC	AD	15.18%	20.32%
R9	HAC	CA	22.71%	20.02%
R9	HAC	GT	15.70%	21.24%

V14	HAC	CA	62.37%	5.69%
V14	HAC	GT	31.80%	8.81%
V14	SUP	CA	63.64%	5.27%
V14	SUP	GT	31.31%	7.22%

Table S19: Dataset Q-scores per sequencing chemistry and basecalling algorithm after error-profile adjustment.

Kit	Algorithm	Minimal number of reads per cluster	Cluster	Nucleotides	Errors	Dataset Q-score
R9	HAC	2	8639	35221384	724239	16.869
R9	HAC	4	4102	16375163	24585	28.235
R9	HAC	6	2997	12459002	5693	33.401
R9	HAC	8	2294	9974981	2315	36.344
R9	HAC	10	1827	8247966	1272	38.119
R9	HAC	12	1470	6902884	772	39.514
R9	HAC	14	1220	5833440	541	40.327
R9	HAC	16	1039	5049779	410	40.905
R9	HAC	18	882	4356647	321	41.326
R9	HAC	20	755	3769914	253	41.732
V14	HAC	2	11806	46875946	502112	19.701
V14	HAC	4	4415	18346483	5428	35.289
V14	HAC	6	3271	13082913	501	44.169
V14	HAC	8	2806	11019387	163	48.3
V14	HAC	10	2488	9679100	92	50.22
V14	HAC	12	2238	8636705	72	50.79
V14	HAC	14	2023	7743442	57	51.331
V14	HAC	16	1837	6995736	52	51.288
V14	HAC	18	1649	6232904	47	51.226
V14	HAC	20	1483	5587278	42	51.24
V14	SUP	2	13316	52615786	454575	20.635
V14	SUP	4	4825	20493221	2649	38.885
V14	SUP	6	3483	14295288	192	48.719
V14	SUP	8	2947	11852165	94	51.007
V14	SUP	10	2625	10464413	76	51.389
V14	SUP	12	2389	9468884	70	51.312
V14	SUP	14	2203	8708883	64	51.338
V14	SUP	16	2047	8082330	63	51.082
V14	SUP	18	1873	7388495	50	51.696
V14	SUP	20	1684	6618100	46	51.58

Table S20: Performance measures for all human gDNA samples for all sequencing chemistries and basecalling conditions.

Sample	Fragment	Kit	Algorithm	Raw reads	Consensus sequences	True positive	True negative	False positive	False negative	Sensitivity	Specificity	Precision	F1 score
EUR15415	5104	R9	HAC	70038	17	44	4973	4	83	0.346	0.999	0.917	0.503
EUR27513	5104	R9	HAC	88489	142	107	4978	9	10	0.915	0.998	0.922	0.918
EUR28877	5104	R9	HAC	156027	487	132	4966	4	2	0.985	0.999	0.971	0.978
EUR32880	5104	R9	HAC	151916	251	65	4971	3	65	0.5	0.999	0.956	0.657
EUR35729	5104	R9	HAC	95958	3	18	4999	1	86	0.173	1	0.947	0.293
EUR41297	5104	R9	HAC	128104	81	52	5031	19	2	0.963	0.996	0.732	0.832
EUR60915	5104	R9	HAC	110100	4	32	5037	0	35	0.478	1	1	0.646
EUR63113	5104	R9	HAC	157136	692	145	4942	16	1	0.993	0.997	0.901	0.945
EUR63453	5104	R9	HAC	114891	34	103	4980	3	18	0.851	0.999	0.972	0.907
EUR66900	5104	R9	HAC	93850	253	77	4943	22	62	0.554	0.996	0.778	0.647
EUR73204	5104	R9	HAC	208536	106	129	4868	105	2	0.985	0.979	0.551	0.707
EUR77692	5104	R9	HAC	147495	537	108	4990	4	2	0.982	0.999	0.964	0.973
EUR86619	5104	R9	HAC	97587	331	99	5003	0	2	0.98	1	1	0.99
EUR98778	5104	R9	HAC	114148	529	122	4976	2	4	0.968	1	0.984	0.976
EUR15415	5104	V14	HAC	107148	450	126	4975	1	2	0.984	1	0.992	0.988
EUR21943	5104	V14	HAC	115224	148	61	5039	1	3	0.953	1	0.984	0.968
EUR27513	5104	V14	HAC	90414	423	113	4985	2	4	0.966	1	0.983	0.974
EUR28877	5104	V14	HAC	106609	740	131	4970	0	3	0.978	1	1	0.989
EUR32880	5104	V14	HAC	103952	508	124	4974	0	6	0.954	1	1	0.976
EUR35729	5104	V14	HAC	100376	142	100	5000	0	4	0.962	1	1	0.98
EUR41297	5104	V14	HAC	114752	477	53	5048	1	2	0.964	1	0.981	0.972
EUR60915	5104	V14	HAC	101977	81	51	5029	8	16	0.761	0.998	0.864	0.81
EUR63113	5104	V14	HAC	100513	680	145	4943	14	2	0.986	0.997	0.912	0.948
EUR63453	5104	V14	HAC	99587	266	117	4982	1	4	0.967	1	0.992	0.979
EUR66900	5104	V14	HAC	112271	717	137	4962	2	3	0.979	1	0.986	0.982
EUR73204	5104	V14	HAC	157966	290	128	4967	6	3	0.977	0.999	0.955	0.966
EUR77692	5104	V14	HAC	102688	632	108	4994	0	2	0.982	1	1	0.991
EUR86619	5104	V14	HAC	116328	743	98	5002	1	3	0.97	1	0.99	0.98

EUR98778	5104	V14	HAC	159463	733	122	4978	0	4	0.968	1	1	0.984
EUR15415	5104	V14	SUP	109926	784	126	4976	0	2	0.984	1	1	0.992
EUR21943	5104	V14	SUP	117770	316	61	5040	0	3	0.953	1	1	0.976
EUR27513	5104	V14	SUP	106423	653	113	4987	0	4	0.966	1	1	0.983
EUR28877	5104	V14	SUP	108763	959	131	4970	0	3	0.978	1	1	0.989
EUR32880	5104	V14	SUP	106256	773	124	4974	0	6	0.954	1	1	0.976
EUR35729	5104	V14	SUP	102554	317	100	5000	0	4	0.962	1	1	0.98
EUR41297	5104	V14	SUP	117428	807	53	5049	0	2	0.964	1	1	0.981
EUR60915	5104	V14	SUP	104458	220	63	5036	1	4	0.94	1	0.984	0.962
EUR63113	5104	V14	SUP	119810	809	145	4945	12	2	0.986	0.998	0.924	0.954
EUR63453	5104	V14	SUP	101851	532	116	4983	0	5	0.959	1	1	0.979
EUR66900	5104	V14	SUP	138525	887	137	4964	0	3	0.979	1	1	0.989
EUR73204	5104	V14	SUP	261885	469	128	4966	7	3	0.977	0.999	0.948	0.962
EUR77692	5104	V14	SUP	124413	816	108	4994	0	2	0.982	1	1	0.991
EUR86619	5104	V14	SUP	125810	860	98	5003	0	3	0.97	1	1	0.985
EUR98778	5104	V14	SUP	177455	786	122	4978	0	4	0.968	1	1	0.984
EUR15415	5104	V14	SUPDUP	29849	7	30	4976	0	98	0.234	1	1	0.38
EUR28877	5104	V14	SUPDUP	30581	17	54	4969	1	80	0.403	1	0.982	0.571
EUR32880	5104	V14	SUPDUP	28577	8	31	4973	2	98	0.24	1	0.939	0.383
EUR41297	5104	V14	SUPDUP	34236	7	26	5048	1	29	0.473	1	0.963	0.634
EUR63113	5104	V14	SUPDUP	11125	27	124	4918	40	22	0.849	0.992	0.756	0.8
EUR86619	5104	V14	SUPDUP	12618	5	23	4999	4	78	0.228	0.999	0.852	0.359
EUR98778	5104	V14	SUPDUP	17411	81	118	4965	13	8	0.937	0.997	0.901	0.918

Table S21: Summary performance measures for UMI-ONT-Seq.

Kit	Algorithm	Samples	Sensitivity Mean	Sensitivity SD	Specificity Mean	Specificity SD	Precision Mean	Precision SD	F1 score Mean	F1 score SD
R9	HAC	14	0.762	0.288	0.997	0.005	0.9	0.128	0.784	0.214
V14	HAC	15	0.957	0.055	1	0.001	0.976	0.039	0.966	0.045
V14	SUP	15	0.968	0.013	1	0.001	0.99	0.023	0.979	0.011
V14	SUPDUP	7	0.481	0.298	0.998	0.003	0.913	0.086	0.578	0.221

Table S22: Summary performance measures for ONT-Seq without UMIs.

Kit	Algorithm	Samples	Sensitivity Mean	Sensitivity SD	Specificity Mean	Specificity SD	Precision Mean	Precision SD	F1 score Mean	F1 score SD
R9	HAC	14	0.614	0.3	0.595	0.191	0.106	0.221	0.073	0.058
V14	HAC	15	0.89	0.049	0.837	0.007	0.111	0.027	0.196	0.043
V14	SUP	15	0.799	0.317	0.889	0.021	0.132	0.06	0.225	0.1
V14	SUPDUP	8	0.95	0.031	0.971	0.008	0.399	0.122	0.554	0.123

Table S23: Differences and correlation values between ddPCR measured and UMI-ONT-Seq predicted number of KIV-2 repeats for different prediction strategies. CI: Confidence interval

Strategy	Mean difference	SD difference	r	R^2	Samples within ddPCR CI [n]
All unique haplotypes	+9.059	8.15	0.842	0.709	3
Noise-filtered haplotypes	-3.588	3.94	0.961	0.924	5
Coverage-corrected	+0.412	2.891	0.975	0.950	10

Table S24: Overview of the three trios, including number of consensus sequences, uniquely occurring haplotypes (used for the Venn diagram), the KIV-2 copy number estimation based on the coverage-corrected haplotypes and the results of the ddPCR. CI: Confidence interval.

Family ID	sample	Pop	Superpop	Relationship	Consensus sequences [n]	Filtered haplotypes [n]	KIV-2 repeats based on coverage-corrected haplotypes [n]	KIV-2 repeats based on ddPCR [n]	ddPCR CI min (95 %)	ddPCR CI max (95 %)
1	NA19238	YRI	AFR	mother	1779	33	35	32.23	30.9	33.6
1	NA19240	YRI	AFR	child	2355	29	32	29.6	31.4	27.8
1	NA19239	YRI	AFR	father	2484	18	22	22.04	21	23
2	NA19127	YRI	AFR	mother	3576	33	37	36.95	35.3	38.6
2	NA19129	YRI	AFR	child	1957	32	38	37.34	34.7	40
2	NA19128	YRI	AFR	father	3658	28	35	35.03	33.3	36.7
3	HG00657	CHS	EAS	mother	2357	29	30	31.43	29.8	33.1
3	HG00702	CHS	EAS	child	607	32	34	38.17	36.3	40
3	HG00656	CHS	EAS	father	2269	46	54	56.74	53.6	59.9

Table S25: KIV-2 subtype occurrence in the SAPHIR and 1000G dataset.

Population	Super Population	Samples	KIV-2 subtype	Subtype occurrence	Relative subtype occurrence
CDX	EAS	12	KIV-2B	2	0.167
CDX	EAS	12	KIV-2C	6	0.5
CDX	EAS	12	only KIV-2A	4	0.333
JPT	EAS	12	KIV-2B	4	0.333
JPT	EAS	12	KIV-2C	6	0.5
JPT	EAS	12	only KIV-2A	2	0.167
PJL	SAS	12	KIV-2B	6	0.5
PJL	SAS	12	KIV-2C	3	0.25
PJL	SAS	12	only KIV-2A	3	0.25
SAPHIR	EUR	15	KIV-2B	8	0.533
SAPHIR	EUR	15	KIV-2C	4	0.267
SAPHIR	EUR	15	only KIV-2A	3	0.2
YRI	AFR	12	KIV-2B	8	0.667
YRI	AFR	12	only KIV-2A	4	0.333

Table S26: KIV-2 subtype specific summary performance measures for UMI-ONT-Seq analysis of the 48 1000G samples.

Samples	KIV-2 subtype	Sensitivity Mean	Sensitivity SD	Specificity Mean	Specificity SD	Precision Mean	Precision SD	F1 score Mean	F1 score SD
35	contains KIV-2B and/or KIV-2C	0.995	0.007	1	0.001	0.983	0.022	0.989	0.013
13	only KIV-2A	0.592	0.047	1	0	0.988	0.009	0.74	0.037

Table S27: Per-sample performance measures for UMI-ONT-Seq analysis of the 48 1000G samples for V14 SUP.

Sample	Population	KIV-2 subtype	Raw reads	Consensus sequences	True positive	True negative	False positive	False negative	Sensitivity	Specificity	Precision	F1 score
HG00766	CDX	contains KIV-2B and/or KIV-2C	180551	937	114	4990	0	0	1	1	1	1
HG01802	CDX	contains KIV-2B and/or KIV-2C	212541	1272	143	4958	2	1	0.993	1	0.986	0.99

HG01811	CDX	contains KIV-2B and/or KIV-2C	310581	1802	114	4989	1	0	1	1	0.991	0.996
HG02154	CDX	contains KIV-2B and/or KIV-2C	203792	1269	143	4960	1	0	1	1	0.993	0.997
HG02184	CDX	contains KIV-2B and/or KIV-2C	200983	1190	145	4958	1	0	1	1	0.993	0.997
HG02375	CDX	contains KIV-2B and/or KIV-2C	149627	963	149	4954	1	0	1	1	0.993	0.997
HG02394	CDX	contains KIV-2B and/or KIV-2C	146497	684	108	4993	3	0	1	0.999	0.973	0.986
HG02406	CDX	contains KIV-2B and/or KIV-2C	231893	1410	136	4966	1	1	0.993	1	0.993	0.993
HG01805	CDX	only KIV-2A	218346	1313	58	5004	1	41	0.586	1	0.983	0.734
HG02355	CDX	only KIV-2A	172259	919	56	5003	1	44	0.56	1	0.982	0.713
HG02371	CDX	only KIV-2A	132332	883	63	4997	1	43	0.594	1	0.984	0.741
HG02385	CDX	only KIV-2A	202281	1191	60	4997	0	47	0.561	1	1	0.719
NA18942	JPT	contains KIV-2B and/or KIV-2C	191416	1052	131	4972	1	0	1	1	0.992	0.996
NA18945	JPT	contains KIV-2B and/or KIV-2C	185226	920	110	4994	0	0	1	1	1	1
NA18950	JPT	contains KIV-2B and/or KIV-2C	192303	1153	137	4965	2	0	1	1	0.986	0.993
NA18987	JPT	contains KIV-2B and/or KIV-2C	182461	839	146	4956	2	0	1	1	0.986	0.993
NA19007	JPT	contains KIV-2B and/or KIV-2C	182972	822	140	4960	0	4	0.972	1	1	0.986
NA19056	JPT	contains KIV-2B and/or KIV-2C	165790	904	112	4992	0	0	1	1	1	1
NA19058	JPT	contains KIV-2B and/or KIV-2C	75303	94	112	4983	8	1	0.991	0.998	0.933	0.961
NA19066	JPT	contains KIV-2B and/or KIV-2C	117345	471	149	4952	3	0	1	0.999	0.98	0.99
NA19072	JPT	contains KIV-2B and/or KIV-2C	115109	686	142	4960	2	0	1	1	0.986	0.993
NA19074	JPT	contains KIV-2B and/or KIV-2C	99583	556	102	5001	1	0	1	1	0.99	0.995
NA18944	JPT	only KIV-2A	132767	863	63	4993	2	46	0.578	1	0.969	0.724
NA19002	JPT	only KIV-2A	230404	1371	64	4997	1	42	0.604	1	0.985	0.749

HG02490	PJL	contains KIV-2B and/or KIV-2C	134239	754	107	4996	1	0	1	1	0.991	0.995
HG02652	PJL	contains KIV-2B and/or KIV-2C	94659	510	115	4986	2	1	0.991	1	0.983	0.987
HG02655	PJL	contains KIV-2B and/or KIV-2C	144664	632	95	5008	1	0	1	1	0.99	0.995
HG02684	PJL	contains KIV-2B and/or KIV-2C	139851	720	124	4969	10	1	0.992	0.998	0.925	0.958
HG02737	PJL	contains KIV-2B and/or KIV-2C	148915	931	115	4989	0	0	1	1	1	1
HG03021	PJL	contains KIV-2B and/or KIV-2C	131982	736	114	4989	1	0	1	1	0.991	0.996
HG03652	PJL	contains KIV-2B and/or KIV-2C	188154	837	127	4974	3	0	1	0.999	0.977	0.988
HG03653	PJL	contains KIV-2B and/or KIV-2C	158362	948	136	4967	1	0	1	1	0.993	0.996
HG03702	PJL	contains KIV-2B and/or KIV-2C	201185	1171	110	4993	1	0	1	1	0.991	0.995
HG02694	PJL	only KIV-2A	158195	1065	63	5000	1	40	0.612	1	0.984	0.754
HG03238	PJL	only KIV-2A	180456	1158	70	4982	1	51	0.579	1	0.986	0.729
HG03708	PJL	only KIV-2A	310158	1251	60	4997	1	46	0.566	1	0.984	0.719
NA18516	YRI	contains KIV-2B and/or KIV-2C	181110	1222	113	4990	1	0	1	1	0.991	0.996
NA18873	YRI	contains KIV-2B and/or KIV-2C	153257	774	142	4950	10	2	0.986	0.998	0.934	0.959
NA18907	YRI	contains KIV-2B and/or KIV-2C	347909	1941	160	4937	4	3	0.982	0.999	0.976	0.979
NA19092	YRI	contains KIV-2B and/or KIV-2C	264116	769	107	4995	1	1	0.991	1	0.991	0.991
NA19171	YRI	contains KIV-2B and/or KIV-2C	386363	1120	130	4960	12	2	0.985	0.998	0.915	0.949
NA19235	YRI	contains KIV-2B and/or KIV-2C	170053	706	144	4956	2	2	0.986	1	0.986	0.986
NA19239	YRI	contains KIV-2B and/or KIV-2C	181384	517	96	5006	0	2	0.98	1	1	0.99
NA19248	YRI	contains KIV-2B and/or KIV-2C	207469	1197	139	4962	1	2	0.986	1	0.993	0.989
NA18510	YRI	only KIV-2A	215364	1250	58	4989	0	57	0.504	1	1	0.671

NA18861	YRI	only KIV-2A	229313	1095	98	4964	0	42	0.7	1	1	0.824
NA18916	YRI	only KIV-2A	270688	1135	77	4984	1	42	0.647	1	0.987	0.782
NA19201	YRI	only KIV-2A	189399	922	73	4984	0	47	0.608	1	1	0.756

Table S28: Summary performance measures for UMI-ONT-Seq analysis of the 48 1000G samples without KIV-2B specific variants.

Samples	Sensitivity Mean	Sensitivity SD	Specificity Mean	Specificity SD	Precision Mean	Precision SD	F1 score Mean	F1 score SD
48	0.987	0.018	1	0.001	0.979	0.033	0.983	0.02

Table S29: Correlation between the number of KIV-2 repeats quantified by ddPCR and by UMI-ONT-Seq for each step of the UMI-ONT-Seq KIV-2 quantification algorithm in the 1000G dataset. The confidence interval (CI) is set to 95 %. CI: Confidence interval

Strategy	Mean difference	SD difference	r	R^2	Within ddPCR CI [n]
Unique	+3.833	7.413	0.68	0.462	10
Noise-filtered	-6.125	5.895	0.672	0.451	16
Coverage corrected	+0.292	4.371	0.851	0.724	32

Table S30: extracted STR sequences of all 63 human gDNA samples and two unmixed KIV-2A, respectively KIV-2B plasmids.

STR sequence	STR length	Occurrence (KIV-2 repeats) [n]
CACACACACACACACACACACACACACACACA	16	15504
CACACACACACACACACACACACACA	13	15367
CACACACACACACACACACACACACACACA	15	9701
CACACACACACACACACACACACACACA	14	6005
CACACACACACACACACACACACA	12	4056
CACACAGACACACACACACACACA	12	3844
CACACACACACACACACACA	10	2018
CACACACACACAGACACACACACACACACACA	16	1546
CACACACACACACACACACACACACACACACACA	17	1433
CACACACACACACACACACACA	11	1411
CACACACACACACACACACACACACACACACACACACACA	19	500
CACACACACACACACACACACACACACACACACACACACACA	20	429
CACACACACACACACACACACACACACACACACACA	18	414
CACACACACACACACACA	9	97
CACACAGACACACACACACACA	11	67
CACACACACACACACACACACACACACACACACACACACACACA	21	50
CACACACAAACACACACACACACACACACACACACA	18	33
CACACACAAACACACACACACACACACACACACACACA	19	33
CACACAAACACACACACACACACA	12	30
CACACAGACACACACACACACACACA	13	30
CACACAGACACACACACACACACACACA	14	27
CACACACAAACACACACACACACACACACACA	17	24
CACACACACACAGACACACACACACACA	15	22
CACACAGCACACACACACACACA	11.5	20
CACACACACACACACACACACACACACACACACACACACACACACA	22	4
CACACACACACAGCACACACACACACACACA	15.5	4
CACACACACAGACACACACACACACACACA	15	3
CACACAGACACACACACACA	10	3
CACACACAACACACACACACACACACACACACACA	17.5	2
CACACACACACACACA	8	2

Table S31: Population-specific degeneration of the STR region.

Population	Super population	Mutation	Mutation is at STR position	Occurrence [n samples]	Occurrence [n sequences]
YRI	AFR	none	-	12	12529
YRI	AFR	AA	8	1	90
YRI	AFR	A	2	1	3
CDX	EAS	none	-	12	12032
CDX	EAS	GA	7	9	1362
CDX	EAS	GA	15	9	417
CDX	EAS	G	7	4	13
JPT	EAS	none	-	12	8541
JPT	EAS	GA	7	8	729
JPT	EAS	GA	15	7	444
JPT	EAS	G	7	1	2
SAPHIR	EUR	none	-	15	12926
SAPHIR	EUR	GA	7	8	886
SAPHIR	EUR	GA	15	7	435
SAPHIR	EUR	AA	6	2	31
SAPHIR	EUR	G	7	2	5
SAPHIR	EUR	G	15	1	2
PJL	SAS	none	-	12	9426
PJL	SAS	GA	7	7	994
PJL	SAS	GA	15	6	272
PLASMID	EUR	none	-	2	1537

Table S32: Diversity of the extracted STR sequences per population.

Population	Super population	Unique STR sequences
PLASMID	EUR	4
CDX	EAS	18
YRI	AFR	18
PJL	SAS	20
JPT	EAS	22
SAPHIR	EUR	32

Table S33: Predicted number of KIV-2 repeats for 48 samples and the three trios of the 1000G project using either UMI-ONT-Seq, ddPCR or the results from Behera et al. [13].

Sample	Population	Super population	ddPCR predicted number of KIV-2 repeats	UMI-ONT-Seq predicted number of KIV-2 repeats	Behera et al predicted number of KIV-2 repeats
NA18510	YRI	AFR	37	35	38
NA18873	YRI	AFR	31	29	31
NA18861	YRI	AFR	35	35	37
NA19092	YRI	AFR	24	26	27
NA19235	YRI	AFR	35	36	37
NA19201	YRI	AFR	22	22	23
NA18916	YRI	AFR	33	35	35
NA19171	YRI	AFR	33	34	34
NA19248	YRI	AFR	36	38	38
NA19239	YRI	AFR	20	23	23
NA18907	YRI	AFR	36	38	37
NA18516	YRI	AFR	27	32	31
HG02371	CDX	EAS	48	42	50
HG02385	CDX	EAS	45	42	48
HG02355	CDX	EAS	34	32	35
HG00766	CDX	EAS	47	49	52
HG02375	CDX	EAS	47	48	50
HG02406	CDX	EAS	41	38	40
HG02184	CDX	EAS	42	47	49
HG01811	CDX	EAS	52	53	54
HG01805	CDX	EAS	47	48	49
HG02394	CDX	EAS	34	36	36
HG01802	CDX	EAS	38	41	41
HG02154	CDX	EAS	51	52	52
NA19074	JPT	EAS	43	23	45
NA19058	JPT	EAS	40	30	48
NA19056	JPT	EAS	50	41	48
NA18950	JPT	EAS	43	44	48
NA19002	JPT	EAS	52	50	53
NA19007	JPT	EAS	39	37	40
NA18987	JPT	EAS	41	40	42
NA19066	JPT	EAS	43	44	45
NA18945	JPT	EAS	38	42	42
NA18942	JPT	EAS	41	43	43
NA18944	JPT	EAS	42	46	46
NA19072	JPT	EAS	46	50	47
HG03021	PJL	SAS	37	38	40
HG03708	PJL	SAS	43	44	46
HG03653	PJL	SAS	41	41	42

HG02490	PJL	SAS	34	34	35
HG03652	PJL	SAS	36	41	41
HG02694	PJL	SAS	41	43	43
HG02655	PJL	SAS	26	28	28
HG02684	PJL	SAS	32	33	33
HG02737	PJL	SAS	28	31	30
HG03238	PJL	SAS	42	44	43
HG03702	PJL	SAS	45	51	50
HG02652	PJL	SAS	30	33	32
NA19238	YRI	AFR	35	32	35
NA19240	YRI	AFR	32	30	32
NA19239	YRI	AFR	22	22	23
NA19127	YRI	AFR	37	37	36
NA19129	YRI	AFR	38	37	38
NA19128	YRI	AFR	35	35	35
HG00657	CHS	EAS	30	31	32
HG00702	CHS	EAS	34	38	39
HG00656	CHS	EAS	54	57	56

Table S34: Number of KIV unit specific sequence patterns found in the raw reads of the 48 1000 Genome samples.

Pattern	Total reads [n]	Total Samples [n]	Total hits [n]	Mean hits [%]
**KIV-1**	**54117642**	**288**	**1**	**0.00%**
AGAAAACATGAATATAGAAT	9019607	48	1	0.00%
ATCTATTCCTTTGGTTTTTT	9019607	48	0	0.00%
CAGTGCCAAATGTATCTCCC	9019607	48	0	0.00%
GGTGCTAGATGCAAAACCAT	9019607	48	0	0.00%
TGACATATCACTATATAATA	9019607	48	0	0.00%
TGCTGGGCACAAGAAAATTT	9019607	48	0	0.00%
**KIV-3**	**45098035**	**240**	**1323**	**0.00%**
ACTGAAGTTTCTGTGACAAG	9019607	48	344	0.00%
CCAATAGGGAGAAAATCTCT	9019607	48	333	0.00%
CCTTGCCCAACACCCACTAA	9019607	48	341	0.00%
CTCTGGTATTTTCTAGGTCC	9019607	48	159	0.00%
TCAGATTCAGATGGTGTAGC	9019607	48	146	0.00%
**KIV-4**	**45098035**	**240**	**0**	**0.00%**
AGAAAACATGAATACGGAAT	9019607	48	0	0.00%
CAAATGAGAATGCTACATCC	9019607	48	0	0.00%
CTGCAGGGGAGTAAGCTGTT	9019607	48	0	0.00%
GTATGAACCATGACCAGGTG	9019607	48	0	0.00%
GTGGGTCATGAGAGGATTTC	9019607	48	0	0.00%

References

1. Coassin S, Schönherr S, Weissensteiner H, Erhart G, Forer L, Losso JL, et al. **A comprehensive map of single-base polymorphisms in the hypervariable LPA kringle IV type 2 copy number variation region.** *J Lipid Res* 2019;60(1)**:**186-199.
2. Heid IM, Wagner SA, Gohlke H, Iglseder B, Mueller JC, Cip P, et al. **Genetic architecture of the APM1 gene and its influence on adiponectin plasma levels and parameters of the metabolic syndrome in 1,727 healthy Caucasians.** *Diabetes* 2006;55(2)**:**375-384.
3. Lanktree MB, Rajakumar C, Brunt JH, Koschinsky ML, Connelly PW, Hegele RA. **Determination of lipoprotein(a) kringle repeat number from genomic DNA: copy number variation genotyping using qPCR.** *J Lipid Res* 2009;50(4)**:**768-772.
4. Hindson BJ, Ness KD, Masquelier DA, Belgrader P, Heredia NJ, Makarewicz AJ, et al. **High-Throughput Droplet Digital PCR System for Absolute Quantitation of DNA Copy Number.** *Anal Chem* 2011;83(22)**:**8604-8610.
5. Madeira F, Madhusoodanan N, Lee J, Eusebi A, Niewielska A, Tivey ARN, et al. **The EMBL-EBI Job Dispatcher sequence analysis tools framework in 2024.** *Nucleic Acids Res* 2024;52(W1)**:**W521-W525.
6. Coassin S, Kronenberg F. **Lipoprotein(a) beyond the kringle IV repeat polymorphism: The complexity of genetic variation in the LPA gene.** *Atherosclerosis* 2022;349**:**17-35.
7. Coassin S, Erhart G, Weissensteiner H, de Arajo MEG, Lamina C, Schönherr S, et al. **A novel but frequent variant in LPA KIV-2 is associated with a pronounced Lp(a) and cardiovascular risk reduction.** *Eur Heart J* 2017;38(23)**:**1823-1831.
8. Weissensteiner H, Forer L, Fuchsberger C, Schöpf B, Kloss-Brandstätter A, Specht G, et al. **mtDNA-Server: next-generation sequencing data analysis of human mitochondrial DNA in the cloud.** *Nucleic Acids Res* 2016;44(W1)**:**W64-W69.
9. Garrison E, Marth G. **Haplotype-based variant detection from short-read sequencing.** *ArXiv [Preprint]* 2012**:**arXiv:1207.3907.
10. Wilm A, Aw PPK, Bertrand D, Yeo GHT, Ong SH, Wong CH, et al. **LoFreq: A sequence-quality aware, ultra-sensitive variant caller for uncovering cell-population heterogeneity from high-throughput sequencing datasets.** *Nucleic Acids Res* 2012;40(22)**:**11189-11201.
11. Byrska-Bishop M, Evani US, Zhao X, Basile AO, Abel HJ, Regier AA, et al. **High-coverage whole-genome sequencing of the expanded 1000 Genomes Project cohort including 602 trios.** *Cell* 2022;185(18)**:**3426-3440.e19.
12. Noureen A, Fresser F, Utermann G, Schmidt K. **Sequence Variation within the KIV-2 Copy Number Polymorphism of the Human LPA Gene in African, Asian, and European Populations.** *PLoS One* 2015;10(3)**:**e0121582.
13. Behera S, Belyeu JR, Chen X, Paulin LF, Nguyen NQH, Newman E, et al. **Identification of allele-specific KIV-2 repeats and impact on Lp(a) measurements for cardiovascular disease risk.** *bioRxiv* 2023**:**2023.04.24.538128.
